# Factors in the Initial Resuscitation of Patients With Severe Trauma: The FiiRST-2 Randomized Clinical Trial

**DOI:** 10.1001/jamanetworkopen.2025.32702

**Published:** 2025-09-22

**Authors:** Luis T. da Luz, Keyvan Karkouti, Jo Carroll, Deep Grewal, Marti Jones, Jennifer Altmann, Yulia Lin, Akash Gupta, Avery B. Nathens, Amie Kron, Lowyl Notario, Andrew Beckett, Andrew Petrosoniak, Katerina Pavenski, Kelly Vogt, Ziad Solh, Ian M. Ball, Neil G. Parry, Paul T. Engels, Michelle P. Zeller, Donald M. Arnold, Emilie Belley-Côté, Chris Evans, Jordan Leitch, Andrew Shih, Philip Jean Dawe, Robert Gooch, Jeannie Callum

**Affiliations:** 1 Division of General Surgery, Sunnybrook Health Sciences Centre, Toronto, Ontario, Canada; 2 Department of Surgery, University of Toronto, Toronto, Ontario, Canada; 3 Department of Anesthesiology and Pain Medicine, University Health Network, Sinai Health System, Women’s College Hospital, Toronto, Ontario, Canada; 4 Department of Anesthesiology and Pain Medicine, University Health Network, Toronto, Ontario, Canada; 5 Biostatistics, ERGOMED PLC, Guildford, United Kingdom; 6 ERGOMED PLC, Cologne, Germany; 7 Precision Diagnostics and Therapeutics Program, Sunnybrook Health Sciences Centre, Toronto, Ontario, Canada; 8 Tory Trauma Program, Sunnybrook Health Sciences Centre, Toronto, Ontario, Canada; 9 General Surgery, Unity Health Toronto, Toronto, Ontario, Canada; 10 Emergency Medicine, Unity Health Toronto, Toronto, Ontario, Canada; 11 Transfusion Medicine, Unity Health Toronto, Toronto, Ontario, Canada; 12 General Surgery, London Health Sciences Centre, London, Ontario, Canada; 13 Western University, London, Ontario, Canada; 14 Division of Transfusion Medicine, Department of Pathology and Laboratory Medicine, Schulich School of Medicine and Dentistry, Western University, London, Ontario, Canada; 15 Division of Hematology, Department of Medicine, Schulich School of Medicine and Dentistry, Western University, London, Ontario, Canada; 16 Critical Care Medicine and Office of Academic Military Medicine, London Health Sciences Centre Trauma Program, London, Ontario, Canada; 17 Critical Care Trauma Center, London Health Sciences Centre, London, Ontario, Canada; 18 General Surgery, Hamilton General Hospital, Hamilton, Ontario, Canada; 19 McMaster University, Hamilton, Ontario, Canada; 20 Michael G. DeGroote Centre for Transfusion Research, Department of Medicine, Hamilton, Ontario, Canada; 21 Cardiology and Critical Care, McMaster University, Hamilton, Ontario, Canada; 22 Emergency Medicine, Kingston General Hospital, Kingston, Ontario, Canada; 23 Queen’s University, Kingston, Ontario, Canada; 24 Anesthesia and Perioperative Medicine, Kingston General Hospital, Kingston, Ontario, Canada; 25 Transfusion Medicine, Vancouver General Hospital, Vancouver, British Columbia, Canada; 26 University of British Columbia, Vancouver, British Columbia, Canada; 27 Acute Care and Trauma Surgery, Vancouver General Hospital, Vancouver, British Columbia, Canada; 28 Emergency Medicine, Vancouver General Hospital, Vancouver, British Columbia, Canada; 29 Pathology and Molecular Medicine, Kingston General Hospital, Kingston, Ontario, Canada

## Abstract

**Importance:**

Patients with bleeding and coagulopathic trauma often require more transfusions and have higher mortality rates, motivating research on improving hemostatic strategies.

**Objective:**

To evaluate the replacement of clotting factors with frozen plasma (FP) or factor concentrates (fibrinogen concentrate [FC] and prothrombin complex concentrate [PCC]) in the initial resuscitation of patients with trauma.

**Design, Setting, and Participants:**

This multicenter, parallel-control, superiority randomized clinical trial was conducted at 6 level I trauma centers in Canada, between April 2021 and February 2023. Eligible patients were those with massive hemorrhage protocol (MHP) activation on admission and aged 16 years or older. Patients were excluded if they received more than 2 red blood cell (RBC) units either before hospital admission or in the hospital prior to randomization or if they had a catastrophic head injury. Follow-up was completed on March 25, 2023. The primary analysis was based on the modified intention-to-treat approach.

**Interventions:**

The intervention group received FC 4 g and PCC 2000 IU in MHP packs 1 and 2. The control group received 4 FP units. Concurrently, patients received 4 RBC units (both packs) and 1 dose of platelets (pack 2). After pack 2, FP was administered at clinician discretion.

**Main Outcomes and Measures:**

Primary outcome was the number of allogeneic blood product (RBC, FP, and platelet) units administered within 24 hours. Secondary outcomes included incidence of thromboembolic events, duration of intensive care unit stay, and mortality.

**Results:**

Of the 217 patients enrolled, 107 were randomly assigned to the FC-PCC group and 110 to the FP group; 137 patients were included in the primary analysis (66 in the FC-PCC group and 71 in the FP group). Baseline characteristics were similar between groups (median [IQR] age, 38 [29-55] years; 111 males [81.0%]). Among these patients, 95 (69.3%) had blunt mechanisms of injury, and the median (IQR) Injury Severity Score was 29 (19-43). Mean 24-hour transfusions were 20.8 (95% CI, 16.7-25.9) units in the FC-PCC group and 23.8 (95% CI, 19.2-29.4) units in the FP group. The mean ratio was 0.87 (1-sided 97.5% CI, 0.00-1.19; *P* = .20 for superiority). No significant differences were found in thromboembolic complications or 24-hour and 28-day mortality. The trial was terminated after the interim analysis showed conditional power of less than 25%, requiring an impractically large sample to show superiority.

**Conclusions and Relevance:**

In this randomized clinical trial, clotting factor concentrates were not superior to FP for initial resuscitation of patients with trauma. Efficacy and safety outcomes were similar across the treatment groups.

**Trial Registration:**

ClinicalTrials.gov Identifier: NCT04534751

## Key Points

**Question:**

Do fibrinogen concentrate (FC) and prothrombin complex concentrate (PCC) reduce allogeneic blood product use within 24 hours in severely injured patients with trauma compared with frozen plasma (FP)?

**Findings:**

In this randomized clinical trial involving 137 patients with massive hemorrhage protocol activation on admission, there was no significant difference in 24-hour allogeneic blood product use between those in the FC-PCC group vs the FP group.

**Meaning:**

FC and PCC were not superior to FP for initial resuscitation of patients with trauma.

## Teaser

This randomized clinical trial evaluates whether fibrinogen concentrate and prothrombin complex concentrate reduce the number of allogenic blood product units administered within 24 hours compared with plasma in patients requiring massive hemorrhage protocol activation.

## Introduction

Trauma-induced coagulopathy (TIC) is common in critically injured patients.^1^ Its complex pathophysiological mechanism^2^ raises uncertainty about using an aggressive hemostatic approach and whether to apply preventive or reactive transfusion strategies. Coagulation tests may take up to 60 minutes, delaying treatment.^3^ Even with viscoelastic testing, delays occur^4^; thus, initial resuscitation decisions rely on clinical information.

In Europe, clotting factor concentrates (fibrinogen concentrate [FC] and prothrombin complex concentrate [PCC]) are used for TIC treatment,^5^ while North America mainly uses ratio-based plasma resuscitation.^3,6^ Factor concentrates have advantages over plasma: they are easier and faster to administer, have a longer shelf life, can be stored at room temperature, require no additional preparation equipment, and can be used in remote regions. They do not need blood grouping, are pathogen reduced, and eliminate the need for prethawed AB plasma.

Hospitals should have massive hemorrhage protocols (MHPs) to standardize blood product delivery for patients with suspected TIC during initial resuscitation.^3,5,6^ These protocols vary by resources: high-volume centers maintain thawed plasma, medium centers thaw on demand, and rural sites may lack storage or thawing capacity. This variation leads some regions to use FC and PCC as a bridge until transfer to a trauma center.^3,6^

The impact on transfusion volumes, 24-hour mortality, and thromboembolic complications reported by current randomized clinical trials (RCTs) assessing factor concentrates in trauma is inconclusive.^7,8,9^ We hypothesized that using FC and PCC compared with plasma in the first 2 transfusion packs for severely injured patients with trauma and MHP activation would reduce total allogeneic blood products (ABPs) transfused at 24 hours. Thus, we conducted a trial to evaluate the replacement of clotting factors with frozen plasma (FP) or FC and PCC in the initial resuscitation of patients with trauma.

## Methods

### Study Design

This multicenter, parallel-control, superiority RCT—Factor in the Initial Resuscitation of Severe Trauma 2 Patients (FiiRST-2)—was conducted at 6 level I trauma centers in Canada, between April 2021 and February 2023. The trial protocol has been published,^10^ and no changes were made following patient enrollment; the protocol and statistical analysis plan are provided in Supplements 1 and 2. The trial was coordinated by the Clinical Trials Unit at the University Health Network and was conducted in accordance with the principles of the Declaration of Helsinki,^11^ Good Clinical Practice Guidelines, and applicable regulations. The Sunnybrook Health Sciences Centre Research Ethics Board approved the study before patient enrollment, with review coordinated through Clinical Trials Ontario. Each participating site obtained local research ethics board approval. Written informed consent was obtained from the patient or substitute decision-maker after treatment^12,13^ (the consent process occurred after treatment due to the emergency nature of trauma). An independent data and safety monitoring committee (IDSMC) oversaw the trial. We followed the Consolidated Standards of Reporting Trials (CONSORT) reporting guideline.

Patients (aged ≥16 years) with traumatic injury requiring MHP activation by the criteria at participating sites (eTable 1 in Supplement 3) within 1 hour of hospital admission were eligible. Blood bank technologists confirmed eligibility and randomly assigned patients to treatment groups while applying the following exclusion criteria: received more than 2 units of red blood cells (RBCs) before hospital admission or in the hospital prior to MHP activation; had more than 3 hours from injury; had a penetrating brain injury with a Glasgow Coma Scale score of 3 (score range: 3-15, with higher scores indicating greater disability); taken anticoagulants within the past 7 days; had a bleeding disorder; were pregnant; refused blood transfusion; or had a history of heparin-induced thrombocytopenia. Race and ethnicity were collected as reported by the patient and/or substitute decision-maker (including Black, East Asian, Latino, Middle Eastern, South Asian, White, and not known). These variables were assessed to evaluate potential demographic differences between groups and to support future analyses of equity in trauma care and transfusion outcomes.

### Interventions

#### Trial Procedures

**Figure 1.**
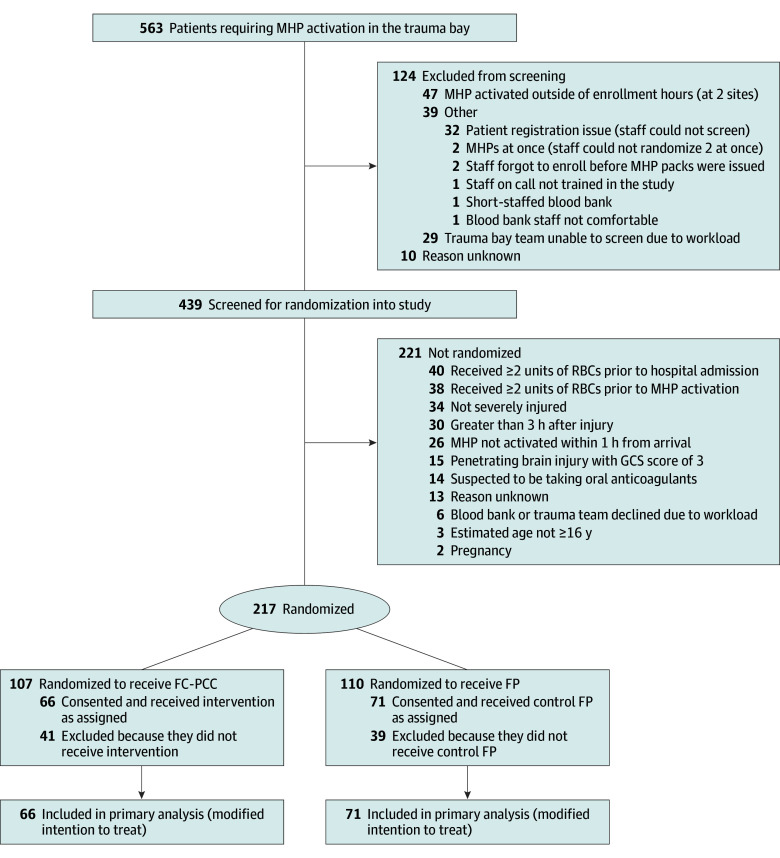
Flow Diagram of FiiRST-2 Trial Participants FC-PCC indicates fibrinogen concentrate and prothrombin complex concentrate; FP, frozen plasma; GCS, Glasgow Coma Scale (score range: 3-15, with higher scores indicating greater disability); MHP, massive hemorrhage protocol; RBCs, red blood cells.

A 1:1 randomization was performed using a permuted-block random allocation schedule, stratified by center, generated by an independent statistician (M.J.). Patients were randomly assigned to receive the intervention FC and PCC (hereafter FC-PCC) or the control FP (Figure 1). Sealed, opaque, and consecutively numbered envelopes were provided to centers. Clinicians remained blinded to treatment allocation with a tamper-proof seal on the assigned products until the decision to administer coagulation factors.

In the intervention group, patients were provided FC 4 g (Fibryga; Octapharma) and PCC 2000 IU (Octaplex; Octapharma) in the first and second MHP packs. In the control group, patients were provided 4 units of FP in both packs. The doses of intravenous FC-PCC were consistent with those in the literature.^5,9^ FC was also permitted in the control group if fibrinogen level was below 1.5 to 2.0 g/L. However, PCC was not permitted in the control group during the intervention period (first 24 hours or until after administration of the 2 MHP packs).

Both groups received 4 units of RBCs with the first and second coagulation factor packs and a single dose of platelets (4 units) with the second pack. Clinicians decided when to release and infuse pack components, but they had to administer all FC-PCC or FP in the first pack before moving to the second pack. If the second pack was opened, all FC-PCC or FP had to be given before starting the third pack. Not complying with this trial requirement was considered a trial protocol deviation. This strategy aimed to maintain compliance with RBC: plasma ratio not greater than 2:1. The order of infusion within each MHP pack (ie, RBC and plasma or FC-PCC) was left to the discretion of the treating team based on the clinical context. Although alternating RBC and plasma administration is common practice in Canadian trauma centers, this sequence was not mandated in the trial protocol, as there is no trial evidence to support a standardized order.

The second MHP pack (if required) had to be administered within 24 hours of arrival or MHP termination, whichever occurred first. Additional packs were transfused according to existing site MHPs, using plasma for factor replacement. After achieving hemorrhage control and MHP termination, transfusions were guided by laboratory test results. All sites intravenously administered 2 g of tranexamic acid within 3 hours of injury. The treatment period spanned randomization to 24 hours or after the second MHP pack, whichever occurred first.

#### Outcomes

Efficacy and safety data were collected through day 28. The primary efficacy outcome was the mean number of ABPs transfused within 24 hours. The key secondary efficacy outcome was the mean number of ABPs transfused within 24 hours, excluding FP units in the first 2 MHP packs. Other outcomes included individual blood component units transfused within 24 hours, rescue use of hemostatic agents (recombinant factor VIIa, PCC, and FC) from the third MHP pack onward, laboratory end points, days out of hospital to 28 days, 24-hour and all-cause 28-day mortality, and time to death (to 28 days). Follow-up was completed on March 25, 2023.

#### Safety

The key secondary safety outcomes were the incidence of thromboembolic events (TEEs) up to 28 days, ventilator-free days, duration of intensive care unit stay, multiorgan failure, abdominal compartment syndrome, and limb compartment syndrome. TEEs were screened by the blinded research personnel, the blinded investigator, and the monitors. TEEs were classified based on the medical records by the bedside team, supplemented by radiologic and other information. Medical records abstraction was identical for both treatment arms. Severe adverse events (AEs) were recorded and reported to the IDSMC by the principal investigators. In addition, independent trial monitors screened all medical records for AEs.

### Sample Size Estimation

The power and sample size calculations were based on a 2-sample, 1-sided test for the ratio of means. While the primary analysis ultimately used a negative binomial model, the initial power calculation was performed assuming approximate normality of the mean number of ABP units. A sample size of 297 was determined to have 80% power to detect superiority of the primary outcome, assuming a Type I error probability of α = .025 and mean difference of 5 ABP units. The FiiRST-2 steering committee considered this difference achievable and clinically meaningful to clinicians. Empirical estimates of the mean (SD) number of ABP units within the first 24 hours and their dispersion were based on results of the FiiRST-1 pilot study^14^: 1.50 (2.51) units in the intervention group, and 3.06 (5.06) units in the control group. The sample estimate was inflated to 350 units (175 units per arm) to account for a dropout percentage of 15%.

### Statistical Analysis

Results were expressed as means (SDs) for normally distributed variables, medians (IQRs) for non-normally distributed variables, and numbers (percentages). Categorical variables were compared with the χ^2^ test or, if not applicable, the Fisher exact test. No missing outcomes were imputed. The primary analysis, which used a modified intention-to-treat (mITT) approach, excluded randomly assigned patients who were not treated because it is frequent in trauma for an MHP to be activated and then quickly deactivated. The bedside tools that clinicians use to estimate the need for an MHP have low sensitivity and specificity, frequently leading to unnecessary activations. There have been previous cardiac surgery trials assessing hemostasis using the mITT design (FIBRES and FARES 2).^15,16^ Sensitivity analyses were conducted for the per-protocol population, which excluded patients who received less than 50% of the factor replacement in pack 1 and patients with major protocol deviations.

Superiority was determined with a negative binomial regression model for count data using a log link function with treatment effect, with treatment group as the main effect. Test hypotheses were H0 (null hypothesis) represented by a ratio of event rates (RR) greater than or equal to 1 vs Ha (alternative hypothesis of a true effect of difference) represented by an RR less than 1, with RR as the ratio of event rates λ2/λ1, where λ1 and λ2 represent the mean number of ABPs in the control and intervention groups, respectively. Inferences were based on the 1-sided 97.5% CI for the ratio λ2/λ1 derived from the model’s estimated least-squares means (LSM). Superiority is concluded if the upper CI limit is less than 1.0.

Survival differences between treatment groups were estimated using the risk ratio with 95% CI. Kaplan-Meier estimators were used to calculate and graphically present the time to death distribution. Safety outcomes included all randomly assigned patients who received any interventional products and consented to remain in the study. Safety analyses focused on treatment-emergent AEs, defined as AEs that start or worsen after treatment (in intervention or active control group) began.

The study followed a group sequential design with the Fleming error-spending function,^17^ a futility boundary (conditional power <25%), and sample size reestimation with conditional power based on the observed data from the first 120 patients. The test statistic derived from the negative binomial regression model planned for the primary analysis was used, which included the observed effect size and variance. An interim analysis was performed by an independent statistician (M.J. or J.A.) after 120 patients were treated, provided consent, and completed the 28-day follow-up.

Two-sided *P* < .05 indicated statistical significance. All data handling and statistical analyses were performed by independent statisticians (M.J. and J.A.) from November 2022 to June 2025 using SAS software, version 9.4 (SAS Institute Inc).

## Results

Among 563 patients requiring MHP activations, 439 were screened for enrollment (Figure 1). Of these, 217 patients were enrolled and randomly assigned (107 to the FC-PCC intervention group and 110 to the FP control group). The intervention MHP pack was opened and administered to 66 patients (61.7%), and FP was provided to 71 patients (64.5%) in the control group, resulting in 137 patients included in the mITT primary analysis. All patients in the FP group completed the 28-day study period, but 2 patients in the FC-PCC group could not be contacted (1 was discharged to home on day 8, and 1 was discharged to rehabilitation on day 24).

The trial was stopped after interim analysis demonstrated power less than 25%, requiring an impractically large sample size to detect superiority. Overall, both groups had similar characteristics (Table 1). The 137 patients in the mITT analysis consisted of 26 females (19.0%) and 111 males (81.0%) with a median (IQR) age of 38 (29-55) years, among whom 10 (7.3%) identified as Black, 10 (7.3%) as East Asian, 4 (2.9%) as Latino, 5 (3.7%) as Middle Eastern, 24 (17.5%) as South Asian, 42 (30.7%) as White, and 42 (30.7%) with unknown race and ethnicity. Among these patients, the median (IQR) Injury Severity Score (ISS) was 29 (19-43) (33 [18-45] in the FC-PCC group and 29 [22-43] in the control group) and 95 (69.3%) had blunt mechanisms of injury. The median (IQR) systolic blood pressure (SBP) on admission was 108 (93-138) mm Hg in the FC-PCC group and 127 (85-152) mm Hg in the control group; 54 patients (39.4%) had a SBP on admission lower than 90 mm Hg, and 63 (45.9%) had an Assessment of Blood Consumption score of 2 or higher.

**Table 1.** Characteristics of Trial Participants in the Primary Analysis

Characteristic	Patients		
	All (n = 137)	FP group (n = 71)	FC-PCC group (n = 66)
Demographics			
Age, median (IQR), y	38 (29-55) [n = 135]	41 (31-55) [n = 69]	35 (27-53) [n = 66]
Sex, No. (%)			
Female	26 (19.0)	15 (21.1)	11 (16.7)
Male	111 (81.0)	56 (78.9)	55 (83.3)
BMI, median (IQR), [No.]	27 (23-31) [n = 95]	27 (23-32) [n = 51]	27 (24-29) [n = 44]
Race and ethnicity^a^			
Black	10 (7.3)	6 (8.4)	4 (6.1)
East Asian	10 (7.3)	7 (9.9)	3 (4.5)
Latino	4 (2.9)	2 (2.8)	2 (3.0)
Middle Eastern	5 (3.7)	2 (2.8)	3 (4.6)
South Asian	24 (17.5)	14 (19.7)	10 (15.1)
White	42 (30.7)	19 (26.8)	23 (34.9)
Not known	42 (30.7)	21 (29.6)	21 (31.8)
Injury data			
Mechanism, No. (%)			
Blunt	95 (69.3)	47 (66.2)	48 (72.7)
Penetrating	46 (33.6)	25 (35.2)	21 (31.8)
Injury severity			
ISS, median (IQR)^b^	29 (19-43) [n = 136]	29 (22-43) [n = 70]	33 (18-45) [n = 66]
ISS ≥15, No. (%)	117 (85.4)	63 (88.7)	54 (81.8)
Head AIS ≥3, No. (%)^c^	41 (30.1) [n = 136]	19 (27.1) [n = 70]	22 (33.3) [n = 66]
GCS, median (IQR)^d^	13 (5-15)	10 (3-15)	14 (5-15)
Mild: score >13	69 (50.4)	29 (40.8)	40 (60.6)
Moderate: score 9-12	16 (11.7)	12 (16.9)	4 (6.1)
Severe: score 0-8	50 (36.5)	30 (42.3)	20 (30.3)
Physiologic data on admission			
HR per min			
Median (IQR)	113 (92-131) [n = 132]	111 (88-129) [n = 68]	113 (95-134) [n = 64]
>120 per min, No. (%)	55 (40) [n = 132]	28 (39) [n = 68]	27 (41) [n = 64]
SBP, mm Hg			
Median (IQR)	114 (89-148) [n = 126]	127 (85-152) [n = 65]	108 (93-138) [n = 61]
≤90 mm Hg	34 (24.8)	20 (28.2)	14 (21.2)
ABC score ≥2^e^	63 (45.9)	35 (49.3)	28 (42.4)
SI >1.0^f^	54 (39.4)	25 (35.2)	29 (43.9)
Laboratory data on admission			
Blood pH, median (IQR)^g^	7.2 (7.2-7.3)	7.2 (7.2-7.3)	7.2 (7.2-7.3)
Base deficit, median (IQR), mEq/L^g^	8 (4-11)	8 (4-11)	8 (4-10)
Lactate, median (IQR), mg/dL^g^	45 (36-63)	45 (36-63)	45 (36-72)
Hemoglobin, median (IQR), g/dL^g^	12.1 (10.7-133)	11.6 (10.2-13.3)	12.1 (11.1-13.1)
INR, median (IQR)^h^	1.3 (1.2-1.5)	1.3 (1.2-1.6)	1.3 (1.2-1.5)
INR >1.2, No. (%)	73 (53.3)	37 (52.1)	36 (54.5)
INR >1.5, No. (%)	24 (17.5)	13 (18.3)	11 (16.7)
Fibrinogen, median (IQR), mg/dL^g^	180 (103-240)	170 (120-240)	190 (140-240)
Fibrinogen ≤1.5 mg/dL), No. (%)	29 (21.2)	17 (23.9)	12 (18.2)
Platelet count, median (IQR), ×10^3^/µL^g^	215 (155-270)	220 (149-226)	208 (162-263)
Resuscitation indicators			
Transfusion of ≥3 RBC units within the first h	112 (81.8)	60 (84.5)	52 (78.8)
Transfusion of ≥10 RBC units within the first 24 h	53 (38.7)	24 (33.8)	29 (43.9)
Tranexamic acid infused	123 (89.8)	64 (90.1)	59 (89.4)
Time from injury to TB or ED, median (IQR), min	46.5 (35-63) [n = 96]	52 (34-66) [n = 51]	45 (36-55) [n = 45]
Time from arrival to start of IMP, median (IQR), min	49 (38-68)	41 (31-68)	52 (40-68)
Need for hemostasis control procedure (surgical or radiologic), No (%)	101 (73.7)	58 (81.7)	43 (65.1)

Abbreviations: ABC, assessment of blood consumption; AIS, Abbreviated Injury Scale; BMI, body mass index (calculated as weight in kilograms divided by height in meters squared); ED, emergency department; FC, fibrinogen concentrate; FP, frozen plasma; GCS, Glasgow Coma Scale; HR, heart rate; IMP, investigational medicinal product; INR, International Normalized Ratio; ISS, Injury Severity Score; PCC, prothrombin complex concentrate; RBCs red blood cell; SBP, systolic blood pressure; SI, Shock Index; TB, trauma bay.

SI conversion factor: To convert base deficit to millimoles per liter, multiply by 1.0; fibrinogen to grams per liter, multiply by 0.01; hemoglobin to grams per liter, multiply by 10.0; lactate to millimoles per liter, multiply by 0.111; platelet count to ×10^9^/L, multiply by 1.0.

^a^ Race and ethnicity were reported by the patient and/or substitute decision-maker.

^b^ ISS, an overall assessment of body injury, is calculated as the sum of squares of the 3 highest ISSs for body parts. Total score ranges from 0 to 75, with higher scores indicating greater injury. A score greater than 15 indicates major trauma.

^c^ Head AIS assesses head injury on a scale of 0 to 6, with 0 indicating no injury and 6 indicating unsurvivable injury.

^d^ GCS, a measure of level of consciousness, is based on eye, verbal, and motor responses. Total score ranges from 3 to 15, with higher scores indicating greater disability.

^e^ ABC uses pulse rate, SBP, abdominal sonography, and mechanism of injury to determine need for massive transfusion. Scores range from 0 to 4, with scores of 2 to 4 indicating higher likelihood of requiring massive transfusion.

^f^ SI is calculated from a simple equation relating HR and SBP (SI = HR / SBP in mm Hg). SI higher than 1.0 indicates the development of hypotension and the need for massive transfusion with higher sensitivity, compared with HR and SBP in isolation.

^g^ Laboratory reference ranges are hemoglobin: 130 to 180 mg/dL; lactate: less than 1.7 mmol/L; platelets: 150 to 450 ×10^9^/L; fibrinogen: 1.7 to 4 mg/dL; INR: 0.8 to 1.2.

^h^ INR, the ratio between the prothrombin time of the patient and the prothrombin time reference value of the laboratory. INR higher than 1.2 indicates posttraumatic coagulopathy; INR higher than 1.5 indicates severe posttraumatic coagulopathy.

Coagulopathy (International Normalized Ratio [INR] > 1.2) was identified in 36 patients (54.5%) in the FC-PCC group and in 37 patients (52.1%) in the control group. The critical administration threshold (transfusion of ≥3 RBC units within the first hour of admission) was positive in most patients (FC-PCC: 52 [78.8%]; FP: 60 [84.5%]). Tranexamic acid was administered to 123 patients (89.8%) (Table 1). Median (IQR) time from injury to hospital admission was similar in both the FC-PCC group (45 [36-55] minutes) and FP control group (52 [34-66] minutes).

**Figure 2.**
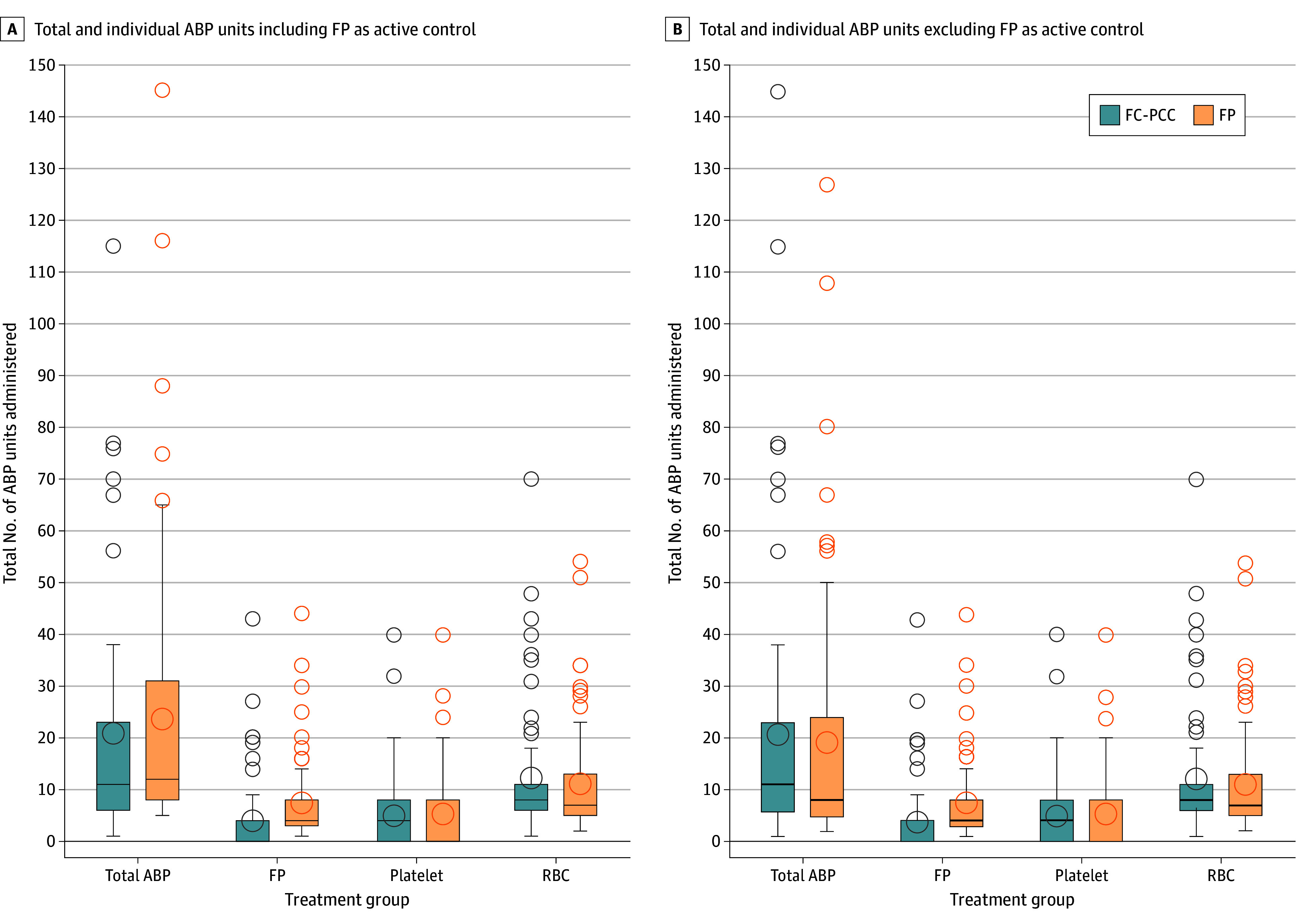
Allogeneic Blood Products (ABPs) Administered in the First 24 h After Admission to the Trauma Bay Error bars represent 95% CIs. FC-PCC indicates fibrinogen concentrate and prothrombin complex concentrate; FP, frozen plasma; RBC, red blood cell.

No significant difference was observed in the primary outcome. The mean 24-hour ABP transfusions were 20.8 (95% CI, 16.7-25.9) units in the FC-PCC group and 23.8 (95% CI, 19.2-29.4) units in the FP group, and the LSM ratio was 0.87 (97.5% CI, 0.00-1.19; *P* = .20 for superiority) (Table 2, Figure 2). Similarly, there were no significant differences between mean ABP transfusions administered within 24 hours without FP as the active control. The mean 24-hour ABP transfusions were 20.8 (95% CI, 16.4 to 26.3) units in the FC-PCC group and 19.1 (95% CI, 15.2 to 24.0) in the FP group, and the LSM ratio was 1.09 (95% CI, 0.00-1.51; *P* = .69). In addition, no significant differences were identified in the the individual number of each ABP transfusion administered within 24 hours (Table 2; eFigure 1 in Supplement 3).

**Table 2.** Trial Outcomes by Treatment Group

Outcome	FP group (n = 71)	FC-PCC group (n = 66)	*P* value
Primary			
ABPs transfused within 24 h, mean (95% CI), units	23.8 (19.2-29.4)	20.8 (16.7-25.9)	.20^a^
Mean (SD)	23.8 (25.9)	20.8 (26.3)	
Median (IQR)	12.0 (8-31)	11.0 (6-23)	
Secondary			
ABPs transfused within 24 h without FP as active control, mean (95% CI), units	19.1 (15.2-24.0)	20.8 (16.4-26.3)	.69^a^
Mean (SD)	19.1 (24.2)	20.8 (26.3)	NA
Median (IQR)	8.0 (5-24)	11.0 (6-23)	NA
RBC within 24 h, median (IQR), units	7 (5-13)	8.0 (6-11)	NA
Plasma within 24 h, median (IQR), units	4 (3-8)	0 (0-4)	NA
Platelets within 24 h, median (IQR), units	0 (0-8)	4 (0-8)	NA
Plasma within 24 h without control, median (IQR), units	0 (0-4)	0 (0-4)	NA
Rescue of FC within 24 h, No. (%)	42 (59.1)	17 (25.8)	<.001
Median (IQR), g	4 (0-6)	0 (0-2)	NA
Rescue of PCC, No. (%)	2 (2.8)	3 (4.5)	.59
Median (IQR),g	0 (0-4000)	0 (0-2000)	NA
Days out of hospital at 28 d, median (IQR)	0 (0-7)	0 (0-11)	.19
ICU-free days at 28 d, median (IQR)	10 (0-19)	11 (0-20)	.40
Ventilator-free days at 28 d, median (IQR)	16 (0-23)	16 (0-24)	.62
Disposition at 28 d, No. (%)			
Hospitalized	32 (45.1)	26 (39.4)	NA
Home	13 (18.3)	15 (22.7)	NA
Died	15 (21.1)	9 (16.6)	NA
Rehabilitation	6 (8.4)	6 (9.1)	NA
Other acute care facility	5 (7.0)	10 (15.1)	NA
Abdominal compartment syndrome, No. (%)	1 (1.4)	0	NA
Limb compartment syndrome, No. (%)	1 (1.4)	5 (7.6)	NA
24-h mortality, No. (%)	12 (16.9)	5 (7.6)	.24^b^
All-cause mortality at 28 d, No. (%)	15 (21.1)	9 (13.6)	.26^c^
Any TEAE, No. (%), [95% exact CI]^d^	10 (14.1) [6.9-24.4]	14 (21.2) [12.1-33.0]	.37^e^

Abbreviations: ABP, allogeneic blood product; FC, fibrinogen concentrate; FP, frozen plasma; ICU, intensive care unit; LSM, least-squares means; NA, not applicable; PCC, prothrombin complex concentrate; RBC, red blood cell; TEAE, treatment-emergent adverse event.

^a^ LSM ratio (FC + PCC/FP) with 1-sided 97.5% CI.

^b^ Log-rank test for comparison between groups.

^c^ Cox proportional hazards regression model used for comparison between time to death within 28 days.

^d^ Exact 95% CI is based on exact binomial distribution (Clopper-Pearson).

^e^ Fisher exact test *P* value.

The need for rescue with FC in the FC-PCC group was lower compared with the FP group (17 [25.8%] vs 42 [59.1%]; odds ratio [OR], 0.24 [95% CI, 0.12-0.50]; *P* < .001). In addition, patients in the FP group were rescued with a higher median (IQR) dose of FC within the first 24 hours compared with patients in the FC-PCC group (4 [0-6] g vs 0 [0-2] g) (Table 2). No significant differences were identified for rescue with PCC in both FC-PCC group and FP group (3 [4.5] g vs 2 [2.8] g, respectively; OR, 1.64 [95% CI, 0.27-10.10]; *P* = .59]). The coagulation profiles in both groups across the first 24 hours are shown in eFigure 2 in Supplement 3.

The median (IQR) doses of FC and PCC, administered in pack 1 and pack 2, were 4 (4-4) g and 2000 (2000-2000) IU, respectively. The median (IQR) dose of FP was 4 (2-4) units in pack 1 and 4 (4-4) units in pack 2. A higher compliance with administration of full doses of FC-PCC compared with FP, in both first and second packs was observed (eTable 2 in Supplement 3).

**Figure 3.**
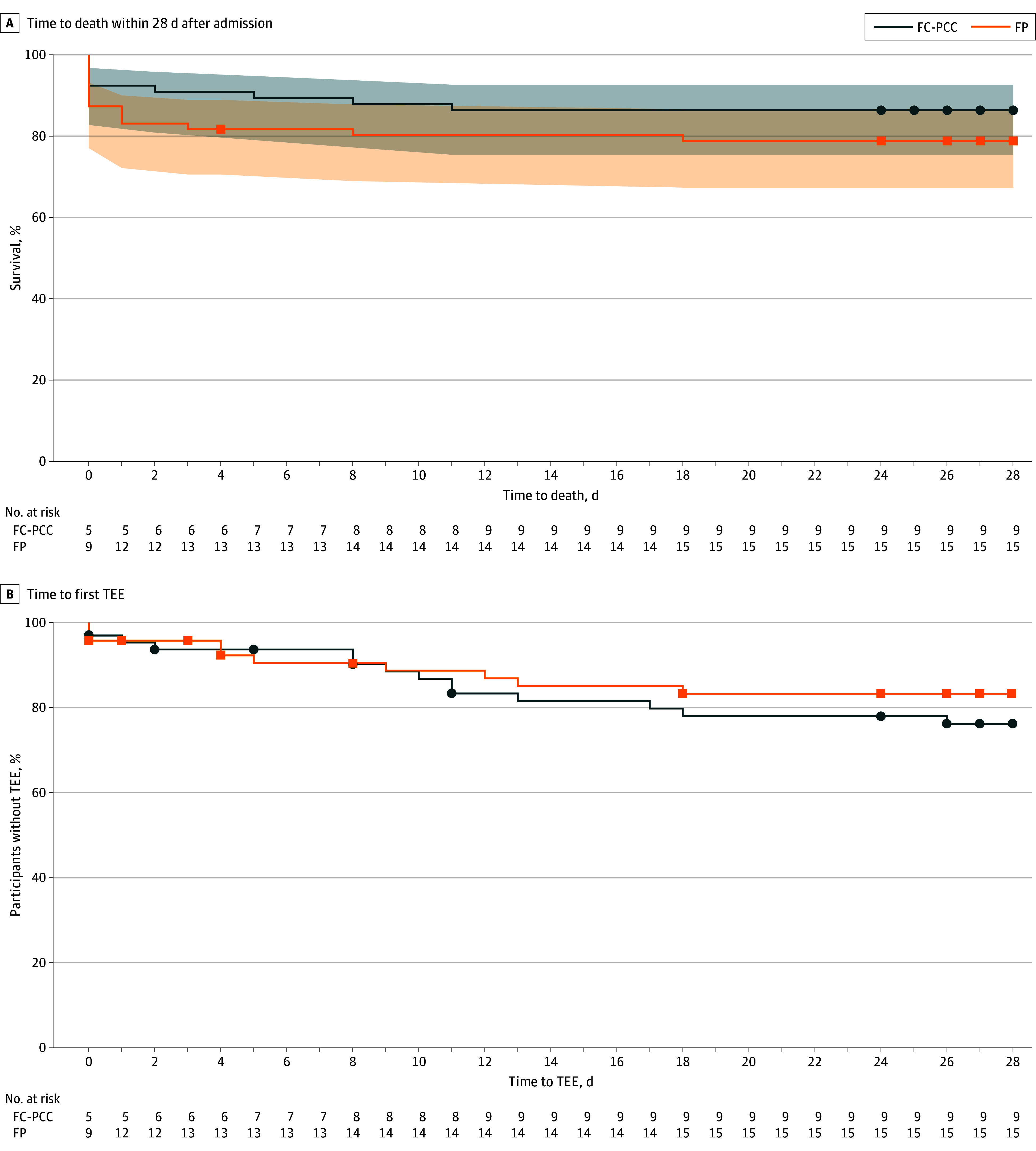
Time to Death Within 28 d After Admission and Time to First Thromboembolic Event (TEE) Squares represent censored time (last visit date or death date; limited to 28 days). FC-PCC indicates fibrinogen concentrate and prothrombin complex concentrate; FP, frozen plasma.

No statistical significance in 24-hour mortality was observed between the FC-PCC and FP groups (5 [7.6%] vs 12 [16.9%]; *P* = .24) (Table 2). In addition, there were no differences in time to death and risk distribution between groups for all-cause 28-day mortality (hazard ratio, 0.62 [95% CI, 0.26-1.40, *P* = .26] and 0.64 [95% CI, 0.30-1.37, *P* = .25], respectively) (Table 2 and Figure 3).

The number of patients with at least 1 TEE was 14 (21.2%) in the FC-PCC group and 10 (14.1%) in the FP group (risk difference, 7.13; 95% CI, −5.89 to 20.65; *P* = .37) (Table 2). While this difference was not statistically significant, the interpretation of TEE rates should consider the numerically higher survival in the FC-PCC group (deaths: 9 [13.6%] in FC-PCC group vs 15 [21.1%] in FP group), which resulted in a longer period at risk for TEEs. All additional deaths in the FP group occurred within the first 24 hours, prior to the typical time frame for thromboembolic complications, potentially affecting the exposure time for TEEs between the treatment groups. An exposure-adjusted incidence rate analysis was conducted and had findings consistent with the main results (Table 2 and Figure 3).

No between-group difference was observed in other secondary efficacy and safety clinical outcomes (Table 2). The primary outcome in subgroups of interest is available in eTable 3 in Supplement 3. Primary and secondary outcomes in the per-protocol population were similar to the mITT population and are provided in eTable 4 in Supplement 3.

## Discussion

In the FiiRST-2 trial, the efficacy and safety of preemptive fixed-dose factor concentrates administered preemptively in the initial resuscitation of severely injured patients with trauma compared with FP as the standard of care showed no significant reduction in 24-hour ABP units. Similarly, no significant difference in secondary outcomes, including thromboembolic complications, was identified. This RCT was terminated early because the conditional power was below 25% at the a priori interim analysis.

The rationale for conducting the FiiRST-2 trial was based on 2 observational studies showing reduced RBC transfusions and no difference in TEEs with factor concentrates plus plasma vs plasma alone^16,17^ as well as the RETIC trial comparing FC-PCC vs plasma and finding higher massive transfusion rates with need for hemostatic rescue therapy in patients receiving plasma.^9^ The preemptive use of FC and PCC in the FiiRST-2 trial was chosen for their logistical advantages, potentially safer profile than FP, and adoption in remote centers due to no plasma access.^3,6^ The FC-PCC dose adopted was based on clinical practice guidelines,^3^ the observational data,^18,19^ and the RETIC trial.^9^

While the FiiRST-2 trial was ongoing, the CRYOSTAT-2^7^ and PROCOAG^8^ RCTs were published. CRYOSTAT-2 assessed plasma with empiric fibrinogen replacement vs standard of care (plasma with delayed fibrinogen replacement) and found no differences in mortality or thromboembolic complications.^7^ PROCOAG evaluated 4-factor (4F) PCC preemptively vs placebo along with a ratio-based plasma strategy and identified no difference in transfusion requirements or mortality. However, the trial identified an increased risk of thromboembolic complications in patients who received PCC and plasma, in an unadjusted analysis with a small numerical difference in survival between groups.^8^

The FiiRST-2 and PROCOAG trials had similar 24-hour ABP units transfused. Overall, patients in both trials had similar coagulation profiles (ie, median INR, fibrinogen level, and platelet count), marker of tissue perfusion (lactate), Assessment of Blood Consumption score of 2 or higher, head Abbreviated Injury Scale higher than 2, and similar requirements for hemostasis control procedures. The differences between the 2 trials included lower median (IQR) admission SBP in PROCOAG (4F-PCC: 89 [70-115] mmHg; control group: 90 [74-110] mmHg) compared with FiiRST-2 (FC-PCC: 108 [93-138] mmHg; control group: 127 [85-152] mmHg); higher rates of coagulopathy (INR >1.2: INR >1.2: 93 [65%] in the 4F-PCC group and 89 [68%] in the control group compared with FiiRST-2 (36 [54.5%] in the FC-PCC group; 37 [52.1%] in the control group). In addition, the median (IQR) the ISS was higher in PROCOAG than in FiiRST participants (median [IQR] 34 [25-50] in the 4F-PCC group vs 38 [29-50] in the control group compared with 33 [18-45] in the FC-PCC group and 29 [22-43] in the control group. FiiRST-2 participants had higher rates of tranexamic acid administration. The longer median (IQR) arrival time in the PROCOAG vs FiiRST-2 trial (105 [80-132] minutes in the 4F-PCC group and 100 [75-132] minutes in the control group compared with FiiRST-2 45 [36-55] minutes in the FC-PCC group and 52 [34-66] minutes in the control group) may account for the hemodynamic and coagulation differences observed. Participants in the FiiRST-2 trial received FC-PCC or FP 43 and 37 minutes earlier following injury, respectively, likely due to longer prehospital times in the PROCOAG trial. Despite higher hypotension, ISS, and coagulopathy, PROCOAG patients had lower rates of 3 or more RBC units transfused within the first hour following admission, a critical administration threshold, compared with FiiRST-2 participants (67 [42%] and 60 [38%] vs 52 [78.8%] and 60 [84.5%) in intervention and control groups, respectively).^20,21^ Differences in prehospital emergency medical services between France^22,23^ and Canada^24,25^ may explain why PROCOAG patients had lower transfusion rates after admission (ie, likely due to prehospital transfusions). However, the study did not report or analyze the number of prehospital units transfused.

In the FiiRST-2 trial, no significant differences in thromboembolic complication were found between groups. Due to the higher number of FC-PCC 28-day survivors (deaths: 9 [13.6%] in FC-PCC group vs 15 [21.1%] in FP group), we adjusted the incidence risk for exposure.^26^ In the PROCOAG trial, a marginally higher incidence of TEEs in patients who received PCC was reported, but no adjustments were made for exposure (deaths: 20 in PCC group vs 26 in placebo group).^8^

### Future Directions

Future trials assessing factor concentrates in trauma should focus on optimizing patient selection. This optimization likely can be achieved by applying novel data-driven methods that leverage machine learning algorithms and continuous vital signs data to improve early prediction of TIC and the need for MHP activation.^27,28,29,30^ In addition, RCTs should use 24-hour mortality as the primary outcome^31,32^ based on the numerically higher number of survivors in both PROCOAG and FiiRST-2 trials. Moreover, future trials should involve thromboembolism experts in the design phase and plan closer follow-up on TEEs, ensuring compliance with evidence-based guidelines.^33^ Furthermore, analyses of TEEs should include adjustment for time exposure, with adjusted rates provided to the IDSMC.

### Strengths and Limitations

The strengths of this RCT include its design, which ensured that both FC-PCC and FP were administered only to patients who were markedly bleeding. Clinicians were instructed to cut the tamper seal on the MHP pack only if they deemed it necessary to transfuse factor concentrates, which minimized unnecessary administration to patients with prompt bleeding control. Second, when clinicians opted to administer FC-PCC or FP, full doses were administered to most patients. Third, due to shorter transport times in this trial, the intervention was administered much faster than in the PROCOAG^8^ and CRYOSTAT-2^7^ trials. Fourth, due to the pragmatic trial design with screening of consecutive patients, the results are likely generalizable to the broad trauma population.

The FiiRST-2 study has several limitations. First, to be pragmatic, we used MHP activation as a starting point to screen and randomly assign patients. However, variability in activation criteria across centers and difficulty in determining the need for massive transfusion^34,35,36^ led to more overactivation than observed in the pilot study.^14^ Second, we included patients with cardiopulmonary resuscitation in progress or catastrophic injuries with low survival probability and who required large amounts of ABPs, contributing to the low conditional power detected in the interim analysis. Third, a more personalized strategy (ie, viscoelastic testing) was not included due to the inconsistent use and lack of availability at participating sites. We also aimed to assess FC-PCC use preemptively due to the geographic challenges and limited access to plasma in small, remote centers.^3,6^ Fourth, we identified a higher compliance with FC-PCC, likely due to its smaller volume, allowing faster infusion compared with FP. This difference may have resulted in some patients dying before the complete FP dose was administered or led some clinicians to discontinue FP for patients who had bleeding control. Fifth, there is opportunity to decrease the time of FC-PCC administration by storing products in the emergency department and assigning a team member to prepare them, thus avoiding delays in administration.

## Conclusions

The FiiRST-2 RCT found no reduction in blood product transfusion within 24 hours for severely injured patients with trauma treated preemptively with FC-PCC vs FP. There was no difference in secondary efficacy and safety outcomes, including TEE, between treatment groups.

## Notes

Supplement 1.
Study Protocol

Supplement 2.
Statistical Analysis Plan

Supplement 3. **eTable 1.** Massive Hemorrhage Protocol Activation Criteria at Each Participating Site **eTable 2.** Details on Compliance With the Administration of Intervention and Control **eTable 3.** Primary Outcome in Subgroups of Interest **eTable 4.** Trial Outcomes by Treatment Group in the Per Protocol Population **eFigure 1.** Transfusion of Blood Products Across the First 24 Hours Following Admission, mITT N=137 **eFigure 2.** Coagulation Profile and Hemoglobin Across the First 24 Hours Following Admission, mITT N=137

Supplement 4.
Data Sharing Statement
