# Error in the Byline

**DOI:** 10.1001/jamanetworkopen.2025.53844

**Published:** 2025-12-19

In the Original Investigation titled “Factors in the Initial Resuscitation of Patients With Severe Trauma: The FiiRST-2 Randomized Clinical Trial”^1^ published on September 22, 2025, an author’s name was omitted from the byline. Dr Solh’s name and academic degrees were added to the byline as follows: Ziad Solh, MD, MSc. Dr Solh is affiliated with the Division of Transfusion Medicine, Department of Pathology and Laboratory Medicine, Schulich School of Medicine and Dentistry, Western University, London, Ontario, Canada and Division of Hematology, Department of Medicine, Schulich School of Medicine and Dentistry, Western University, London, Ontario, Canada. This article has been corrected.^1^
