# Supplementary material for: Factors in the Initial Resuscitation of Patients With Severe Trauma: The FiiRST-2 Randomized Clinical Trial
Source: JAMA Netw Open. 2025 Sep 22;8(9):e2532702. doi: 10.1001/jamanetworkopen.2025.32702 (PMC12455389; doi:10.1001/jamanetworkopen.2025.32702)
Supplement: Supplement 1. — Study Protocol [file jamanetwopen-e2532702-s001.pdf]

CLINICAL STUDY PROTOCOL

FiiRST-2

Factors In the Initial Resuscitation of Severe Trauma 2

Prospective, multi-center, randomized, parallel-control, superiority study comparing administration of clotting factor concentrates with a standard massive hemorrhage protocol in severely bleeding trauma patients.

**Investigational Products:**	*Fibryga* and *Octaplex*
**Indication:**	Acute trauma-associated coagulopathy
**Study Design:**	Multicenter, randomized, controlled, superiority trial, utilizing a conventional, parallel group, two-armed, with an adaptive two-stage design.
**Sponsor:**	**Keyvan Karkouti, MD**<br>Department of Anesthesia<br>Toronto General Hospital<br>200 Elizabeth Street, 3EN<br>Toronto, ON<br>M5G 2C4<br>Canada
**Abbreviated Study Name:**	FiiRST-2
**Clinical Registration Number:**	NCT04534751
**Clinical Trials Ontario Project ID:**	2031
**Development Phase:**	Phase 4
**Planned Clinical Start:**	11-Jan-2021
**Planned Clinical End:**	31-Dec-2023
**Date of Protocol:**	11-June-2022
**Version:**	3.0
**Principal Investigator:**<br>**Co-Investigator:**	Luis Teodoro da Luz, MD<br>Jeannie Callum, MD

STUDY OUTLINE

**Name of Sponsor:**<br>Keyvan Karkouti, Toronto General Hospital	
**Name of Investigational Products:**<br>*Fibryga* and *Octaplex*	**Protocol Identification Code:**<br>FiiRST-2
**Name of Active Ingredients:**<br>Human fibrinogen concentrate and 4-factor prothrombin complex concentrate	**Date of Final Protocol:**<br>01-Dec-2020

Title of Study:

Prospective, multi-center, randomized, parallel-control, superiority study comparing administration of clotting factor concentrates with a standard massive hemorrhage protocol in severely bleeding trauma patients

Indication:

Severely bleeding trauma patients

Number of Study Centers:

Eleven Canadian hospitals:

- Sunnybrook Health Sciences Centre, Toronto
- Saint Michael's Hospital, Toronto
- Montreal General Hospital, Montreal
- Foothills Medical Centre, Calgary
- Vancouver General Hospital, Vancouver
- The Ottawa Hospital, Ottawa
- Hamilton General Hospital, Hamilton
- London Health Sciences Centre, London
- Kingston Health Sciences Centre, Kingston
- Royal Alexandria Hospital, Edmonton
- University of Alberta Hospital, Edmonton

Objectives:

Primary Objective:

The primary objective of this study is to determine the impact of early co-administration of fibrinogen concentrate (FC) and prothrombin complex concentrate (PCC) on the total number of allogeneic blood products (ABPs) transfused compared to the current standard of care (frozen plasma [FP] administered in a ratio-based plasma resuscitation).

Name of Sponsor:<br>Keyvan Karkouti, Toronto General Hospital	
**Name of Investigational Products:**<br>*Fibryga* and *Octaplex*	**Protocol Identification Code:**<br>FiiRST-2
**Name of Active Ingredients:**<br>Human fibrinogen concentrate and 4-factor prothrombin complex concentrate	**Date of Final Protocol:**<br>01-Dec-2020

Secondary Objectives:

- To compare the hemostatic efficacy of the intervention with the standard of care, as measured by transfusion of ABPs, use of hemostatic interventions, and correction of coagulopathy based on laboratory tests
- To assess the safety of the intervention, focusing on arterial and venous thromboembolic complications

Study Design:

FiiRST-2 is a multicenter, randomized, parallel-control, superiority trial, utilizing a conventional two-armed, two-stage design, with an adaptive interim analysis, performed at Level 1 Trauma Centers in Canada. The study is designed to examine the effect of replacing fibrinogen and clotting factors via FC and PCC following activation of the massive hemorrhage protocol (MHP) on the number of ABP units transfused in trauma patients with severe hemorrhage versus the current standard of care (ratio-based plasma resuscitation).

Number of patients

The study will enroll up to 350 trauma patients with approximately 175 assigned to each of the two treatment groups. Due to the inherent variability in the primary endpoint and a yet substantial uncertainty about the effect size, an adaptive design approach will be used. For this, a planned interim analysis will be performed after 120 patients have completed the study. Primary aim of this interim analysis is to calculate the p-value and conditional power of the test statistic and perform a sample size re-assessment. This will be done in an unblinded interim analysis performed by an independent statistician who will report the results only to the independent data safety monitoring committee (IDSMC) which will make recommendations to the sponsor without revealing the treatment groups. Hence, the final number of enrolled patients will depend on the sample size re-calculation.

Patient Selection Criteria:

Inclusion Criteria:

Severely injured adult trauma patients who meet all following criteria:

1. Estimated age greater than 16 years old
2. Severely injured (penetrating or blunt) trauma patients
3. Triggered MHP within first hour of hospital arrival at the trauma bay/ED

**Name of Sponsor:**<br>Keyvan Karkouti, Toronto General Hospital	
**Name of Investigational Products:**<br>*Fibryga* and *Octaplex*	**Protocol Identification Code:**<br>FiiRST-2
**Name of Active Ingredients:**<br>Human fibrinogen concentrate and 4-factor prothrombin complex concentrate	**Date of Final Protocol:**<br>01-Dec-2020

Exclusion Criteria:

Patients who meet any of the following criteria are *not* eligible for the study:

1. Have received more than 2 U RBCs during the pre-hospital phase of care
2. Have received more than 2 U RBCs in the trauma bay/ED before activation of the MHP
3. Have an elapsed time from injury of more than 3 hours
4. Have a penetrating traumatic brain injury with Glasgow Coma Scale (GCS) of 3
5. Are suspected or known to be on anticoagulants in the last 7 days
6. Have known congenital or acquired bleeding disorders
7. Have a known pregnancy
8. Refuse blood transfusion due to religion or other reasons
9. Previous history of heparin induced thrombocytopenia (HIT)

Test Products, Dose, and Mode of Administration:

Patients will be randomized if the MHP is activated according to the MHP activation criteria at each study site. Once eligibility is confirmed, the blood bank medical laboratory technologist will randomize the patient to one of two groups: the intervention group or the control group.

Intervention group: 4 g *Fibryga* and 2000 IU *Octaplex* will be released as part of the first and second MHP packs.

Control group: 4 U FP will be released as part of the first and second MHP packs.

Concomitant therapy: In both groups, 4 U RBC will be included as part of the first and second MHP packs, and 1 dose of platelets (4 U of pooled random donor or single donor apheresis) will also be included as part of the second MHP pack. Both RBCs and platelets will be administered according to the clinical situation and/or lab results as per the discretion of the clinical team. The second MHP pack will be released at the request of the clinical team, but clinicians will be instructed to administer all of the investigational product (*Fibryga/Octaplex* or FP) in the first pack before moving onto the second pack. Similarly, if the second pack is opened, clinicians will be instructed to administer all of the investigational products contained within, before moving to the third pack. Not administering all of the investigational products in the first pack, once started, will be a protocol deviation.

Administration of all non-investigational products will be at the discretion of the clinical team according to the hemodynamic status of the patient and/or laboratory results (standard and/or point-of-care as per institutional practice). While platelets will be routinely included in the second pack, clinicians can request platelets outside of the packs (e.g., for patients on antiplatelet therapy or with marked thrombocytopenia). In the control group, FC may be administered if hypofibrinogenemia (fibrinogen level below 1.5–2.0 g/L or FIBTEM A10 below 8–12 mm) is identified as part of routine testing, at the discretion of the clinical team.

**Name of Sponsor:**<br>Keyvan Karkouti, Toronto General Hospital	
**Name of Investigational Products:**<br>*Fibryga* and *Octaplex*	**Protocol Identification Code:**<br>FiiRST-2
**Name of Active Ingredients:**<br>Human fibrinogen concentrate and 4-factor prothrombin complex concentrate	**Date of Final Protocol:**<br>01-Dec-2020

Patients in the intervention group can receive additional FC if hypofibrinogenemia (as per above criteria) is identified after the full dose (4g) in the first pack is administered (if the second pack is not opened). If the second pack is opened, additional doses of FC will be permitted after the full dose (4g) in the second pack is administered.

Patients in the control group will not be permitted to receive PCC during the study. Patients in the intervention group may receive FP in the third and subsequent MHP packs. If a third MHP pack is required, and thereafter, MHP packs will contain ABPs (RBCs, platelets and plasma) according to guidelines at each participating site or revert to laboratory-guided transfusion as per the local guidelines once bleeding is controlled. The MHP should be terminated once bleeding is controlled and the MHP criteria are no longer met. Termination of the MHP may occur at any time based on the discretion of the clinical team.

The maximum time frame for administration of the second MHP pack (if required) for both groups is 24 hours from arrival to the trauma bay/ED or termination of the MHP (whichever comes first).

Duration of Treatment:

The duration of treatment is from randomization following activation of the MHP until all units within the second MHP pack have been administered or until MHP is terminated, whichever occurs first. The maximum time frame for initiation of administration of the second MHP pack is 24 hours from arrival at the trauma bay/ED. Should administration of units from the second pack occur prior to 24 hours post trauma bay/ED arrival and the infusion extend beyond 24 hours, all ABP units transfused from the pack will be included in the primary endpoint analysis.

Study Outcome Parameters (Primary and Secondary Endpoints):

Primary Endpoint:

The primary endpoint is to demonstrate superiority with respect to the composite number of all ABP units (RBCs, FP and platelets) transfused within 24 hours following arrival at the trauma bay/ED.

Secondary endpoints:

- Total number of units of RBCs transfused within the first 24 hours following arrival at the trauma bay/ED
- Incidence of thromboembolic events, as defined by evidence of any of the following, from arrival at the trauma bay/ED, up to 28 days:
  - Deep vein thrombosis (DVT)
  - Pulmonary embolism (PE)
  - Myocardial infarction (MI) [1]
  - Ischemic stroke [2]

**Name of Sponsor:**<br>Keyvan Karkouti, Toronto General Hospital	
**Name of Investigational Products:**<br>*Fibryga* and *Octaplex*	**Protocol Identification Code:**<br>FiiRST-2
**Name of Active Ingredients:**<br>Human fibrinogen concentrate and 4-factor prothrombin complex concentrate	**Date of Final Protocol:**<br>01-Dec-2020

- Arterial or venous thrombosis at other sites
- Ventilator-free days, defined as the number of days up to Day 28 following arrival at the trauma bay/ED on which a patient breathed without assistance (if period of unassisted breathing lasted at least 48 consecutive hours). Patients who die during study follow-up or require 28 or more days of mechanical ventilation will be assigned zero ventilator-free days [3]

Additional Endpoints:

Additional efficacy endpoints:

- Total and individual numbers of units and volumes (liters) of ABPs (RBCs, FP and platelets) transfused within 6 hours, 24 hours and within 7 days post arrival at the trauma bay/ED
- Total volume of crystalloids and other colloids administered within the first 6 and 24 hours following arrival at the trauma bay/ED
- Rescue use of hemostatic agents (fibrinogen concentrate and rFVIIa) within the first 24 hours following arrival at the trauma bay/ED
- Laboratory endpoints upon arrival (before drug administration), if measured, and following infusion of the investigational medicinal products (IMPs), as per each site protocol routine, measured within the first 24 hours and within 7 days following arrival at the trauma bay/ED:
  - Plasma fibrinogen levels
  - International normalized ratio (INR), prothrombin time (PT) and activated partial thromboplastin time (aPTT)
  - Hemoglobin and hematocrit levels
  - Platelet count
  - Base deficit, pH and lactate
  - Thromboelastometry values: EXTEM clotting time (CT), EXTEM A10, EXTEM maximum clot firmness (MCF), FIBTEM A10, FIBTEM MCF, and EXTEM LY30
- Days out of hospital within the first 28 days following arrival at the trauma bay/ED
- Time to death over the first 28 days following arrival at the trauma bay/ED

Additional safety endpoints:

- All documented adverse events (AEs) and serious adverse events (SAEs) during the first 28 days following arrival, including:
  - Multi organ failure (MOF) as measured by the Sequential Organ Failure Assessment (SOFA) score [4] daily during ICU stay for up to 28 days following arrival at the trauma bay/ED
    - Highest SOFA score and time of highest SOFA score
    - Change in SOFA score

**Name of Sponsor:**<br>Keyvan Karkouti, Toronto General Hospital	
**Name of Investigational Products:**<br>*Fibryga* and *Octaplex*	**Protocol Identification Code:**<br>FiiRST-2
**Name of Active Ingredients:**<br>Human fibrinogen concentrate and 4-factor prothrombin complex concentrate	**Date of Final Protocol:**<br>01-Dec-2020

- Incidence of ACS, defined as sustained intra-abdominal pressure >20 mmHg with or without an abdominal perfusion pressure (APP) of <60 mmHg, that is associated with new organ dysfunction/failure [5]
- Incidence of LCS [6]
- Incidence of transfusion reactions as defined by the International Society of Blood Transfusion [7]
- Incidence of treatment-emergent adverse events (TEAEs)

- Duration of ICU stay
- 28-day all-cause mortality

Study Procedures:

Upon arrival at the trauma bay/ED and activation of the MHP, the blood bank technologist will confirm the following criteria:

- The approximate time of injury and the arrival time at the trauma bay/ED
- The patient's age
- The inclusion and exclusion criteria are met

If the patient meets the inclusion criteria and has no exclusion criteria, the blood bank technologist will then randomize patients according to the randomization schedule and prepare and release the products.

Visit 1: Upon arrival at the trauma bay/ED

- Record date and time of arrival at the trauma bay/ED
- Collect injury data

Visit 2: 24 hours following arrival at the trauma bay/ED

- Collect demographic characteristics, medical history and concomitant medications, record date and time of arrival at the trauma bay/ED, and injury data
- Obtain deferred consent by substitute decision maker (SDM) or patient if recovered
- Collect total and individual numbers of units and volumes of ABPs (RBCs, FP and platelets) transfused within 6 hours and 24 hours post arrival at the trauma bay/ED
- Record crystalloid and colloid requirements within the first 6 hours and 24 hours following arrival at the trauma bay/ED
- Record rFVIIa and FC use 24 hours following arrival at the trauma bay/ED
- Collect laboratory assessments 24 hours following arrival at the trauma bay/ED, where available
    - Collect viscoelastic tests measurements, where available
- Record thromboembolic events, including leg Doppler ultrasound or other imaging, as per clinical indications

**Name of Sponsor:**<br>Keyvan Karkouti, Toronto General Hospital	
**Name of Investigational Products:**<br>*Fibryga* and *Octaplex*	**Protocol Identification Code:**<br>FiiRST-2
**Name of Active Ingredients:**<br>Human fibrinogen concentrate and 4-factor prothrombin complex concentrate	**Date of Final Protocol:**<br>01-Dec-2020

- Record all AEs, SAEs, including MOF (SOFA score), ACS and LCS, transfusion reactions, and TEAEs

Visit 3: Days 2–27 following arrival at the trauma bay/ED

- Obtain deferred consent by SDM or patient if recovered
- Collect total and individual numbers of units and volumes of ABPs (RBCs + FP + platelets) transfused (Day 7)
- Collect laboratory assessments, where performed (Day 7)
  - Collect point-of-care coagulation measurements, where available
- Record thromboembolic events, including leg Doppler ultrasound or other imaging, as per clinical indications
- Record all AEs and SAEs, including MOF (SOFA score, collected daily for patients still in ICU), ACS and LCS, transfusion reactions, and TEAEs

Visit 4: Day 28 (in person if in hospital or by phone)

- Obtain deferred consent by SDM or patient if recovered
- Record thromboembolic events, including leg Doppler ultrasound or other imaging as per clinical indications
- Record ventilator-free days
- Record days out of hospital within the 28 days of follow-up
- Record time to death
- Record all AEs and SAEs, including MOF (SOFA score, for patients still in ICU), ACS and LCS, transfusion reactions, and TEAEs
- Record duration of ICU stay
- Record 28-day all-cause mortality

Statistical Analysis Plan:

To demonstrate that the early administration of FC and PCC is clinically superior to the standard of care (FP administered in a balanced ratio), with respect to the mean number of ABP units administered within 24 hours of arrival at the trauma bay/ED, a two-sample, one-sided test of the pair of hypotheses: H_0: RR $\geq$ RR_0 vs. Ha: RR < RR_0 will be carried out with a type I error probability of $\alpha = 0.025$. Here, λ_1 and λ_2 denote the mean number of ABPs (RBCs + FP + platelets) in the control group (standard of care) and intervention group, respectively, RR is the ratio $\lambda_2/\ \lambda_1$, and RR_0 will be set equal to 1.0 to test for superiority. A mean difference in 5 U of the composite outcome (mean 15 U in the control group and mean 10 U in the intervention group) is considered as a clinically meaningful difference that should be detected with at least 80% power.

**Name of Sponsor:**<br>Keyvan Karkouti, Toronto General Hospital	
**Name of Investigational Products:**<br>*Fibryga* and *Octaplex*	**Protocol Identification Code:**<br>FiiRST-2
**Name of Active Ingredients:**<br>Human fibrinogen concentrate and 4-factor prothrombin complex concentrate	**Date of Final Protocol:**<br>01-Dec-2020

Summary data for continuous variables will be presented as means and standard deviations, or medians and interquartile ranges, depending on the distribution. Discrete variables will be summarized as frequency and percentages. All-cause mortality will be analyzed by the intention-to-treat (ITT) and per-protocol (PP) populations. Differences in binary outcomes will be assessed using exact tests for proportions. Likelihood ratio test in the context of a generalized linear model (GLM) for count data will be used to analyze ratios of blood and blood products. For categorical clinical endpoints, relative risks (RR) and 95% confidence interval (CI) will be calculated. ARDS-free survival will be used to account for deaths and lost-to-follow-up and will be analyzed using log-rank tests.

Adaptive design: Due to the inherent variability in the primary endpoint and a yet substantial uncertainty about the effect size, an adaptive design approach will be used. For this, a single interim analysis will be performed after 120 patients have completed the study. Primary aim of this interim analysis is to calculate the p-value and conditional power of the test statistic for the primary endpoint and perform a sample size re-assessment. This will be an unblinded interim analysis performed by an independent statistician who will report the results only to the independent data safety monitoring committee (IDSMC) which will make recommendations to the sponsor without revealing the treatment groups. The study design will follow a group sequential design with O'Brien-Fleming error-spending function, a futility boundary and sample size re-estimation based on conditional power. Hence, the recommendation of the IDSMC can include:

- To continue the trial as planned until 350 patients have completed the study,
- To stop the trial for demonstrated superiority at the interim analysis,
- To stop the trial at the interim for futility or for requiring an increase in sample size that is considered unfeasible
- To continue the trial with a modified sample size

The primary analysis will be performed on the modified intention-to-treat (mITT) population. A secondary analysis will be performed for the per-protocol (PP) population.

The safety analysis population (SAF) will include all randomized patients who receive any of the interventional products in the first MHP pack or beyond the intended first-line treatment and agree to remain in the study after consenting.

FLOW CHART OF ASSESSMENTS

Table 1 Flow Chart of Assessments Performed Throughout the Study

**Procedures**	**Visit 1** Upon arrival at the trauma bay/ED (Day 0)	**Visit 2** 24 h following arrival at the trauma bay/ED (Day 1)	**Visit 3** 2–27 days following arrival at the trauma bay/ED	**Visit 4** End of study visit: Day 28 after arrival at the trauma bay/ED
Inclusion and exclusion criteria	x			
Randomization	x			
Interventions administered		x		
Baseline data				
Demographics		x	x*	x*
Medical history		x	x*	x*
Pre-arrival medications		x	x*	x*
Injury data		x	x*	x*
Obtain deferred consent from SDM or patient if recovered		x	x	x
**Primary endpoint**				
Total composite units of RBC + FP + platelets		x (24 h)		
**Secondary endpoints**				
Total number of units of RBCs		x (24 h)		
Thromboembolic events†		x	x	x
Ventilator-free days				x
**Additional endpoints**				
Efficacy endpoints				
Total and individual numbers of units and volumes of APBs (RBCs, FP, platelets) transfused		x (6 and 24 h)	x (Day 7)	
Total volume of crystalloids and other colloid use		x (6 and 24 h)		
Rescue use of rFVIIa		x		
Total FC use		x		
Laboratory tests, including thromboelastometry measurements, where available		x	x (Day 7)	
Days out of hospital within 28 days				x
Time to death				x
Safety endpoints				
AEs and SAEs		x	x	x
MOF (SOFA score)		x	x (daily)	x
ACS and LCS		x	x	x
Transfusion reactions from products transfused following arrival at the trauma bay/ED		x	x	x
Treatment-emergent events		x	x	x
Duration of ICU stay				x
All-cause mortality				x

* If not already collected.† Including leg Doppler ultrasound or other imaging as per clinical indications

ABP = allogeneic blood product; ACS = abdominal compartment syndrome; ED = emergency department; FP = frozen plasma; ICU = intensive care unit; LCS = limb compartment syndrome; MOF = multiple organ failure; RBC = red blood cells; rFVIIa = factor VIIa; SDM = substitute decision maker; SOFA = sequential organ failure assessment.

PROTOCOL SIGNATURES

This study is intended to be conducted in compliance with the protocol, Good Clinical Practice and applicable regulatory requirements.

Keyvan Karkouti MD

Coordinating Investigator and Sponsor Signature Date
Department of Anesthesia
Toronto General Hospital
200 Elizabeth Street, 3EN
Toronto, ON

Luis Teodoro da Luz MD

Principal Investigator Signature Date
Department of Surgery
Sunnybrook Health Sciences Centre
2075 Bayview Ave, H1.15
Toronto, ON

Hans-Peter Hucke

Statistician Signature Date
ERGOMED
Im Mediapark 2
D-50670 Cologne
Germany

TABLE OF CONTENTS

STUDY OUTLINE 2
FLOW CHART OF ASSESSMENTS 10
PROTOCOL SIGNATURES 11
LIST OF ABBREVIATIONS 16
1 INTRODUCTION 18
1.1 BACKGROUND 18
1.2 RATIONALE FOR CONDUCTING THE STUDY 22
1.3 BENEFIT-RISK STATEMENT 23
1.4 INVESTIGATOR (SPONSOR) 24
2 STUDY OBJECTIVES 25
2.1 PRIMARY OBJECTIVE 25
2.2 SECONDARY OBJECTIVES 25
3 INVESTIGATIONAL PLAN 26
3.1 PRIMARY AND SECONDARY ENDPOINTS 26
3.1.1 Primary Endpoint 26
3.1.2 Secondary Endpoints 26
3.2 OVERALL STUDY DESIGN AND PLAN 27
3.3 DISCUSSION OF STUDY DESIGN AND CHOICE OF CONTROL GROUP 30
3.3.1 Superiority Design 30
3.3.2 Choice of Primary Endpoint 31
3.3.3 Dose Rationale 31
3.3.4 Choice of Comparator 31
3.3.5 External Validity 31
3.3.6 Randomization and Baseline Differences 32
3.3.7 Recruitment and Informed Consent 32
3.3.8 Blinding of Investigational Medicinal Product (IMP) 32
3.3.9 Drop-outs and Crossovers 32
3.3.10 Outcome Assessments and Independent Data and Safety Monitoring Committee (IDSMC) 33
4 STUDY POPULATION 34
4.1 POPULATION BASE 34
4.1.1 Inclusion Criteria 34
4.1.2 Exclusion Criteria 34
4.2 PRIOR AND CONCOMITANT THERAPY 34
4.2.1 Permitted Concomitant Therapy 34
4.2.2 Forbidden Concomitant Therapy 35
4.3 WITHDRAWAL AND REPLACEMENT OF PATIENTS 35
4.3.1 Premature Patient Withdrawal 35
4.3.2 Patient Replacement Policy 35
4.4 ASSIGNMENT OF PATIENTS TO TREATMENT GROUPS 35
4.5 RELEVANT PROTOCOL DEVIATIONS 35
4.6 SUBSEQUENT THERAPY 35

5 INVESTIGATIONAL MEDICINAL PRODUCTS....37
5.1 CHARACTERIZATION OF INVESTIGATIONAL PRODUCTS.... 37
5.1.1 Fibryga....37
5.1.2 Octaplex....39
5.1.3 Red Blood Cells, Frozen Plasma and Platelets....41
5.1.4 Other Therapeutics Administered as Part of the MHP: Fibrinogen Concentrate42
5.1.5 Other Therapeutics Administered as Part of the MHP: Tranexamic acid....42
5.2 BLINDING, EMERGENCY ENVELOPES, AND BREAKING THE STUDY BLIND.... 43
5.3 TREATMENT COMPLIANCE.... 43
5.3.1 Drug Dispensing and Accountability....43
5.3.2 Assessment of Treatment Compliance....43
6 STUDY CONDUCT....44
6.1 STUDY PROCEDURE.... 44
6.1.1 Visit 1: Upon arrival at the trauma bay/ED....44
Visit 2: 24 hours following arrival at the trauma bay/ED....44
6.1.2 44
6.1.3 Visit 3: Days 2–27 following arrival at the trauma bay/ED....46
6.1.4 Visit 4: Day 28 post-arrival at the trauma bay/ED (in person if in hospital or by phone) 47
6.1.5 Maximum Visit Time Frame Used in this Study....47
6.2 DURATION OF STUDY.... 48
6.2.1 Planned Duration for an Individual Patient....48
6.2.2 Planned Duration for the Study as a Whole....48
6.2.3 Premature Termination of the Study....48
7 ASSESSMENTS AND METHODS....50
7.1 BASELINE DATA.... 50
7.1.1 Demographic and Baseline Characteristics....50
7.1.2 Medical History and Prior/Concomitant Medications....50
7.2 EFFICACY ASSESSMENTS.... 50
7.2.1 Transfusion Data....50
7.3 LABORATORY ASSESSMENTS.... 50
7.3.1 Test Parameters and Laboratories....50
7.3.2 Blood Sampling....51
7.3.3 Citrated Blood....51
7.3.4 Serum....51
7.3.5 Recording of Clinically Significant Abnormal Laboratory Values as AEs/ADRs 51
7.4 SAFETY ASSESSMENTS.... 52
7.4.1 Assessments for Safety Endpoints....52
7.4.2 Adverse Events....52
7.4.3 Serious Adverse Events....55
7.4.4 SAE Reporting Timelines....55
7.4.5 Incidence of thromboembolic events....56
7.4.6 Incidence and severity of MOF....57
7.4.7 Incidence of abdominal compartment syndrome and limb compartment syndrome58
7.4.8 Incidence of transfusion reactions....60
7.4.9 Other Relevant Safety Information....60
8 DATA HANDLING AND RECORD KEEPING....61

8.1 DOCUMENTATION OF DATA 61
8.1.1 Source Data and Records 61
8.1.2 Case Report Forms 61
8.1.3 Changes to Case Report Form (CRF) Data 61
8.2 INFORMATION TO INVESTIGATORS 62
8.3 RESPONSIBILITIES 62
8.4 INVESTIGATOR'S SITE FILE 62
8.5 PROVISION OF ADDITIONAL INFORMATION 63
8.6 INDEPENDENT DATA SAFETY MONITORING COMMITTEE 63
9 STATISTICAL METHODS AND SAMPLE SIZE 64
9.1 DETERMINATION OF SAMPLE SIZE 64
9.2 STATISTICAL ANALYSIS 65
9.2.1 Efficacy Analysis Plan 66
9.2.2 Safety Analysis Plan 67
9.2.3 Handling of Missing Data 69
9.3 RANDOMIZATION, STRATIFICATION, AND CODE RELEASE 69
9.4 ADAPTIVE DESIGN (INTERIM ANALYSIS) 69
9.5 SUBGROUP ANALYSIS 69
10 ETHICAL/REGULATORY, LEGAL AND ADMINISTRATIVE ASPECTS 71
10.1 ETHICAL/REGULATORY FRAMEWORK 71
10.2 APPROVAL OF STUDY DOCUMENTS 71
10.3 PATIENT INFORMATION AND INFORMED CONSENT 71
10.3.1 Deferred Consent 71
10.4 PROTOCOL AMENDMENTS 72
10.5 CONFIDENTIALITY OF PATIENT DATA 73
11 QUALITY CONTROL AND QUALITY ASSURANCE 74
11.1 PERIODIC MONITORING 74
11.2 AUDIT AND INSPECTION 74
12 REPORTING AND PUBLICATION 75
12.1 CLINICAL STUDY REPORT 75
12.2 PUBLICATION POLICY 75
13 LIABILITIES AND INSURANCE 76
14 REFERENCES 77
15 APPENDICES 83
APPENDIX 1. MASSIVE HEMORRHAGE PROTOCOL ACTIVATION CRITERIA COLLECTED DURING PROTOCOL DEVELOPMENT AT EACH POTENTIAL STUDY SITE 83
APPENDIX 2. DEFINITION OF MHP PACKS COLLECTED DURING PROTOCOL DEVELOPMENT AT EACH POTENTIAL STUDY SITE FOR THE INTERVENTION GROUP 84
APPENDIX 3. DEFINITION OF MHP PACKS COLLECTED DURING PROTOCOL DEVELOPMENT AT EACH POTENTIAL STUDY SITE, BASED ON THE STANDARD OF CARE 85
APPENDIX 4. THROMBOELASTOMETRY-GUIDED PARAMETERS FOR TARGETED THERAPY AT EACH STUDY SITE 86

APPENDIX 5. LABORATORY TEST-GUIDED PARAMETERS FOR TARGETED THERAPY AT EACH STUDY SITE 87
APPENDIX 6. CONSENTING PROCESS GUIDANCE DOCUMENT 88

LIST OF ABBREVIATIONS

Abbreviation	Description
A10	Amplitude at 10 Minutes
ABP	Allogeneic Blood Product
ACS	Abdominal Compartment Syndrome
ADR	Adverse Drug Reaction
AE	Adverse Event
ALP	Alkaline Phosphatase
ALT	Alanine Aminotransferase
aPTT	Activated Partial Thromboplastin Time
ATC	Acute Trauma Coagulopathy
ATE	Arterial Thromboembolism
BMI	Body Mass Index
CFT	Clot Formation Time
CI	Confidence Interval
CIHR	Canadian Institute of Health Research
CIMVHR	Canadian Institute for Military and Veteran Health Research
CPAP	Continuous Positive Airway Pressure
CRF	Case Report Form
CRO	Contract Research Organization
CT	Clotting Time
DRDC	Defense Research & Development Canada
DVT	Deep Vein Thrombosis
eCRF	Electronic Case Report Form
ED	Emergency Department
EDC	Electronic Data Capture
FC	Fibrinogen Concentrate
FIO_2	Fraction of Inspired oxygen
FP	Frozen Plasma
GCP	Good Clinical Practice
GCS	Glasgow Coma Scale
GLIM	Generalized Linear Model
GMP	Good Manufacturing Practice
HIV	Human Immunodeficiency Virus
IB	Investigator's Brochure
ICU	Intensive Care Unit
IDSMC	Independent Data Safety Monitoring Committee
IMP	Investigational Medicinal Product
INR	International Normalized Ratio
ISS	Injury Severity Score
ITT	Intention To Treat

IV	Intravenous
LCS	Limb Compartment Syndrome
LY30	Lysis at 30 Minutes
MAP	Mean Arterial Pressure
MCF	Maximum Clot Firmness
MedDRA	Medical Dictionary for Regulatory Activities
MHP	Massive Hemorrhage Protocol
MI	Myocardial Infarction
mITT	Modified Intention To Treat
ML	Maximum Lysis
MOF	Multiple Organ Failure
MRI	Magnetic Resonance Imaging
PaO_2	Partial pressure of Arterial Oxygen
PCC	Prothrombin Complex Concentrate
PE	Pulmonary Embolism
PEEP	Positive End-Expiratory Pressure
PI	Principal Investigator
pO_2	Partial pressure of Oxygen
PP	Per-Protocol
PRV	Pseudorabies Virus
PT	Prothrombin Time
RBC	Red Blood Cell
REB	Research Ethics Board
RR	Relative Risk
rFVIIa	Recombinant Factor VIIa
SAE	Serious Adverse Event
SAF	Safety Analysis Population
SAP	Statistical Analysis Plan
SBV	Schmallenberg Virus
SD	Standard Deviation
SDM	Substitute Decision Maker
SDV	Source Data Verification
SMQ	Standardized MedDRA Queries
SOFA	Sequential Organ Failure Assessment score
SUSAR	Suspected Unexpected Serious Adverse Reaction
TACO	Transfusion-Associated Circulatory Overload
TEAE	Treatment Emergent Adverse Event
TRALI	Transfusion-Related Acute Lung Injury
VTE	Venous Thromboembolism
WFI	Water for Injection

1 INTRODUCTION

1.1 Background

Acute Trauma Coagulopathy

Injury remains the leading cause of death among people aged 1-44 years worldwide [8]. Bleeding coupled with coagulopathy is a leading cause of in-hospital mortality in trauma [9-11], and compared with patients who do not have coagulopathy, those with coagulopathy have a three- to fourfold greater mortality and are up to eight times more likely to die within the first 24 hours following injury [12-16].

Coagulopathy in trauma is multi-factorial and is frequently associated with massive hemorrhage and clotting factor-deprived fluid resuscitation. Traditionally, trauma-induced coagulopathy was understood as the loss of procoagulant factors accompanying massive hemorrhage; dilution ensuing from aggressive crystalloid infusion and red blood cell (RBC) transfusion; and metabolic acidosis and hypothermia associated with hemorrhagic shock. However, evolving evidence suggests the existence of an early intrinsic coagulopathy, not explained by consumption and dilution, which is linked to an imbalance of the complex interplay in procoagulant, anticoagulant, and fibrinolytic pathways; and also associated with platelet and endothelial dysfunction [13,17-19]. Acute trauma coagulopathy (ATC) is associated with high transfusion requirements [20], longer intensive care unit (ICU) and hospital stays, more days requiring mechanical ventilation, and a greater incidence of multiorgan dysfunction.

Fibrinogen Replacement Therapies

Acquired fibrinogen deficiency (hypofibrinogenemia) is a major driver of ATC. Fibrinogen is a critical component of the coagulation cascade as it is both a precursor for fibrin which forms the basis of a firm clot, and is a cofactor that enhances platelet aggregation [21-24]. Fibrinogen is the first clotting factor to fall to critically low levels during life-threatening bleeding [25,26] and unlike other coagulation factors that have a large reserve margin [26], a modest drop in fibrinogen levels impairs coagulation and increases bleeding complications [27-34]. Low plasma fibrinogen levels at hospital arrival and late plasma administration are associated with increased transfusion requirements and mortality [35].

There are two primary options available for fibrinogen supplementation: cryoprecipitate and purified human-derived fibrinogen concentrate [21]. Cryoprecipitate is an allogeneic blood product (ABP) that is prepared by thawing fresh frozen plasma at 2 to 4ºC, harvesting the resultant precipitate by centrifugation, and then refreezing it at –20 ºC.

Cryoprecipitate has been historically the standard of care for fibrinogen supplementation in North America, but it has several important limitations. First, the amount of fibrinogen in each unit of cryoprecipitate is highly variable, ranging from 120 to 796 mg per unit [36]. To achieve adequate fibrinogen plasma level for hemostasis, cryoprecipitate is typically administered in 10-unit pools, which exposes patients to the risks of multiple allogeneic units. Second, thawing, reconstituting in saline, and pooling of cryoprecipitate is time consuming and labor intensive, which precludes rapid therapy. Third, cryoprecipitate is not a purified product and contains large amounts of contaminants such as fibronectin and platelet microparticles. These contaminants are not benign and may cause adverse outcomes such as thrombosis and organ dysfunction [36].

Purified human-derived fibrinogen concentrate (FC) is the second option for fibrinogen supplementation and is currently the mainstay of therapy for acquired hypofibrinogenemia in much of Europe. In North America, FC is currently only licensed for patients with congenital hypofibrinogenemia or afibrinogenemia. However, in Canada, 40% of cryoprecipitate use has been already replaced by FC.

The fibrinogen concentrate that will be used for this study, *Fibryga* (Octapharma, AG), is similar to cryoprecipitate in that it is derived from human plasma, but it has several important advantages [37]. First, it undergoes several virus removal and inactivation steps (nanofiltration [20-nm filter] and solvent detergent treatment), which remove contaminants and inactivate viruses. Thus, it is likely to have a lower risk of transmission of infectious agents. Indeed, a Canadian consensus statement from 2007 recommends adoption of such broad approaches to pathogen inactivation, which have a high likelihood of dealing with emerging pathogens [38]. Second, it is a highly purified concentrate, containing a consistent amount of fibrinogen (approximately 1 g per vial), and the response to therapy is potentially more predictable and more robust than for cryoprecipitate [39-41]. Third, since (unlike cryoprecipitate) the product can be administered immediately after it is reconstituted with sterile water, it allows for rapid fibrinogen supplementation. A recent randomized, controlled, pragmatic trial in the setting of acquired hypofibrinogenemia after cardiac surgery found FC to be non-inferior to cryoprecipitate for hemostasis, where the primary endpoint was cumulative number of ABPs transfused in the 24 hours after cardiopulmonary bypass [42]. No safety flags were identified in this trial, including no increase in the risk of thromboembolic complications (odd ratio 0.70; 95% CI 0.42–1.20).

<u>*Review of the literature*</u>

Successful use of fibrinogen replacement with FC in patients with acquired fibrinogen deficiency has been described in the literature. The E-FIT 1 randomized controlled feasibility trial by Curry et al. [43] investigated administration of FC (RiaSTAP™, CSL Behring) within 45 minutes of arrival in 48 bleeding trauma patients. The primary outcome was feasibility of administering the drug. The study demonstrated that it was not feasible (69%; 95% CI, 52–83% received FC across both groups). RiaSTAP™ was the product used in this study, which is stabilized with albumin, must be kept refrigerated, and therefore has a much longer reconstitution time than *Fibryga* (Octapharma). The authors recommended that other FC products with rapid reconstitution times should be explored in future studies. In a prospective multicenter observational study of 223 patients with acute bleeding by Weiss et al. [44], fibrinogen substitution was initiated at a median blood loss of 2.0 L and plasma fibrinogen level of 1.45 g/L. A median dose of 12 g fibrinogen (4 g administered as FC and 8 g from plasma) raised plasma fibrinogen levels to 2.19 g/L. There was a positive correlation between postoperative fibrinogen levels and survival.

Several systematic reviews have been published on the use of FC in patients with acquired bleeding [45-48]; one of these focused on FC in trauma patients. The authors [45] identified 12 studies on FC use in trauma; four case reports and seven retrospective studies and one observational study. No published RCTs were identified in this review. Despite methodological flaws, they suggest that FC in trauma may be associated with reduced blood product requirement. In the systematic review conducted by Wikkelso [47], bleeding patients in other settings were also included and meta-analysis was conducted. Overall, the systematic reviews included small RCTs mostly in cardiac surgery, but also in vascular surgery and urology. They consistently demonstrated that using FC was associated with improved viscoelastic testing parameters and reduced transfusion of ABP units. They also suggest that the use of FC in those populations was safe without an increase in thromboembolic complications.

Prior to conducting FiiRST-2, and based on evidence that FC may reduce blood product requirement in trauma patients, a randomized controlled feasibility trial (FiiRST-1) [49] was conducted at Sunnybrook Heath Sciences Centre to determine if it is feasible to administer FC within 60 min of hospital arrival in patients at risk of significant bleeding. FiiRST-1 showed that early FC infusion improved fibrinogen levels and improved coagulopathy as demonstrated by viscoelastic testing. There were no safety concerns related to early administration of 6 g FC in this population, including incidence of thromboembolic complications. The fibrinogen product used in this study was RiaSTAP™ (CSL Behring) which has a longer reconstitution time than *Fibryga*; however, the study concluded that infusion of 6 g FC within 1 hour of arrival at the trauma center was feasible and improved plasma fibrinogen concentration by approximately 1 g/L in a population of trauma patients at risk of significant hemorrhage. Additionally, no increased rates of thromboembolic complications were identified.

Coagulation Factor Deficiency in ATC

Consumption of other coagulation factors, along with hypofibrinogenemia, is another important component of ATC. To replenish these depleted coagulation factors and improve thrombin generation, two therapeutics, frozen plasma (FP) and prothrombin complex concentrate (PCC), are available. FP is currently the mainstay of therapy for patients with acquired coagulopathies in North America whereas PCC is the mainstay of therapy in much of Europe [50].

Frozen plasma is obtained from donated whole blood and contains all enzymatic coagulation factors, although some coagulation factor activity is lost during storage and processing (including freezing for storage and thawing prior to transfusion) [51]. Despite being a mainstay of therapy, there is little clinical trial evidence for the use of FP in bleeding trauma patients requiring coagulation factor replacement. The lack of evidence for the efficacy of FP is concerning in the context of the risks associated with its use [52,53]. FP can lead to adverse events (AEs) including allergic reactions in 1–3% of transfusions, and while most are not serious, life-threatening anaphylaxis can occur [54]. Transfusion-related acute lung injury (TRALI) is associated with plasma-containing components and is a leading cause of transfusion-related death [54,55]. Transfusion-associated circulatory overload (TACO), which occurs as a result of the large volume of transfusion required to achieve therapeutic effect with FP, occurs in approximately 5% of transfusions and is fatal in 2% [56]. Abdominal compartment syndrome (ACS) is also associated with volume overload in trauma patients [57]. FP transfusion is associated with transmission of infectious diseases, as FP is not usually filtered or treated with solvent/detergent [58]. These adverse effects are often dose dependent [59-62]. FP requires ABO blood group compatibility matching and thawing, which can either deplete scarce AB plasma stocks and/or delay therapy in attempts to provide ABO-identical plasma. The large volumes of FP needed to effectively raise thrombin generation can further delay time to hemostatic control and lead to substantial hemodilution, resulting in additional red blood cell (RBC) transfusions [63].

Prothrombin complex concentrate offers a potential alternative to FP for treating bleeding trauma patients requiring coagulation factor replacement. PCCs contain prothrombin and other enzymatic coagulation factors, the anticoagulant proteins C and S and anti-thrombin, and small amounts of heparin; they are routinely defined as 3-factor (that contain factors II, IX, and X) or four-factor (that contain factors II, VII, IX, and X) formulations [64]. PCCs such as *Octaplex* (Octapharma), the four-factor PCC used in this study, are purified from human pooled plasma, which is fractionated into cryoprecipitate and cryoprecipitate-free plasma fractions through a process of slow thawing, then eluted from cryoprecipitate-free plasma [64]. The production of PCCs

includes strict viral inactivation using solvents, detergents, pasteurization, nanofiltration, and vapor-heated treatment [64].

PCCs have several potential advantages over FP. Solvent/detergent treatment and filtering to remove viruses substantially reduces the risk of transmission of infectious agents with PCCs. Indeed, a Consensus Conference recommended in 2007 that pathogen reduction technologies should be implemented to improve the safety of transfusion when they became available [38]. Unlike FP, PCCs do not require ABO compatibility matching or thawing, and can therefore be prepared and administered more quickly. PCCs have the advantage of room temperature storage allowing for near patient storage and reducing the door-to-needle time in trauma. PCCs are associated with a substantially lower risk of TRALI (due to pooling of the source donor plasma), and also a lower risk of TACO [52], as substantially lower volumes of PCC are required than with FP to achieve dose-equivalence for increasing thrombin generation (for example, in a 70-kg patient, a standard dose of 25 IU/kg PCC would be administered in a volume of 80 mL, whereas a standard dose of 15 mL/kg FP would be administered in a volume of 1000 mL) [63]. PCCs contain standardized levels of coagulation factors and thus have a more predictable therapeutic effect than FP, whose coagulation factor concentrations vary depending on the characteristics of the donor [65,66].

PCCs do not contain the full balanced complement of procoagulants and anticoagulants present in FP [67] and it is therefore conceivable that they might be less effective, although *in vitro* studies suggest that PCCs may be more effective than FP in enhancing thrombin generation [63]. Also, because PCCs contain more procoagulants than anticoagulants, they may carry a higher risk of thrombotic events, disseminated intravascular coagulation and acute kidney injury [64,68-72], although the safety of using PCC has been documented in animal and human studies. Thromboembolic phenomena are one of the cited concerns regarding use of PCC. However, the true estimated incidence of thromboembolic events due to PCC remains unknown, because most studies were not powered to detect a difference. One study reported up to a 4% risk of thromboembolic complications with the use of four-factor PCC [73], with rates similar to patients administered plasma; however, a further clinical study found no thrombotic events in patients receiving PCC [74]. On the other side, a propensity-matched study [75] that enrolled 516 severely bleeding trauma patients (median Injury Severity Score [ISS] 29), comparing four-factor PCC + FC versus FP, did not report a difference between groups for thromboembolic complications (2.5% versus 1.2%, p=0.5). Similarly, a Phase 3b multicenter RCT in surgery patients also did not report higher rates of thromboembolic complications with use of four-factor PCC compared with FP for reversal of vitamin K in patients requiring surgery [76]. Finally, another study in bleeding cardiac surgery patients reported no difference in thromboembolic complications in patients receiving three-factor PCC + FP compared with FP alone [77].

<u>Review of the literature</u>

Successful use of coagulation management algorithms including the use of PCCs has been described in trauma [78-81] and also cardiovascular surgery [82,83]. The reversal of trauma-induced coagulopathy using first-line coagulation factor concentrates or fresh frozen plasma (RETIC) trial [80] described the use of goal directed therapy with FC, PCC, and factor XIII in a trauma setting. Patients were randomized to receive FC + PCC + Factor XIII or FP only, with both arms guided by rotational thromboelastometry (ROTEM). Patients in the FP group were more likely to undergo a massive transfusion and required more platelet transfusions, which led to the authors terminating the study early. Additionally, a recent study by Jehan et al. [75] enrolled bleeding trauma patients and used a propensity matched analysis. The authors demonstrated that use of four-factor PCC

in conjunction with FP was associated with a faster reversal of international normalized ratio (INR) and reduction in transfusion requirements as compared with FP alone. In another propensity-matched analysis, Zeeshan et al. [84] investigated the effect of receiving four-factor PCC + FP versus FP alone in 468 severely injured trauma patients. Compared to FP alone, PCC + FP administration was associated with decreased RBC transfusion requirement (6 versus 10 units; p=0.02), decreased number of FP units transfused (3 versus 6 units; p=0.01), and decreased mortality (17.5 versus 27.7%; p=0.01). Other less recent studies have also addressed the use of concentrates of clotting factors and FC in trauma. For example, Schöchl et al. [78] utilized FC and PCC in a retrospective analysis to treat hemorrhaging trauma patients, guided by ROTEM. The study was able to demonstrate a fast goal-directed FC therapy as first therapy step, with addition of PCC as needed. Patients had a favorable survival rate. The authors recommended further investigation of this strategy in prospective, randomized trials. Furthermore, in another retrospective analysis, Schöchl et al. [79] addressed the exposure of ABPs in trauma patients with this same goal-directed therapy by ROTEM, using FC and PCC, compared to another group of patients in whom only FFP was used. There was an improvement in the avoidance of RBC transfusion in 29% of patients in the FC-PCC group compared with only 3% in the FFP group (p<0.001). Additionally, transfusion of platelet concentrate was avoided in 91% of patients in the fibrinogen-PCC group, compared with 56% in the FFP group (p<0.001).

Current standard of care: the massive hemorrhage protocol

In North America the current standard of care is fibrinogen supplementation (with cryoprecipitate or FC), administered after verification of fibrinogen levels or clinician discretion, with other clotting factors replaced via FP administration as part of a massive hemorrhage protocol (MHP) in a 1:1 or 2:1 ratio of RBCs:FP [85]. The activation criteria for MHP has a general rationale, including patients bleeding or at risk of bleeding. No activation criteria are 100% sensitive or specific and vary between trauma centers [86]. Following activation of the MHP, the formula-driven resuscitation continues until the rate of hemorrhage is controlled, the MHP is terminated, and laboratory tests of coagulation can be performed to provide individualized decisions regarding further hemostatic therapy. Strategies for hemorrhage and coagulopathy treatment have changed significantly over the last decade. Prompt hemorrhage control, along with targeted coagulation factor replacement, are emerging as key components of trauma care. As discussed above, FC and PCC have several important advantages over cryoprecipitate or FP but there is a scarcity of data regarding the efficacy and safety on the use of PCC in combination with FC in hemorrhaging trauma patients. The FiiRST-2 study aims to understand if early use of FC and PCC in trauma patients at risk of massive hemorrhage will lead to superior patient outcomes.

1.2 Rationale for Conducting the Study

Preliminary data suggest that FC supplementation in trauma is associated with improved physiologic and clinical endpoints, but the safety and efficacy of early fibrinogen replacement in trauma patients has yet to be demonstrated in a large multi-center randomized controlled trial. Similarly, the evidence for use of PCC in bleeding trauma patients is still lacking, as described in Section 1.1. PCC has several advantages compared to the current standards of care, plasma, such as not requiring cross-matching and easy and rapid administration. PCC carries minimal risk of infection (as it undergoes pathogen reduction) and allergic reactions, and due to its low administration volume, carries minimal risk of transfusion-related circulatory overload or other volume related complications, such abdominal compartment syndrome, and acute respiratory distress syndrome.

A randomized, multicenter trial is necessary to demonstrate the impact of rapidly infusing FC and PCC as an early hemostatic therapy in bleeding trauma patients, as compared to standard MHP packs with FP administered in 2:1 to 1:1 ratio (RBC:FP). This trial will also provide safety data on early administration of FC and PCC as first-line hemostatic therapy in trauma care, and its impact on plasma fibrinogen levels, coagulation, and other clinical and transfusion endpoints. The finding that the use of FC and PCC decreases requirement for non-pathogen-reduced ABPs compared to a standard strategy would have several important consequences: (1) Possibility of administration of less fluid volume, leading to less hemodilution and fewer complications due to fluid overload; (2) Reduction of requirement for AB plasma per trauma patient reducing dependence on this product in short supply; (3) Potential ability to store FC and PCC in trauma bays at trauma centers to remove the time required for delivery to accelerate door-to-needle time; and (4) Potential to extend these products to be used in a pre-hospital setting (e.g., land/air transport) or in distant/austere/combat environments, which is particularly relevant in Canada with multiple remote geographical areas.

1.3 Benefit-Risk Statement

Substituting FP with FC and PCC is not expected to pose any material risks to the participants – this strategy is already standard of care in many European countries and a small number of studies suggest that this approach may be favorable [78-80,84]. Patients will only be included in the trial when the MHP is activated by the treating physician. In trauma, the MHP is activated when patients are severely bleeding. This means that these patients are experiencing ongoing loss of clotting factors and have a high chance of developing ATC, as described earlier in this protocol. Bleeding coupled with coagulopathy in trauma is responsible for a high incidence of in-hospital death within the first 24 hours post arrival. The expected mortality rate for trauma patients where the MHP has been activated is 28% at the largest level I trauma center in Canada (Sunnybrook Health Sciences Centre; data from January 2017 to August 2018). FC and PCC will replace most of clotting factors in these hemorrhaging patients to within normal levels.

Fibryga, the FC to be used in this study, is currently licensed for the treatment of congenital afibrinogenemia and hypofibrinogenemia. *Fibryga* has been shown to have comparable (and in some instances superior) pharmacokinetics, hemostatic effects, and safety profile to *RiaSTAP* (CSL Behring) [87,88], which is an approved (by Health Canada and FDA) purified human-derived fibrinogen concentrate for congenital afibrinogenemia and hypofibrinogenemia. In addition, *Fibryga* is logistically superior to *RiaSTAP* as it can be kept at room temperature and has a faster reconstitution time. Ongoing studies looking at the treatment of bleeding and surgery in patients with congenital fibrinogen deficiency have shown excellent efficacy and safety profile so far. *Fibryga* has been approved for the indication named above and for acquired fibrinogen deficiency in 15 European countries and in Switzerland. The experience to date with this concentrate has shown an excellent safety profile that is in all likelihood superior to that of cryoprecipitate due to the use of pathogen reduction strategies in the manufacturing process [21,89]. As discussed, FC is pathogen reduced and can be administered in predictable doses, making its administration likely to be both safer than, and at least as efficacious as, cryoprecipitate. Cryoprecipitate has been superseded by FC at all trauma centers participating in this trial.

Octaplex, the PCC to be used in this study, has been approved for use in patients anticoagulated with warfarin who are either bleeding or undergoing invasive procedures. In addition, PCC is approved for the treatment of

bleeding and perioperative prophylaxis of bleeding in acquired deficiency of the prothrombin complex coagulation factors, such as deficiency caused by treatment with vitamin K antagonists, or in case of overdose of vitamin K antagonists, when rapid correction of the deficiency is required.

Few studies have addressed PCC use in trauma. As described in Section 1.1 PCC has several advantages compared to the current standard of care, plasma, such as not requiring cross-matching and easy and rapid administration. PCC carries minimal risk of infection and allergic reactions, and due to its low administration volume, carries minimal risk of transfusion-related circulatory overload, acute respiratory distress syndrome, or abdominal compartment syndrome.

As mentioned before, both FC and PCC have important advantages over ABPs: they are faster to prepare, easier to administer, do not require ABO blood group compatibility matching, have a more predictable response, are less likely to cause volume-related complications (e.g., ACS), can be prepared and administered in the pre-hospital/transport phase of care, and a superior safety profile. Trauma patients are generally young and therefore survivors of massive transfusion would bear a greater burden from emerging blood pathogens, as compared to the majority of transfusion recipients who are over the age of 60 years. The finding that FC and PCC are superior to the standard of care (plasma) would make them the preferred option for trauma resuscitation, which has the potential to improve the quality and speed of care offered to trauma patients.

1.4 Investigator (Sponsor)

The Sponsor and Coordinating Investigator of this study is Keyvan Karkouti MD at the Department of Anesthesia, Toronto General Hospital, 200 Elizabeth Street, 3EN, Toronto, ON, Canada. This study will be funded by grants from the Canadian Institute of Health Research (CIHR), Canadian Institute for Military and Veteran Health Research (CIMVHR) and the Defense Research & Development Canada (DRDC). Octapharma Pharmazeutika Produktionsges GmbH, manufacturer of *Fibryga* and *Octaplex*, will provide funds for writing, data management, statistics, monitoring and IDSMC.

2 STUDY OBJECTIVES

2.1 Primary Objective

The primary objective of this study is to determine the impact of early co-administration of FC and PCC on the total number of ABPs transfused compared to the current standard of care (FP administered in a ratio-based plasma resuscitation).

2.2 Secondary Objectives

Secondary objectives include:

- To compare the hemostatic efficacy of the intervention with the standard of care, as measured by transfusion of ABPs, use of hemostatic interventions, and correction of coagulopathy based on laboratory tests
- To assess the safety of the intervention, focusing on arterial and venous thromboembolic complications

3 INVESTIGATIONAL PLAN

3.1 Primary and Secondary Endpoints

3.1.1 Primary Endpoint

The primary endpoint is to demonstrate superiority with respect to the composite number of all ABP units (RBCs, FP and platelets) transfused within 24 hours following arrival at the trauma bay/ED.

3.1.2 Secondary Endpoints

Secondary endpoints

- Total number of units of RBCs transfused within the first 24 hours following arrival at the trauma bay/ED
- Incidence of thromboembolic events, as defined by evidence of any of the following, from arrival at the trauma bay/ED, up to 28 days:
    - Deep vein thrombosis (DVT)
    - Pulmonary embolism (PE)
    - Myocardial infarction (MI) [1]
    - Ischemic stroke [2]
    - Arterial or venous thrombosis at other sites
- Ventilator-free days, defined as the number of days up to Day 28 following arrival at the trauma bay/ED on which a patient breathed without assistance (if period of unassisted breathing lasted at least 48 consecutive hours). Patients who die during study follow-up or require 28 or more days of mechanical ventilation will be assigned zero ventilator-free days [3]

Additional endpoints

Additional efficacy endpoints:

- Total and individual numbers of units and volumes (liters) of ABPs (RBCs, FP and platelets) transfused within 6 hours, 24 hours and within 7 days post arrival at the trauma bay/ED
- Total volume of crystalloids and other colloids administered within the first 6 and 24 hours following arrival at the trauma bay/ED
- Rescue use of hemostatic agents (fibrinogen concentrate and rFVIIa) within the first 24 hours following arrival at the trauma bay/ED
- Laboratory endpoints upon arrival (before drug administration), if measured, and following infusion of the investigational medicinal products (IMPs), as per each site protocol routine, measured within the first 24 hours and within 7 days following arrival at the trauma bay/ED:
    - Plasma fibrinogen levels
    - International normalized ratio (INR), prothrombin time (PT) and activated partial thromboplastin time (aPTT)
    - Hemoglobin and hematocrit levels

    - Platelet count
    - Base deficit, pH and lactate
    - Thromboelastometry values: EXTEM clotting time (CT), EXTEM A10, EXTEM maximum clot firmness (MCF), FIBTEM A10, FIBTEM MCF, and EXTEM LY30
- Days out of hospital within the first 28 days following arrival at the trauma bay/ED
- Time to death over the first 28 days following arrival at the trauma bay/ED

Additional safety endpoints:

- All documented adverse events (AEs) and serious adverse events (SAEs) during the first 28 days following arrival, including:
    - Multi organ failure (MOF) as measured by the Sequential Organ Failure Assessment (SOFA) score [4] daily during ICU stay for up to 28 days following arrival at the trauma bay/ED
        - Highest SOFA score and time of highest SOFA score
        - Change in SOFA score
    - Incidence of ACS, defined as sustained intra-abdominal pressure >20 mmHg with or without an abdominal perfusion pressure (APP) of <60 mmHg, that is associated with new organ dysfunction/failure [5]
    - Incidence of LCS [6]
    - Incidence of transfusion reactions as defined by the International Society of Blood Transfusion [7]
    - Incidence of treatment-emergent adverse events (TEAEs)
- Duration of ICU stay
- 29-day all-cause mortality

3.2 Overall Study Design and Plan

FiiRST-2 is a multicenter, randomized, controlled, superiority trial, utilizing a conventional, parallel group, two-armed, with an adaptive two-stage design, performed at eight Level 1 Trauma Centers in Canada. The study is designed to examine the effect on number of ABP units of early replacement of fibrinogen and clotting factors via FC and PCC in trauma patients with severe hemorrhage versus the current standard of care (ratio-based plasma resuscitation, and FC administered in response to low fibrinogen levels).

The study will aim to enroll 350 severely injured (penetrating or blunt) trauma patients who are at risk of significant hemorrhage. Due to the inherent variability in the primary endpoint and a yet substantial uncertainty about the effect size, an adaptive design approach will be used. For this, a planned unblinded interim analysis will be performed after about 120 patients have completed the study. This interim analysis will calculate the conditional power of test statistics and perform a sample size re-assessment. Hence, the final number of enrolled patients will depend on the sample size re-calculation.. The sponsor PI, all study PIs, and other personnel involved in the study will remain blinded, only the IDSMC will review unblinded data and advise the sponsor on possible adaptations in sample size or other study aspects. Appropriate type I error adjustments due to the adaptation will be specified in a separate statistical analysis plan.

Upon arrival at the trauma bay/ED when the MHP has been activated according to the MHP activation criteria at each study site (within the first hour post-arrival), patients will be enrolled. Once eligibility is confirmed, the blood bank medical laboratory technologist will randomize the patient to one of two groups: the intervention group, who will receive FC and PCC or the control group who will receive ratio-based plasma resuscitation with FP and FC administered in response to low fibrinogen levels, as per the standard of care at the study site (Figure 1).

Figure 1. Study Flow

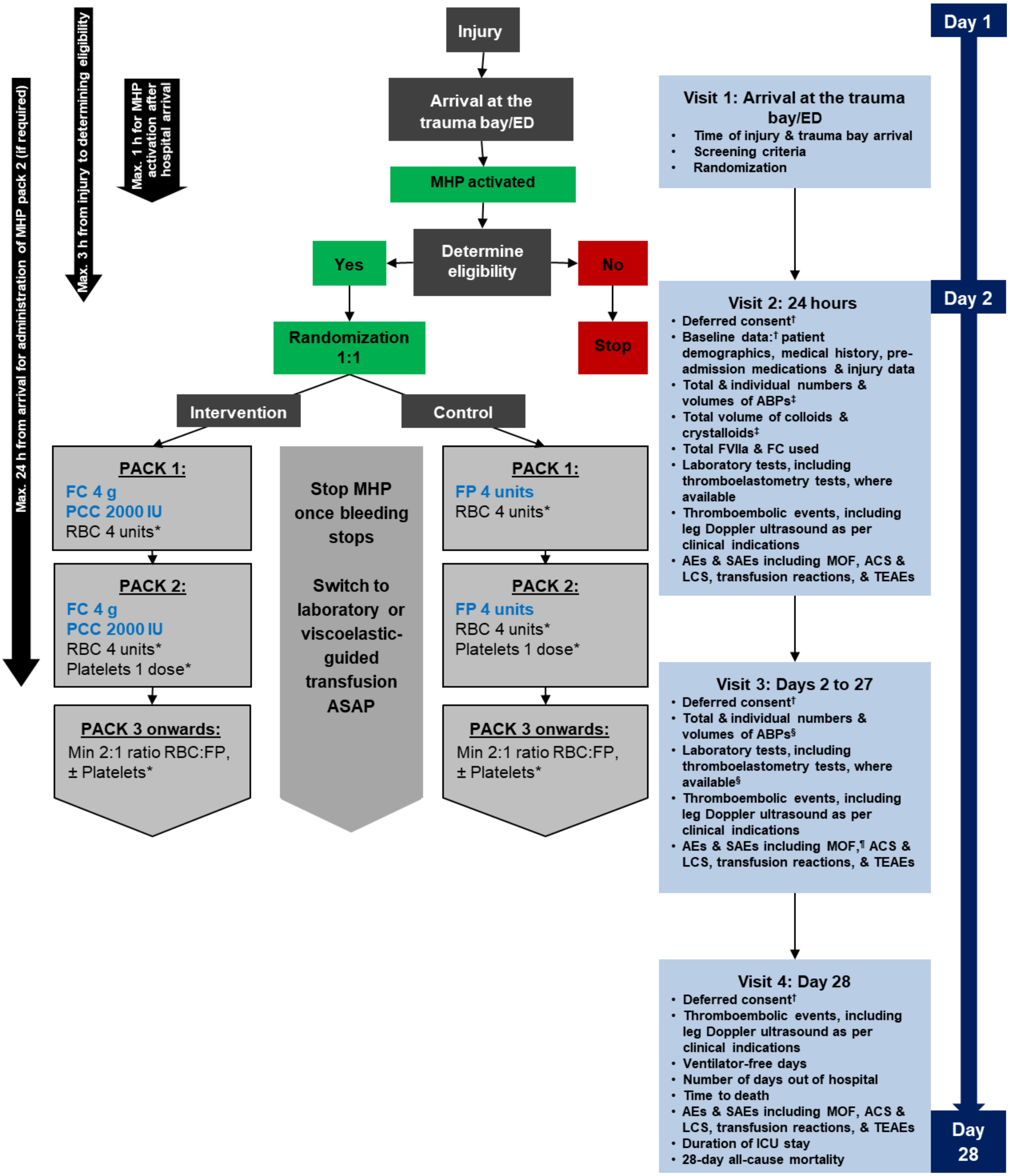

* Administration of all non-investigational products at the discretion of the clinical team, according to the hemodynamic status of the patient and/or laboratory results or viscoelastic test results
† If not collected at an earlier time-point
‡ Collected at 6 hours and 24 hours after arrival at the trauma bay/ED
§ Collected at Day 7 after arrival at the trauma bay/ED
¶ Collected daily for patients still in the ICU
ABP = allogeneic blood products; ACS = abdominal compartment syndrome; AE = adverse event; ED = emergency department; FC = fibrinogen concentrate; FP = frozen plasma; ICU = intensive care unit; LCS = limb compartment syndrome; MHP = massive hemorrhage protocol; MOF = multi organ failure; PCC = prothrombin complex concentrate; RBC = red blood cells; rFVIIa = recombinant factor VIIa; RBC = red blood cells; SAE = serious adverse events; TEAE = treatment-emergent adverse event.

Patients in both groups will receive MHP treatment packs as indicated by the study site MHP initiation criteria. The first two packs will contain therapeutics according to the group assignment: patients in the intervention group will receive FC (*Fibryga*) and PCC (*Octaplex*) and patients in the control group will receive FP in the first and second packs, with platelets in the second pack. In both groups, RBCs will be included as part of the first and second MHP packs, and 1 dose of platelets will also be included as part of the second MHP pack. Both RBCs and platelets will be administered according to the clinical situation and/or lab results. In the control group, FC may be administered if hypofibrinogenemia (fibrinogen level below 1.5–2.0 g/L or FIBTEM A10 below 8–12 mm) is identified as part of routine testing, at the discretion of the clinical team. The maximum time frame for administration of the second MHP pack (if required), is 24 hours from arrival at the trauma bay/ED or termination of the MHP (whichever comes first). If a third pack is required, and thereafter, patients in both groups will receive MHP packs according to MHP guidelines at each participating site or revert to a laboratory or viscoelastic-guided transfusion as per the local guidelines if hemorrhage control is achieved and the MHP is terminated. The MHP should be terminated once bleeding is controlled and the MHP criteria are no longer met. Thereafter, transfusions should be based on the results of laboratory testing and rate of bleeding.

The primary efficacy outcome will be the number of ABP units (RBCs, FP and platelets) administered during the first 24 hours following arrival at the trauma bay/ED, with the primary comparisons being in the modified intention-to-treat (mITT) population.

Safety outcomes will be measured for the first 28 days following arrival at the trauma unit, which is the duration of participation of each patient in the trial.

3.3 Discussion of Study Design and Choice of Control Group

3.3.1 Superiority Design

A superiority design was selected because FC and PCC have important advantages over ABPs: they are faster to prepare, easier to administer, do not require ABO blood group compatibility matching, have a more predictable response, are less likely to cause volume-related complications (e.g., ACS), can be prepared and administered in the pre-hospital/transport phase of care, and a superior safety profile (as it undergoes pathogen reduction). The finding that FC and PCC are superior to the standard of care, with plasma and replacement of fibrinogen based on laboratory fibrinogen levels, would make them the preferred option for trauma resuscitation, which has the potential to improve the quality and speed of care offered to trauma patients, leading to changes in evidence-based guidelines for early hemostatic therapy in trauma. A consensus conference held in 2007 [38] recommended implementation of pathogen reduction strategies as soon as they were available and found to be efficacious to mitigate the risks of transfusion-transmitted pathogens. The superiority design of

the FiiRST-2 trial was chosen due to the impact on clinical practice and logistical benefit that FC and PCC may offer. However, we believe that even if a non-inferiority design had been chosen, its positivity would still be acceptable to recommend the adoption of the two interventions.

3.3.2 Choice of Primary Endpoint

The primary efficacy endpoint is the total number of ABP units (RBCs + FP + platelets) administered during the 24 hours following arrival at the trauma bay/ED. This is a clinically relevant outcome that has been used in previous randomized trials in bleeding patients in a cardiac surgical setting [77,90]. Moreover, it is a primary outcome that has been accepted by the European Medicines Agency (EMA) for a major multi-center clinical study using FC in complex cardiac surgery patients (ClinicalTrials.gov Identifier: NCT01475669).

3.3.3 Dose Rationale

The first two MHP packs given to patients in the intervention group will contain 4 g FC and 2000 IU PCC. A systematic review on the use of FC in trauma described doses ranging from 5 g to a total of 16 g during early hospital management [45]. Similar dose ranges have also been used in non-trauma bleeding patients [46,47]. An initial dose of 4 g of FC will be used as this seems to be an average dose that has been used in recent studies, including in our FiiRST-1 feasibility trial (6 g) [49] and in the large RCT conducted in cardiac surgery patients, the FIBRES trial (4 g) [90]. The safety of administering FC has been described in the literature and of up to 14 g total dose in less than 5 minutes in aortic surgery trials [42,91].

The safety of PCC has also been described [35,92]. For PCC, the usual dose is 25 to 50 IU/kg [35] and in Canada the 2011 National Advisory Committee on Blood and Blood Products recommendations advise a dose of 2000 IU for major bleeding [93], which will be used in our trial without modification for patient weight due to difficulty obtaining an accurate weight in the trauma setting.

3.3.4 Choice of Comparator

The trial will not include a placebo arm because delaying fibrinogen and coagulation factor supplementation in bleeding patients would expose them to the negative consequences of excessive blood loss, is not consistent with standard practice, and would withhold an effective treatment from patients and thus be unethical. Moreover, the question being addressed does not meet any of Freedman's five conditions that would justify the use of a placebo control, which are: 1) no standard treatment exists; 2) standard treatment is not better than placebo; 3) standard treatment is a placebo or no treatment; 4) new evidence has shown uncertainty of the risk-benefit profile of the standard treatment; and 5) effective treatment is not readily available due to cost or supply issues [94,95]. Additionally, the 8 participating sites currently use a 2:1 to 1:1 ratio as the standard of care in their MHPs.

3.3.5 External Validity

This study will be performed in eight hospitals with different characteristics and volumes of trauma activations. Moreover, patients will be recruited and randomized after the clinical team activates the MHP, as determined by local hospital practice. The change to routine practice is the administration of coagulation factor concentrates (FC and PCC) instead of the current standard of care (FP administered in a ratio-based plasma resuscitation and FC administered in response to low fibrinogen levels). Thus, patient management in the

control group will reflect current practice and in the intervention group will reflect how FC and PCC will be used in practice. For these reasons, the study will have good external validity. Numerous European studies have used solely goal-directed use of FC and PCC based on TEG and ROTEM results; the trial investigators and site-investigators currently have a 30–60 minute delay to the first ROTEM or laboratory based assays (INR, PTT) and therefore preemptive coagulation factor replacement would be required at all sites, precluding this type of trial design.

3.3.6 Randomization and Baseline Differences

Given the large size of the study and random patient assignment stratified by center, study groups should be well balanced with respect to important clinical variables. The random allocation schedule will be prepared by a biostatistician not involved in the conduct of the trial, and neither the individual randomizing nor any of the health care providers will know which treatment will be assigned to the patient when the MHP is activated.

3.3.7 Recruitment and Informed Consent

Due to the emergency nature of the condition being studied (i.e., trauma patients who are at risk of significant hemorrhage), the trial will include only patients who are incapable of providing informed consent at the time the therapy is needed and in whom delays in obtaining surrogate consent can be severely detrimental to their well-being. Thus, we will employ a deferred informed consent approach. This consent process meets the criteria for alterations of the informed consent according to the Tri-council policy statement for the ethical conduct for research involving humans, as outlined in **Section 10.3**.

3.3.8 Blinding of Investigational Medicinal Product (IMP)

Given that the products have quite different physical differences, it is not possible to blind the treating clinicians to group assignment. Clinicians not involved in the acute resuscitation period (MHP activations usually last under 4 hours) and outcome assessors will remain blinded by using a generic product label in the patient chart and/or the electronic product name (i.e., FiiRST-2 MHP pack 1 and pack 2, rather than specifying type of product used). The first pack will be sealed, and the clinical team will be instructed to refrain from opening the first pack until the decision is made to transfuse clotting factor replacement.

3.3.9 Drop-outs and Crossovers

Drop-outs: The FiiRST-1 feasibility study had a 10% rate of patient drop-outs (exclusions post randomization). The sample size for the FiiRST-2 study has been calculated to compensate for a drop-out percentage of up to 15%.

Product switching: Other than the MHP pack order being cancelled due to MHP termination after achieving bleeding control, all patients will be treated according to the randomization schedule for the first two MHP packs. To ensure minimal product switching, instructions will be entered into the blood bank information system to dictate the randomization product for the first two packs and will flag the laboratory technologists if attempts are made to override the instruction. In very rare circumstances (e.g., after catastrophic bleeding), clinicians may opt to switch from one therapy to the other during the treatment period. The reasons for this request will be collected and described. It is anticipated that the number of product switches will be very few (<1%).

3.3.10 Outcome Assessments and Independent Data and Safety Monitoring Committee (IDSMC)

An IDSMC will review accumulating safety, endpoint, and other study data (recruitment, retention and compliance, data quality and timeliness, risk vs. benefit). The function of the IDSMC will be to protect and serve the recruited patients particularly pertaining to patient safety as well as to assist and advise the Sponsor on medical questions and issues of study conduct and continuation. The IDSMC will be independent of the investigating team and the Sponsor in operating and formulating recommendations. The IDSMC full role will be detailed in the IDSMC Charter. As the FiiRST-2 trial has an adaptive design, an interim analysis will be conducted when approximately 120 patients have been enrolled, whereby the members of the IDSMC will be entitled to review unblinded interim results.

4 STUDY POPULATION

4.1 Population Base

Approximately 350 adult trauma patients for whom the MHP has been activated will be randomized, with approximately 175 assigned to each of the two treatment groups. Due to the large variability in the mean number of units of ABPs transfused to this population in the literature and a yet substantial uncertainty about the effect size, we will use an adaptive design approach. An unblinded analysis will be conducted by the IDSMC when approximately 120 patients have been enrolled. The analyses will account for differences in the number of ABP units transfused, the variability and effect sizes across centers, to be able to re-estimate the final study sample size. Therefore, the final number of enrolled patients may be greater or less than 350 depending on the sample size recalculation. The sponsor, PI and others involved in the study will remain blinded.

4.1.1 Inclusion Criteria

Severely injured adult trauma patients who meet all following criteria:

1. Estimated age greater than 16 years old
2. Severely injured (penetrating or blunt) trauma patients
3. Triggered MHP within first hour of arrival at the trauma bay/ED. Activation criteria for the MHP at each site are listed in in Appendix 1.

4.1.2 Exclusion Criteria

Patients who meet any of the following criteria are *not* eligible for the study:

1. Have received more than 2 U RBCs during the pre-hospital phase of care
2. Have received more than 2 U RBCs in the trauma bay/ED before activation of the MHP
3. Have an elapsed time from injury of more than 3 hours
4. Have a penetrating traumatic brain injury with Glasgow Coma Scale (GCS) of 3
5. Are suspected or known to be on anticoagulants in the last 7 days
6. Have known congenital or acquired bleeding disorder
7. Have a known pregnancy
8. Refuse blood transfusion due to religion or other reasons
9. Previous history of heparin induced thrombocytopenia (HIT)

4.2 Prior and Concomitant Therapy

Details on medications taken within 1 week before enrolment and any concomitant medications taken during the study must be recorded in the case report form (CRF).

4.2.1 Permitted Concomitant Therapy

Concomitant administration of any therapies required as part of standard patient care is permitted but must be recorded in the CRFs. We will record all hemostatic drugs or products administered. In addition, concomitant medications used to treat SAEs will be reported throughout the duration of follow-up (up to postoperative day 28). The use of tranexamic acid within 3 hours of injury is the standard of care at all 8 participating hospitals.

4.2.2 Forbidden Concomitant Therapy

Patients are not permitted to enter the study if they are suspected or known to have received anticoagulants in the last 7 days or have received more than 2 U of RBCs before randomization prior to hospital arrival /or more than 2 U of RBCs between hospital arrival and MHP activation.(including during pre-hospital RBC transfusions).

4.3 Withdrawal and Replacement of Patients

4.3.1 Premature Patient Withdrawal

Patients or their substitute decision makers (SDM) have the right to withdraw from the study at any time for any reason, without the need to justify their decision. The Investigator also has the right to withdraw patients in case of AEs, poor compliance, or other reasons. Since an excessive rate of withdrawals can render the study non-interpretable, unnecessary withdrawal of patients will be avoided. For any withdrawals after study entry, the Investigator will obtain all the required details and document the reason(s) for discontinuation. If the reason for withdrawal of a patient is an AE, the main specific event or laboratory test will be recorded, and the Investigator will make thorough efforts to clearly document the outcome.

4.3.2 Patient Replacement Policy

Patients withdrawn from the study for safety reasons will not be replaced.

4.4 Assignment of Patients to Treatment Groups

Eligible patients will be randomly assigned to the intervention group or the control group. Randomization will occur via a computer-generated random-block size allocation sequence generated by a biostatistician. In order to assure balanced groups, the randomization will be stratified by hospital site. Sealed opaque envelopes containing these numbers for treatment allocation will be maintained in the blood banks of the participating sites.

The medical laboratory technologist from the blood bank will randomize patients to one of the two groups once eligibility has been confirmed. Patients will be identified using a sequential numbering system.

Subjects/patients are not permitted to re-enroll in the study. Enrolled patients will have a patient instruction in the blood bank information system to prevent re-enrollment on a subsequent trauma admission.

4.5 Relevant Protocol Deviations

In the case of any major protocol deviation, the Sponsor and the Principal Investigator will decide on the further participation of the patient in this study.

4.6 Subsequent Therapy

After the first two MHP packs have been administered, i.e., from MHP pack 3 onwards, patients in both groups may receive ABPs in each MHP pack according to MHP guidelines at each participating site (Table 2), at the discretion of the clinical team. The sites will keep their MHP standards with further MHP packs or revert to a

laboratory or viscoelastic-guided transfusion once bleeding is controlled, as per the local guidelines. The investigators at participating study sites have been requested not to alter the contents of MHP packs 3 and later during the study period.

Table 2 MHP Packs 3 and Following at Each Participating Site

Site	Red blood cells (U)	Plasma (U)	Platelets (dose)*	Fibrinogen concentrate (g)
Sunnybrook Health Sciences Centre	4	2	0.5 (in every other pack)	-
Saint Michael's Hospital	4	4	-	-
Montreal General Hospital	5	4	1	-
Foothills Medical Centre	6	4	1	-
Vancouver General Hospital	6	6	1	-
The Ottawa Hospital	6	6	1	-
Hamilton General Hospital	4	2	1	-
London Health Sciences Centre	4	4	1	-
Kingston Health Sciences Centre	4	4	1	-
Vancouver General Hospital	4	2	1**	4g+

* 1 dose = 4 U of pooled or single donor platelets; **or as ordered by MD; + Only given if not in Packs 1 & 2

5 INVESTIGATIONAL MEDICINAL PRODUCTS

5.1 Characterization of Investigational Products

5.1.1 Fibryga

Fibryga is a highly purified, lyophilized human plasma fibrinogen concentrate without residual albumin. The manufacturing process of *Fibryga* includes two dedicated virus inactivation/removal steps, i.e., solvent/detergent treatment and nanofiltration.

The solvent/detergent treatment mode of action causes enveloped viruses to be irreversibly destroyed. These include the most transfusion-relevant viruses, such as human immunodeficiency virus types l and 2 (HIV-1, HIV-2), hepatitis B virus (HBV) and hepatitis C virus (HCV), and many other adventitious agents, e.g., newly emerging enveloped viruses, such as West Nile virus (WNV).

The Planova 20N filter was specifically developed by Asahi Kasei Pharma Corp. to remove infectious agents from protein solutions on the basis of their size. Thus, this nanofiltration step is in principle effective for removing even very small enveloped and non-enveloped viruses. Nanofiltration may be the only method to date permitting efficient removal of enveloped and non-enveloped viruses under conditions where 90–95% of protein activity is recovered [96]. Other precautions against viral transmission include: selection of plasma donors, screening of donations and plasma pool, as well as quality control measurements of the final product.

<u>Composition of Fibryga</u>

Fibryga is a human plasma-derived fibrinogen concentrate for intravenous (IV) use. Its ingredients are listed in Table 3.

Table 3 Composition of *Fibryga*

Ingredients	Quantity per mL reconstituted solution, mean values	Standard
**Active ingredient**		
Fibrinogen as clottable protein	20 mg	Ph. Eur.
**Excipients**		
Sodium chloride	6 mg	Ph. Eur.
Sodium citrate dehydrate	1.5 mg	Ph. Eur.
Glycine	10 mg	Ph. Eur.
L-arginine hydrochloride	10 mg	Ph. Eur.

Ph. Eur. = Pharmacopoeia Europaea

Fibryga is a powder for solution for injection supplied in labeled 100-mL vials and provided by Octapharma. *Fibryga* will be reconstituted with 50 mL sterile water for injections (WFI) produced according to good manufacturing practice (GMP) and provided by the clinical site.

The final product is released by the responsible Octapharma Quality Control Department, according to a defined final product specification.

Conditions for Storage and Use

This IMP has to be stored at room temperature (not more than 25°C) and protected from light. The product must not be frozen. The Investigator/authorized personnel at the site will ensure that the IMP is stored in appropriate conditions with restricted access and in compliance with national regulations.

Dose and Dosing Schedule

Intervention group: Patients randomized to the intervention group will receive 4 g *Fibryga* in the first MHP pack and 4 g *Fibryga* in the second MHP pack, if required. The maximum time frame for administration of the second MHP pack (if required), is 24 hours from arrival at the trauma bay/ED. If a third MHP pack is required, and thereafter, FC (*Fibryga*) will be administered if the fibrinogen level drops below 1.5–2.0 g/L at the discretion of the clinical team or based on conventional laboratory test results or viscoelastic methods (FIBTEM A10 <8–10 mm) (see Section 5.1.4 for further details).

Control group: For the duration of the study, patients randomized to the control group will receive FC (*Fibryga*) at the discretion of the clinical team, or, most commonly, as part of the current guidelines in the participating sites, if the fibrinogen level drops below 1.5–2.0 g/L (based on conventional laboratory results), or based on results of viscoelastic testing (see Section 5.1.4 for further details). Currently, at the participating sites, FC is replaced after a low level is identified, which usually has a turnaround time of at least 1 hour. It is expected that the replacement of FC will occur only after the eighth unit of RBCs has been administered.

For patients in the intervention group, clinicians will be instructed to administer all of the investigational products, including *Fibryga,* in the first pack before moving onto the second pack. If the second pack is opened, clinicians will be instructed to administer all of the investigational products contained within, before moving on to the third pack. Not administering all of the investigational products in the first or second packs, once started, will be considered a protocol deviation.

Preparation

Fibryga will be delivered to the trauma bay/ED at room temperature with a tamper-proof seal and opened just before products are administration to the patient. A member of the trauma team will be assigned to prepare and administer, as per standard of care procedures. Each 1 g-vial of *Fibryga* will be reconstituted with 50 mL WFI at room temperature (not more than 25°C). *Fibryga* dissolves at room temperature to an almost colorless and slightly opalescent solution within 10 minutes. If the solution is cloudy or contains particulates, it should not be used.

Method of Administration

The 4 g-doses of *Fibryga* will be administered immediately after reconstitution over 4 minutes, through a free-flowing IV by syringe injection, using one syringe per 1 g/50 mL *Fibryga*. *Fibryga* should not be mixed with other medicinal products or crystalloid intravenous solutions.

Packaging and Labeling

The label will comply with the Canadian national requirements. The product will be distributed to the sites by Canadian Blood Services in all sites except Montreal, where the product will come directly from Octapharma only for study patients.

Several batches of IMP may be used throughout the study. The batch numbers will be recorded in the CRFs.

5.1.2 Octaplex

Octaplex is a product derived from human plasma containing the coagulation factors II, VII, IX, and X and proteins C and S. It is manufactured by chromatographic purification of cryoprecipitate-poor plasma.

The *Octaplex* manufacturing process includes two dedicated virus inactivation/removal steps, by way of a solvent/detergent viral inactivation process and a virus removal nanofiltration step. The solvent/detergent treatment causes enveloped viruses such as PRV, SBV, and HIV-1 to be irreversibly destroyed. Nanofiltration removes infectious agents from protein solutions on the basis of their size may be the only method to date permitting efficient removal of enveloped (e.g., HIV-1, SBV, PRV, bovine viral diarrhea virus) and non-enveloped viruses (e.g., hepatitis A virus) under conditions where 90–95% of protein activity is recovered [96]. Other precautions against viral transmission include: selection of plasma donors, screening of donations and plasma pool, as well as quality control measurements of the final product.

Composition of Octaplex

Octaplex is a human PCC for intravenous (IV) use. Its ingredients are listed in Table 4.

Table 4 Composition of *Octaplex*

Ingredients	Quantity per 20-mL vial	Quantity per 40-mL vial
**Active substances**		
Human coagulation factor II	280–760 IU	560-1520 IU
Human coagulation factor VII	180–480 IU	360-960 IU
Human coagulation factor IX	500 IU	1000 IU
Human coagulation factor X	360–600 IU	720-1200 IU
**Further active ingredients**		
Protein C	260–620 IU	520–1240 IU
Protein S	240–640 IU	480–1280 IU
**Clinically relevant non-medicinal ingredients**		
Heparin	80–310 IU	160–620 IU
Sodium citrate	17.0–27.0 mmol/L	17.0–27.0 mmol/L

IU, international units

Further excipient: Solvent (Water for Injection)

Small amounts of the S/D reagents TNBP (5 µg/ml) and Polysorbate 80 (50 µg/mL) may remain in the finished product. These substances are added during the manufacturing process because of their capacity to inactivate lipid-enveloped viruses

Octaplex is a powder that is reconstituted with sterile water solution for injection and is supplied in labeled glass vials and provided by Octapharma. *Octaplex* will be reconstituted with 20-mL or 40-mL (as per the manufacturer's instructions) sterile WFI produced according to GMP and provided by the clinical site.

The final product is released by the responsible Octapharma Quality Control Department, according to a defined final product specification.

Conditions for Storage and Use

This IMP has to be stored at room temperature (not more than 25°C) and protected from light. The product must not be frozen. The Investigator/authorized personnel at the site will ensure that the IMP is stored in appropriate conditions with restricted access and in compliance with national regulations.

Dose and Dosing Schedule

Intervention group: Patients randomized to the intervention group will receive 2000 IU *Octaplex* in the first MHP pack and 2000 IU *Octaplex* in the second MHP pack, if required. The maximum time frame for administration of the second MHP pack (if required), is 24 hours from arrival at the trauma bay/ED.

PCC is not currently used as standard practice during the initiation of resuscitation at any of the 8 participating trauma centers and patients in the control group will not be permitted to receive PCC. PCC is not expected to be administered to patients in the study other than to patients in the intervention group in MHP packs 1 and 2.

For patients in the intervention group, clinicians will be instructed to administer all of the investigational products, including *Octaplex,* in the first pack before moving onto the second pack. If the second pack is opened, clinicians will be instructed to administer all of the investigational products contained within, before moving to the third pack. Not administering all of the investigational products in the first or second pack, once started, will be considered a protocol deviation.

Preparation

Octaplex will be delivered to the trauma bay/ED at room temperature with a tamper-proof seal and opened just before products are administration to the patient. A member of the trauma team will be assigned to prepare and administer, as per standard of care procedures. Each 500 IU vial of *Octaplex* will be reconstituted with 20 mL WFI, as per the manufacturer's instructions, at room temperature (not more than 25°C). *Octaplex* dissolves at room temperature to an almost colorless and slightly opalescent solution within 10 minutes. If the solution is cloudy or contains particulates, it should not be used.

Method of Administration

Octaplex will be administered intravenously, immediately after reconstitution as recommended, via free flowing IV syringe injection, over 5 minutes, using one syringe per 1000 IU/40 mL *Octaplex*. *Octaplex* should not be mixed with other medicinal products or crystalloid intravenous solutions. Total infusion time will be 10 minutes (5 minutes for each 1000 IU).

Packaging and Labeling

Octaplex will be packaged and labeled for the trial by Octapharma. The label will comply with the Canadian national requirements. The product will be distributed to the sites by Canadian Blood Services in all sites except Montreal, where the product will come directly from Octapharma only for study patients.

Several batches of IMP may be used throughout the study. The batch numbers will be recorded in the CRFs.

5.1.3 Red Blood Cells, Frozen Plasma and Platelets

Dose and Dosing Schedule

Intervention group: 4 g *Fibryga* and 2000 IU *Octaplex* will be released as part of the first and second MHP packs. Details of interventional products and APBs administered to patients in the intervention group are described in Appendix 2.

Control group: 4 U FP will be released as part of the first and second MHP packs. Details of APBs administered to patients in the control group are described in Appendix 3.

Concomitant therapy: In both groups, 4 U RBC will be included as part of the first and second MHP packs, and 1 dose platelets (4 U of pooled random donor or single donor apheresis) will also be included as part of the second MHP pack. Both RBCs and platelets will be administered according to the clinical situation and/or lab results as per the discretion of the clinical team.. The second MHP pack will be released at the request of the clinical team, but clinicians will be instructed to administer all of the investigational product (*Fibryga/Octaplex* or FP) in the first pack before moving onto the second pack. Similarly, if the second pack is opened, clinicians will be instructed to administer all of the investigational products contained within, before moving to the third pack. Not administering all of the investigational products in the first or second packs, once started, will be considered a protocol deviation.

Administration of all non-investigational products will be at the discretion of the clinical team according to the hemodynamic status of the patient and/or laboratory results or viscoelastic test results. While platelets will be routinely included in the second pack, clinicians can request platelets outside of the packs (e.g., for patients on antiplatelet therapy or with marked thrombocytopenia).

Patients in the intervention group may receive FP in the third and subsequent packs. If a third MHP pack is required, and thereafter, MHP packs will contain ABPs (RBCs, platelets and plasma) according to guidelines at each participating site (Table 2, Appendix 2 and Appendix 3 or revert to laboratory- (Appendix 5) or viscoelastic-guided transfusion (Appendix 4) as per the local guidelines once bleeding is controlled. The MHP should be terminated once bleeding is controlled and the MHP criteria are no longer met. Termination of the MHP may occur before infusion of the contents of both packs 1 and 2.

The maximum time frame for administration of the second MHP pack (if required) for both groups is 24 hours from arrival to the trauma bay/ED or termination of the MHP (whichever comes first).

Preparation

RBCs, FP and platelets will be provided by the Canadian Blood Services and HemaQuebec and stored and prepared by the blood bank according to current standards. RBCs and FP will be provided in a cooler to maintain temperature at 1–10°C and platelets will be provided at room temperature in transport bags. Packs will be delivered to the trauma bay/ED with a tamper-proof seal and opened just before products are administered to the patient.

Method of Administration

RBCs, FP and platelets will be infused as per standard hospital protocols at the participating institutions. For the first two MHP packs in the intervention group, RBCs will be administered concomitantly to FC and PCC as per each participating site local protocols and at the discretion of the physician clinical team according to the hemodynamic status of the patient and/or laboratory results or viscoelastic test results.

5.1.4 Other Therapeutics Administered as Part of the MHP: Fibrinogen Concentrate

Intervention group: Patients randomized to the intervention group will receive 4 g FC (*Fibryga*) in both the first MHP pack and in the second MHP pack if requested (see 5.1.1 for further details). If a third MHP pack is required, and thereafter, FC will be administered if the fibrinogen level drops below 1.5–2.0 g/L at the discretion of the clinical team (Table 5) or based on conventional laboratory test results (Appendix 5) or viscoelastic methods (Appendix 4).

Control group: Patients randomized to the control group may receive FC if the fibrinogen level drops below 1.5–2.0 g/L at the discretion of the clinical team (Table 5) or based on conventional laboratory test results (Appendix 5) or viscoelastic methods (Appendix 4). FC dosing in MHP packs 3 and above will be site-specific and at the discretion of the treating clinician.

Table 5 Fibrinogen Concentrate as Part of the MHP (First and Second Packs) at Each Study Site

Participating site	Targeted plasma fibrinogen level (g/L)	Fibrinogen concentrate dose
Sunnybrook Health Sciences Centre	>2.0	4 g
Saint Michael's Hospital	>1.5–2	4 g
Montreal General Hospital	At discretion of treating physician	4 g
Foothills Medical Centre	>1.5–2	4 g
Vancouver General Hospital	>2.0	4 g
The Ottawa Hospital	At discretion of treating physician	4 g
Hamilton General Hospital	At discretion of treating physician	4 g
London Health Sciences Centre	>1.5	4 g

MHP = massive hemorrhage protocol.

5.1.5 Other Therapeutics Administered as Part of the MHP: Tranexamic acid

All patients will receive 1 g tranexamic acid within 3 hours of injury, with a further 1-g dose given based on the MHP at each study site (Table 6), unless already administered in the pre-hospital phase of care.

Table 6 Tranexamic Doses as Part of the MHP at Each Study Site

Participating site	Tranexamic acid dosing
Sunnybrook Health Sciences Centre	1 g + 1 g (both bolus)
Saint Michael's Hospital	1 g + 1 g (both bolus)
Montreal General Hospital	1 g + 1 g (both bolus)
Foothills Medical Centre	1 g IV bolus + 1 g IV over 8 h
Vancouver General Hospital	1 g + 1 g (mostly bolus, also infusion)
The Ottawa Hospital	1 g IV bolus + 1 g infusion over 8 h
Hamilton General Hospital	1 g upon MHP activation + additional 1 g at discretion of treating physician
London Health Sciences Centre	1 g over 10 minutes + 1 g infusion over 8 h

IV = intravenous; MHP = massive hemorrhage protocol.

5.2 Blinding, Emergency Envelopes, and Breaking the Study Blind

This is a single-blinded randomized study, with patients randomized to receive FC and PCC or to the current standard of care (RBCs and FP administered in a 1:1 ratio (see **Section 5.1**).

Given that the products have quite different physical differences, it is not possible to blind the treating clinicians to group assignment. Outcome assessors will remain blinded by using a generic product label in the patient chart and/or the electronic product name (i.e., FiiRST-2 MHP pack 1, parts 1 to 4 and pack 2, parts 1 to 4, rather than specifying type of product used).

Blinding of treatment will be performed by blood bank technologists. For the intervention group, FC and PCC will be placed in a tamper-sealed room temperature container and issued with the first set of RBCs and will be opened in the trauma bay/ED only immediately before transfusion. Similarly, the control group will receive the standard MHP pack 1 in a tamper-proof cooler along with the first set of RBCs, which will only be opened immediately before transfusion. Patients and outcome assessors will be blinded to the intervention by standard study blood labels for the patient chart.

The random allocation schedule will be prepared by a biostatistician not involved in the conduct of the trial (see **Section 9.3**). To minimize bias, neither the individual randomizing nor any of the health care providers will know which treatment will be assigned to a given patient when the MHP pack is ordered.

5.3 Treatment Compliance

5.3.1 Drug Dispensing and Accountability

All local hospital policies for storage, issuing and administration of factor concentrates will apply. A drug dispensing log and the inventory will be kept current by the Investigator, detailing the dates and quantities of FC and PCC dispensed to each patient. The inventory will be available to the monitor to verify drug accountability during the study. Any unused or partially used FC or PCC, including empty containers, will be accounted for. Any un-infused concentrate will be returned to the blood bank. The returned FC or PCC will be returned to regular inventory or disposed according to the hospital policies (after documentation by research personnel of all returns for wastage and circumstances).

5.3.2 Assessment of Treatment Compliance

MHP packs and other therapies administered in this study will be ordered and administered by the clinical team in the hospital and will not be dependent on patient compliance.

6 STUDY CONDUCT

All trauma patients at risk of massive hemorrhage will be the source of potential patients in the study. Patients will be randomized if the MHP is activated according to the MHP activation criteria at each study site. Once eligibility is confirmed by the technologists with the trauma team, the blood bank medical laboratory technologist will randomize the patient to one of two groups: the intervention group or the control group.

Patients will be treated according to their group allocation for the first and second MHP packs that are ordered, according to MHP criteria at each study site. The maximum time frame for administration of the second MHP pack (if required) is 24 hours from arrival at the trauma bay/ED or until MHP termination (whichever is earlier). Following the first two MHP packs, all patients will receive MHP packs as required, according to MHP guidelines at each participating site, or transition to a laboratory- or viscoelastic-guided transfusion as per the local guidelines of each study site once the MHP has been terminated.

Results of laboratory testing upon arrival at the trauma bay/ED will be collected whenever they are performed pre- and post-drug administration. Overall, laboratory tests are done on patient's arrival at the trauma bay/ED and frequently during the resuscitation process if the MHP is activated, usually hourly or after every 4-6 RBCs.

Patients will be followed for up to 28 days post-arrival at the trauma bay/ED. During this period, secondary outcomes and safety data will be recorded: and secondary outcomes will be recorded up to 28 days, transfusions and laboratory tests will be recorded up to Day 7 post-arrival, and all AEs and SAEs will be recorded up to Day 28. If the patient is discharged before completion of follow-up period, patients and/or family members will be contacted by telephone (with permission obtained during informed consent process) for a brief interview for the determination of status (dead/alive) and any AEs up to Day 28 following arrival. To minimize loss of follow-up, the research team will obtain contacts for 2 members of the patient's care team where available.

The flow chart of assessments by study visit is given on page 13.

6.1 Study Procedure

6.1.1 Visit 1: Upon arrival at the trauma bay/ED

If the MHP is activated, the blood bank technologist will confirm the following criteria:

- The approximate time of injury and the arrival time in the trauma bay/ED
- The patient's age
- The inclusion and exclusion criteria are met.

If the patient meets the inclusion criteria and has no exclusion criteria, the blood bank technologist will then randomize patients to FC + PCC or standard of care according to the randomization schedule and prepare and release the products

  -

6.1.2 Visit 2: 24 hours following arrival at the trauma bay/ED

- Baseline data:

  - Demographics
  - Medical history, including risk factors for thromboembolic events and comorbidities
  - Concomitant medications
- Date and time of arrival at the trauma bay/ED
- Injury data
  - Time of injury
  - Mechanism (blunt or penetrating)
  - Injury severity score (ISS)
- Deferred consent by SDM or patient if recovered
- Total and individual numbers of units and volumes of ABPs (RBCs + FP + platelets) transfused within 6 and 24 hours post arrival at the trauma bay/ED
- Total volume (in liters) of crystalloids and colloids transfused within the first 6 hours and 24 hours following arrival at the trauma bay/ED
- Rescue use of rFVIIa and FC within 24 hours following arrival at the trauma bay/ED
- Laboratory assessments collected at 24 hours following arrival at the trauma bay/ED, where available, including baseline measurements
  - Plasma fibrinogen levels
  - INR, PT and aPTT
  - Hemoglobin levels
  - Hematocrit levels
  - Platelets
  - Bilirubin
  - Creatinine
  - Calcium
  - Bicarbonate
  - Base deficit, pH and lactate
  - Ethanol levels
  - Liver enzymes (ALT, ALP, AST, albumin)
  - Thromboelastometry measurements collected at 24 hours following arrival at the trauma bay/ED, where available
    - EXTEM CT
    - EXTEM A10 and MCF
    - FIBTEM A10 and MCF
    - EXTEM LY30
- Thromboembolic events, including leg Doppler ultrasound or other imaging, as per clinical indications
- All AEs and SAEs, including:
  - MOF (SOFA score)

- ACS and LCS
- Transfusion-related reactions
- TEAEs

6.1.3 Visit 3: Days 2–27 following arrival at the trauma bay/ED

- Deferred consent by SDM or patient if recovered
- All data points that were not collected during visits 1 & 2
- Total and individual numbers of units and volumes of ABPs (RBCs + FP + platelets) transfused within 7 days following arrival at the trauma bay/ED
- Laboratory assessments, where recorded or measured within 7 days following arrival at the trauma bay/ED
    - Plasma fibrinogen levels
    - INR and aPTT
    - Hemoglobin levels
    - Hematocrit levels
    - Platelets
    - Bilirubin
    - Creatinine
    - Calcium
    - Bicarbonate
    - Base deficit, pH and lactate
    - Ethanol levels
    - Liver enzymes (ALT, ALP, AST, albumin)
    - Thromboelastometry measurements collected at 7 days following arrival at the trauma bay/ED, where available
        - EXTEM CT
        - EXTEM A10 and MCF
        - FIBTEM A10 and MCF
        - EXTEM LY30
- Thromboembolic events, including leg Doppler ultrasound or other imaging, as per clinical indications
- All AEs and SAEs, including:
    - MOF (SOFA score, recorded daily for patients still in the ICU)
    - ACS and LCS
    - Thromboembolic events
    - Transfusion reactions
    - TEAEs

6.1.4 Visit 4: Day 28 post-arrival at the trauma bay/ED (in person if in hospital or by phone)

- Deferred consent by SDM or patient if recovered
- All data points that were not collected during visits 1, 2 & 3
- Thromboembolic events, including leg Doppler ultrasound or other imaging, as per clinical indications
- Ventilator-free days
- Days out of hospital within the 28 days follow-up
- Time to death
- All AEs and SAEs, including:
  - MOF (SOFA score) for patients still in the ICU
  - ACS and LCS
  - Transfusion reactions
  - TEAEs
- Number of days in the ICU
- 28-day all-cause mortality
  - Cause of death (adjudicated by an independent physician and one of the Investigators blinded to treatment allocation):
    - Time of death
    - Time for referral for brain death determination
    - Time of brain death
    - Neurological due to traumatic brain injury
      - Record time of annotation of possible brain death
      - Record time to referral to neurology
      - Official brain death declaration time
    - MOF/sepsis
    - Other, with specific cause detailed

After Day 28 following arrival at the trauma bay/ED, the clinical study is considered completed for all patients. No further study-related assessments will be performed, unless safety concerns (e.g., ongoing AEs) require follow-up.

6.1.5 Maximum Visit Time Frame Used in this Study

In this study, the following maximum time frames for each visit will apply:

Table 7 Study Visits with Accepted Time Range

Visits	Time point	Accepted time range
**Visit 1**	Upon arrival at the trauma bay/ED (for screening and randomization). Performed by the blood bank technologist in	60 minutes ± 10 minutes

	contact with the trauma team leader, both present in the hospital 24 hours per day	
**Visit 2**	At 24 hours from arrival at the trauma bay/ED (for deferred consent, primary and secondary endpoints, and other data collection). Performed by the research assistant, present in the hospital during working hours, Mondays to Fridays	24 hours ± 4 hours
**Visit 3**	Day 2 to Day 27 from arrival at the trauma bay/ED (for deferred consent and secondary endpoints). Performed by the research assistant, present in the hospital during working hours, Mondays to Fridays	48 hours to 27 days inclusive
**Visit 4**	Day 28 (deferred consent, safety endpoints and 28-day mortality). Performed by the research assistant, present in the hospital during working hours, Mondays to Fridays	28 days ± 3 days

ED = emergency department.

6.2 Duration of Study

6.2.1 Planned Duration for an Individual Patient

The **duration of the treatment period** is from randomization following activation of the MHP, until the second MHP pack has been administered (or before if the patient does not receive the second pack or the MHP is terminated). The maximum time frame for administration of the second MHP pack is 24 hours from arrival at the trauma bay/ED or until the MHP is terminated, whichever occurs first. Should administration of units from the second pack occur prior to 24 hours post trauma bay/ED arrival and the infusion extend beyond 24 hours, all ABP units transfused from the pack will be included in the primary endpoint analysis.

The **duration of the study** for an individual patient is 28 days from patient arrival when patients will be contacted in person or by phone post-discharge.

6.2.2 Planned Duration for the Study as a Whole

Recruitment will be closed if the expected 350 patients are recruited (with the number of enrolled patients adjusted after the unblinded interim analysis performed once 120 patients have completed the study). The total duration of this trial will be no more than 36 months.

6.2.3 Premature Termination of the Study

Both the Investigator and the Sponsor, in consultation with the IDSMC, reserve the right to terminate the study at any time. In this event, any necessary procedures will be arranged on an individual study basis after review and consultation by both parties. In terminating the study, the Investigators will ensure that adequate consideration is given to the protection of the patients’ interests.

Regulatory authorities and research ethics boards (REBs) will be informed in accordance with national regulations.

Early termination of the study as a whole or by center may apply for the following reasons:

Early Termination of the Entire Clinical Study

At any time, the study as a whole will be terminated prematurely if:

New toxicological or pharmacological findings or safety reports invalidate the earlier positive benefit-risk-assessment.

Early Termination at an Individual Study Center

At any time, the study can be terminated at an individual center if:

- The center cannot comply with the requirements of the protocol
- The center cannot comply with GCP standards
- The a priori determined required recruitment rate is not met

7 ASSESSMENTS AND METHODS

7.1 Baseline Data

The baseline information and medical history will be recorded after Visit 1, i.e., as soon as possible following randomization and consent.

7.1.1 Demographic and Baseline Characteristics

The demographic and baseline characteristics are sex, age, height, weight, and Body Mass Index (BMI).

7.1.2 Medical History and Prior/Concomitant Medications

The medical history will be obtained by interviewing the patient or from the medical records.

Prior (up to 7 days before arrival) and concomitant medications will be obtained.

7.2 Efficacy Assessments

7.2.1 Transfusion Data

All blood products and hemostatic agents released from the blood bank and transfused will be collected from the blood bank information systems. These include ABPs: RBCs, pooled or apheresis platelets, and plasma. Other hemostatic agents include tranexamic acid, FC, PCC and rFVIIa. Transfusion of crystalloids and colloids will also be assessed. For the purposes of the study, a unit will refer to 1 unit of RBCs or 1 unit of plasma. One dose of platelets will be defined as 4 U of pooled or single donor platelets, for consistency.

7.3 Laboratory Assessments

7.3.1 Test Parameters and Laboratories

Table 8 summarizes all test parameters and the laboratories responsible for analysis.

Table 8 Test Parameters and Laboratories

Test	Material needed	Responsible laboratory
**Coagulation profile**		
Fibrinogen activity via Clauss method	Citrated blood	Local
INR, aPTT	Citrated blood	Local
ROTEM EXTEM CT, EXTEM A10, EXTEM MCF, FIBTEM A10, FIBTEM MCF, and EXTEM LY30	Citrated blood	Local, if available
**Hematology**		
Hemoglobin levels	Citrated blood	Local
**Safety labs**		
Liver enzymes (ALT, ALP, Bilirubin, albumin)	Serum	Local
Troponin	Serum	Local
Bicarbonate	Serum	Local
Lactate	Serum	Local
Base deficit	Serum	Local
Ethanol levels	Serum	Local

A10 = amplitude at 10 minutes; ALP = alkaline phosphatase; ALT = alanine aminotransferase; CFT = clot formation time; CT = clotting time; INR = international normalized ratio; MCF = maximum clot firmness; LY30 = lysis at 30 minutes; ML = maximum lysis.

7.3.2 Blood Sampling

All blood sampling will be performed as per standard practice at the local institution. Where required for test interpretation, test methodology will be recorded.

The *actual* time of blood sampling will be recorded in the CRF and on the corresponding laboratory requisition forms and/or electronic system.

7.3.3 Citrated Blood

Citrated blood as required by the local laboratory will be collected and processed in accordance with local requirements.

7.3.4 Serum

For the determination of clinical chemistry and safety labs, where a serum blood sample has been collected.

7.3.5 Recording of Clinically Significant Abnormal Laboratory Values as AEs/ADRs

Other than abnormal laboratory values due to pre-existing underlying conditions (e.g., patients in chronic renal failure will have an elevated creatinine), the Investigator must assess the clinical significance of abnormal laboratory values outside the specified normal range (see **Section 7.4**). Any clinically significant abnormalities will be documented. All specified clinically significant laboratory abnormalities requiring intervention or medical treatments will be documented as AEs/SAEs and investigated.

Additional tests and other evaluations required to establish the significance or etiology of specified abnormalities or to monitor the course of an AE will be obtained if clinically indicated. Follow-up will persist until resolution or up to the Study Completion Visit, whichever occurs first.

7.4 Safety Assessments

7.4.1 Assessments for Safety Endpoints

The following drug safety information will be collected:

- Incidence of thromboembolic events (DVT, PE, MI, ischemic stroke, and arterial or venous thrombosis at other sites).
- AEs and SAEs temporally associated with the administration of IMP (for definitions and reporting requirements, see **Sections 7.4.2, 7.4.3,** and **7.4.4**), including:
    - Incidence and severity of MOF
    - Incidence of ACS and LCS
    - Incidence of transfusion reactions
    - Incidence of TEAEs.
- Pregnancies, drug overdose, interaction, medication error, lack of efficacy, and post-study SAEs (see **Section 7.4.9**).

7.4.2 Adverse Events

Definitions

AEs will be coded according to the latest Medical Dictionary for Regulatory Activities (MedDRA) version as specified in the Data Management Plan. The analysis will focus on TEAEs, i.e., AEs that started or worsened after start of infusion with IMP.

Adverse event (AE): An AE is any untoward medical occurrence in a study patient receiving an IMP and which does not necessarily have a causal relationship with this treatment. An AE can therefore be any unfavorable and unintended sign (including an abnormal laboratory finding), symptom, or disease temporally associated with the use of an IMP, whether or not related to the IMP.

Adverse drug reaction (ADR): An ADR is any noxious and unintended response to an IMP related to any dose. The phrase ‘response to an IMP’ means that a causal relationship between the IMP and an AE carries at least a reasonable possibility, i.e., the relationship cannot be ruled out.

Other significant AEs: Any marked laboratory abnormalities or any AEs that lead to an intervention, including withdrawal of drug treatment, dose reduction, or significant additional concomitant therapy.

Withdrawal due to AE/ADR: AE/ADR leading to discontinuation of treatment with IMP. Any such events will be followed up by the Investigator until the event is resolved or until the medical condition of the patient is stable. All follow-up information collected will be made available to the Principal Investigator (Sponsor).

Collection of AEs

The condition of the patient will be monitored throughout the study. At each visit, whether scheduled or unscheduled, AEs will be elicited using a standard non-leading question such as "How have you been since the last visit/during the previous study period?" In addition, the Investigator will check the patient records for any documented event.

Any AE or ADR which occurs during the study will be noted in detail on the appropriate pages of the CRF. If the patient reports several signs or symptoms representing a single syndrome or diagnosis, the diagnosis should be recorded in the CRF. The Investigator will grade the severity of all AEs or ADRs (mild, moderate, or severe), the seriousness (non-serious or serious), and the likelihood that they were related to the IMP (causality). The investigator will also assess the expectedness of each ADR (expected or unexpected).

Diseases, signs and symptoms, and/or laboratory abnormalities already present before the first administration of IMP will not be considered AEs unless an exacerbation in intensity or frequency (worsening) occurs.

The Investigator will provide detailed information about any abnormalities and about the nature of and reasons for any action taken as well as any other observations or comments that may be useful for the interpretation and understanding of an AE or ADR.

Severity of AEs

The intensity/severity of AEs will be graded as follows:

Mild: an AE, usually transient, which causes discomfort but does not interfere with the patient's routine activities

Moderate: an AE which is sufficiently discomforting to interfere with the patient's routine activities

Severe: an AE which is incapacitating and prevents the pursuit of the patient's routine activities

The grading of an AE is up to the medical judgment of the Investigator and will be decided on a case-by-case basis.

Causality of AEs

The relationship of AEs to the administered IMP will be assessed by the Investigator:

Probable: Reports including good reasons and sufficient documentation to assume a causal relationship, in the sense of plausible, conceivable, likely, but not necessarily highly probable. A reaction that follows a reasonable temporal sequence from administration of the IMP; or that follows a known or expected response pattern to the suspected medicine; or that is confirmed by stopping or reducing the dosage of the medicine and that could not reasonably be explained by known characteristics of the patient's clinical state.

Possible: Reports containing sufficient information to accept the possibility of a causal relationship, in the sense of not impossible and not unlikely, although the connection is uncertain or doubtful, for example because of missing data or insufficient evidence. A reaction that follows a reasonable temporal sequence from administration of the IMP, following a known or expected response pattern to the suspected medicine, but that could readily have been produced by a number of other factors.

Unlikely: reports not following a reasonable temporal sequence from IMP administration. An event which may have been produced by the patient's clinical state or by environmental factors or other therapies administered.

Not related (unrelated): events for which sufficient information exists to conclude that the etiology is unrelated to the IMP.

Unclassified: reports which for one reason or another are not yet assessable, e.g., because of outstanding information (can only be a temporary assessment).

Classification of ADRs by Expectedness

ADRs will be classified by the Sponsor as either expected or unexpected:

Expected: an ADR that is listed in the current edition of the Investigator's Brochure (IB) or other reference safety information.

Unexpected: an ADR that is not listed in the current edition of the IB or other reference safety information, or that differs because of greater severity or greater specificity.

Outcome of AEs

The outcome of all reported AEs has to be documented as follows:

1. Recovered, resolved
2. Recovering, resolving
3. Not recovered, not resolved (by Study Completion visit)
4. Recovered, resolved with sequelae
5. Fatal
6. Unknown

NOTE: A patient's **death** per se is not an event, but an outcome. The event which resulted in the patient's death will be fully documented and reported.

Action(s) taken

AEs requiring action or therapy must be treated with recognized standards of medical care to protect the health and well-being of the patient. Appropriate resuscitation equipment and medicines must be available to ensure the best possible treatment in an emergency situation.

The action taken by the Investigator must be documented:

a) General actions taken in the event of an AE

- None
- Medication (other than IMP) or other (e.g., physical) therapy started
- Test performed
- Other (to be specified)

b) IMP-related actions taken in the event of an AE

- None
- Product withdrawn

– Dose reduced
– Dose increased

The Investigator will follow up on each AE until it has resolved or until the medical condition of the patient has stabilized. Any relevant follow-up information will be reported to the Principal Investigator (Sponsor).

7.4.3 Serious Adverse Events

An **SAE** is any untoward medical occurrence that at any dose:

- Results in death,
- Is life-threatening (see below),
- Requires hospitalization or prolongation of existing hospitalization,
- Results in persistent or significant disability/incapacity,
- Is another important medical event.

NOTES:

The term 'life-threatening' refers to an event in which the patient was, in the view of the reporting Investigator, at immediate risk of death at the time of the event; it does not refer to an event which may hypothetically have caused death had it been more severe.

The term 'disability' is defined as a substantial disruption of a person's ability to conduct normal life functions.

An important medical event is an event that may not result in death, be life-threatening, or require hospitalization but may be considered an SAE when, based upon appropriate medical judgment, it may jeopardize the patient and may require medical or surgical intervention to prevent one of the outcomes listed in the definitions for SAEs. Examples of such medical events include severe myocardial infraction requiring intensive treatment in an emergency or operating room.

In deciding whether an AE/ADR is serious, medical judgment should be exercised. Thus, important AEs/ADRs that are not immediately life-threatening or do not result in death or hospitalization but may jeopardize the patient or may require intervention to prevent one of the other outcomes listed in the definitions above should also be considered serious.

In addition, although not classified under the seriousness criteria, all suspected transmissions of an infectious agent should be reported as an SAE. A suspected virus transmission means that virus antigen has been detected in the patient. A passive transmission of antibodies alone does not constitute a suspected virus transmission.

7.4.4 SAE Reporting Timelines

All AEs and SAEs that are possibly (or greater imputability) related to the study intervention that occur after the study intervention through 28 days follow-up period must be promptly reported to the REB. A suspected unexpected serious adverse reaction (SUSAR) is an unexpected serious adverse event that is related to study drug. The Investigator or designee is responsible for reporting SAEs and/or SUSARs to relevant regulatory authorities and REB per local reporting requirements. In addition, SAEs will be reviewed at each IDSMC meeting.

<u>Reportable SAEs</u>

Reportable SAEs will be reported to local hospital REB.

All SAEs, suspected to be related to study treatment, will be reported within 24 hours of recognition to the sponsor by telephone, fax, or email:

Keyvan Karkouti MD
Department of Anesthesia
Toronto General Hospital
200 Elizabeth Street, 3EN
Toronto, ON
M5G 2C4
Canada

Phone: 1-416-340-5164
Fax: 1-416-340-3698
Email: keyvan.karkouti@uhn.ca

In addition, all serious adverse events related to Fibryga or Octaplex will be reported within 1-month of recognition of the event to:

Octapharma's Corporate Drug Safety Unit
OCTAPHARMA Pharmazeutika Produktionsges.m.b.H.
Oberlaaer Strasse 235, 1100 Vienna, Austria
Fax: +43 1 61032-9949
Email: cdsu@octapharma.com

24 hours emergency telephone number: +43 1 40 80 500

Waivers from the SAE Reporting Requirement

Waivers from the SAE reporting requirement include surgeries that are elective or were planned before study entry or prolongations of existing hospitalizations for economic or social, but not medical, reasons. Such surgeries or prolongations of hospitalizations should not be considered SAEs.

7.4.5 Incidence of thromboembolic events

As a component of the assessment of AEs, the Investigator will record the occurrence of thromboembolic events, as defined by evidence of any of the following:

<u>Deep Venous Thrombosis [97]</u>

DVT will be defined as presence of an intraluminal filling defect in a vessel (vein or artery) in any of the following:

- A persistent intraluminal filling defect on contrast venography;
- Non-compressibility of one or more venous segments on B mode compression ultrasonography;

A clearly defined intraluminal filling defect on contrast enhanced computed tomography.

Pulmonary Embolism [97]

PE will be defined as mismatch at ventilation/perfusion scintigraphy or demonstration of a thrombus by computed tomography, pulmonary angiography/venography or magnetic resonance pulmonary angiography/venography. The diagnosis of PE requires any one of the following:

- A high probability ventilation/perfusion lung scan
- An intraluminal filling defect of segmental or larger artery on a helical computed tomography scan
- An intraluminal filling defect on pulmonary angiography
- A positive diagnostic test for femoral vein thrombosis (e.g., positive compression ultrasound) and one of the following:
    - Non-diagnostic (i.e., low or intermediate probability) ventilation/perfusion lung scan
    - Non-diagnostic (i.e., subsegmental defects or technically inadequate study) helical computed tomography scan.

Myocardial Infarction

MI will be defined according to the Executive Group on behalf of the Joint European Society of Cardiology, American College of Cardiology, American Heart Association, and World Heart Federation Task Force for the Universal Definition of Myocardial Infarction [1].

Ischemic Stroke

Stroke will be defined as an infarction of the central nervous system, based on neuropathological, neuroimaging, and/or clinical evidence of permanent injury. When imaging or pathology is not available, clinical stroke is recognized by persistence of symptoms for 24 hours [2]. A transient ischemic attack is defined as the occurrence of focal neurological symptoms or signs that last <24 hours [2]. Diagnosis is clinical.

Arterial or Venous Thrombosis at Other Sites

Arterial or venous thrombosis at other sites will be recorded, as diagnosed by the clinical team.

Thromboembolic events will be captured on a specific page of the CRF. All incidences of thromboembolic events must also be recorded on the AE page of the CRF.

7.4.6 Incidence and severity of MOF

As a component of the assessment of AEs, the Investigator will record the occurrence of MOF, as measured by the Sequential Organ Failure Assessment score (SOFA) [98] daily following arrival at the trauma bay/ED, while the patient is still in the ICU. The highest SOFA score, time of highest SOFA score and change in SOFA score will be assessed.

Organ dysfunction will be identified as an acute change in total SOFA score ≥2 points consequent to infection. The baseline SOFA score will be assumed to be zero in patients not known to have pre-existing organ dysfunction. MOF will be captured on a specific page of the CRF. All incidences of MOF must also be recorded on the AE page of the CRF. The diagnosis will be clinical, performed by the critical care physician, and based on the definitions described in Table 9 below.

Table 9 The Sequential Organ Failure Assessment Score (SOFA) [4]

	SOFA score				
	**0**	**1**	**2**	**3**	**4**
Respiration					
PaO_2 / FIO_2, mmHg	>400	≤400	≤300	≤200 With respiratory support	≤100 With respiratory support
Coagulation					
Platelets, $\times 10^3/\mu L$	>150	≤150	≤100	≤50	≤20
Liver					
Bilirubin, mg/dL	<1.2	1.2–1.9	2.0–5.9	6.0–11.9	>12.0
Bilirubin, µmol/L	<20	20–32	33–101	102–204	>204
Cardiovascular					
Hypotension	No hypotension	MAP <70 mm Hg	Dopamine ≤5 or dobutamine (any dose)	Dopamine >5 or epinephrine ≤0.1 or nor epinephrine = 0.1	Dopamine >15 or epinephrine >0.1 or nor epinephrine >0.1
Central Nervous System					
Glasgow Coma Score*	15	13–14	10–12	6–9	<6
Renal					
Creatinine, mg/dL (µmol/L)	<1.2	1.2–1.9	2.0–3.4	3.5–4.9	>5.0
or urine output, mL/day	<110	110–170	171–299	300–440 or <500	>440 or <200

FIO_2 = fraction of inspired oxygen; MAP = mean arterial pressure; PaO_2 = partial pressure of arterial oxygen

7.4.7 Incidence of abdominal compartment syndrome and limb compartment syndrome

As a component of the assessment of AEs, the Investigator will record the occurrence of abdominal compartment syndrome (ACS) and limb compartment syndrome (LCS). The diagnosis of both ACS and LCS will be performed by the clinician.

Diagnosis of Abdominal Compartment Syndrome

For ACS intra-abdominal pressure will be recorded, along with any required intervention and surgeries performed for treatment. A diagnosis of ACS will be defined as sustained intra-abdominal pressure >20 mmHg with or without an abdominal perfusion pressure (APP) of <60 mmHg, that is associated with new organ dysfunction/failure [5].

Diagnosis of Limb Compartment Syndrome in Alert Patients

In alert patients with limb injuries, LCS should be diagnosed in the presence of clinical signs of the condition, as described in Table 10.

Table 10 Diagnosis of Limb Compartment Syndrome in the Alert Patient, as per Wall et al. [6]

Clinical signs of acute limb compartment syndrome
1. Palpable tenseness or swelling of the compartment
2. Pain out of proportion to the injury
3. Pain on passive stretch of muscles within the compartment
4. Paresthesia of skin supplied by nerves traversing the compartment
5. Paresis of muscles supplied by nerves traversing the compartment
6. Pallor of skin overlying the compartment
7. Pulses present

In alert, high-risk patients with equivocal clinical findings, compartment pressure should be measured. In addition, in the initial absence of clinical signs, alert, high-risk patients should be assessed for clinical signs of LCS at least every 4 hours for a minimum of 24 hours after the precipitating injury.

In alert patients, it is recommended that LCS be diagnosed upon demonstration of clinical signs of the condition. The seven clinical signs listed in Table 10 may be present in varying combinations and severity. Clinical diagnosis of LCS is not always straightforward – a degree of swelling is expected following any injury, pain is difficult to interpret as a clinical sign because of its subjectivity, and paresthesia and paresis are considered late signs.

Considering this, it is imperative that the treating doctor performs a thorough examination and carefully interprets any observed signs. A high index of suspicion is paramount in the diagnosis of LCS. Although a present distal pulse is not actually a clinical sign, it is included in the clinical practice guidelines to ensure that clinicians do not wait until the absence of pulses before making the diagnosis. If the diagnosis is uncertain but definite exclusion of LCS is not possible with clinical examination, it is recommended that high-risk patients undergo compartment pressure measurement.

Diagnosis of Limb Compartment Syndrome in Unconscious Patients

In unconscious patients with limb injuries, LCS should be diagnosed in the presence of raised compartment pressure. In the initial absence of raised pressure, unconscious, high-risk patients should have their compartment pressure measured at least every 4 hours for a minimum of 24 hours after the precipitating injury.

Both abdominal and limb compartment syndrome will be captured on a specific page of the CRF. All incidences of abdominal and limb compartment syndrome must also be recorded on the AE page of the CRF. The diagnoses of both ACS and LCS will be confirmed by the critical care team responsible for the patient, in conjunction with the trauma surgeon, orthopedic surgeon or vascular surgeon.

7.4.8 Incidence of transfusion reactions

As a component of the assessment of AEs, the Investigator will record the occurrence of transfusion reactions, as defined by the International Society of Blood Transfusion [7].

7.4.9 Other Relevant Safety Information

Pregnancies

Patients who are known to be pregnant will not be included in the study.

Overdose, Interaction, Medication Error and Lack of Efficacy

The following safety relevant information should be reported as an AE or, if the reaction fulfills one of the criteria for seriousness, as an SAE.

a) Drug overdose

An overdose is a deliberate or inadvertent administration of a treatment at a dose higher than specified in the protocol and higher than the known therapeutic dose that is of clinical relevance. The reaction must be clearly identified as an overdose.

b) Drug interaction

A drug interaction is a situation in which a substance or medicinal product affects the activity of an IMP, i.e., increases or decreases its effects, or produces an effect that none of the products would exhibit on its own. The reaction must be clearly identified as a drug interaction.

c) Medication error

A medication error involves the inadvertent administration or unintended use of a medicinal product which may be caused by the naming, presentation of pharmaceutical form/packaging, or instructions for use/labeling. The reaction must be clearly identified as a medication error.

8 DATA HANDLING AND RECORD KEEPING

8.1 Documentation of Data

8.1.1 Source Data and Records

Source data are defined as all information related to clinical findings, observations, or other activities in the study, written down in original records or certified copies of original records, allowing reconstruction and evaluation of the clinical study.

The Investigator will maintain adequate source records (e.g., case histories or patient files for each patient enrolled). Source records should be preserved for the maximum period of time required by local regulations.

For each patient enrolled, the Investigator will indicate in the source record(s) that the patient participates in this study.

All data entered in the electronic CRF (eCRF) must be supported by source data in the patient records, with exceptions listed in **Section 8.1.2**.

The Investigator will permit study-related monitoring, audit(s), REB review(s), and regulatory inspection(s), by providing direct access to the source data/records.

The Investigator will identify any patient for whom deferred informed consent was not obtained and request the deletion of any of their data (with the exception of the situations listed in Section 10.3.1) from the study database. Execution of such deletions will be documented on a separate form.

The Investigator may authorize site staff (e.g., sub-investigators, clinical research coordinators/assistants, nurses) to enter study data into the eCRF. This must be documented in the Delegation of Duties Log signed by the Investigator.

8.1.2 Case Report Forms

Study site staff (e.g., blood bank technologist, research coordinator/assistant) will be responsible for completing a CRF for each patient enrolled. All site personnel will be trained on CRF completion. The site is also provided with the approved CRF Completion Guidelines which will assist in data entry and data issues/questions. Additional site training may be provided as refreshers throughout the study, if needed. All persons allowed to enter or to change CRF data must be listed in the Delegation of Duties Log.

For each patient enrolled, an eCRF will be completed within the Electronic Data Capture (EDC) system and approved by the Investigator or an authorized sub-investigator.

Study site staff will be responsible for entering patient data into the validated EDC system. All site personnel will be trained on the EDC system and study specific eCRFs prior to receiving access to the live database for data entry.

8.1.3 Changes to Case Report Form (CRF) Data

Monitors will perform source data verification (SDV) as defined for the study.

If any errors or discrepancies in the eCRFs are found during data entry or review, discrepancies will be generated programmatically within the EDC system, and 'manual' queries will be generated by either a monitor or Data Management.

Discrepancies and queries can only be corrected by the Investigator(s) or other authorized site personnel. An audit trail documents all changes to the data over the entire study period. If the reason for a change is not obvious, a comment must be supplied in the query's response, stating the reason for the change, prior to closing. The study monitor should provide guidance to Investigator(s) and the Investigator(s)' designated representatives on making such corrections.

Once queries have been resolved by the site staff, the resolutions are assessed by Data Management. If the query response provided confirms the data as correct, the discrepancy will be closed. If the response does not adequately address the question raised, a new query will be issued for further clarification.

Manual checks are performed and programs are run throughout the study until the data is clean and the database is ready for lock. All discrepancies will be resolved prior to database lock. There will be a final run of the programmed checks to ensure all discrepancies are closed out, SDV will be confirmed as complete by the monitor, and all eCRFs will be approved by the Investigator prior to database lock.

8.2 Information to Investigators

An IB will be handed out to the Investigator before the start of the study. The IB contains all information in the Sponsor's possession necessary for the Investigator to be fully and accurately informed about the safety of *Fibryga* and *Octaplex*.

The IB will be updated at regular intervals by Octapharma and whenever relevant new information concerning the IMPs becomes available. This will be delivered by Octapharma to the Principal Investigator who will distribute to the approved study sites.

The Investigator will be informed about the methods for rating relevant study outcomes and for completing CRFs to reduce discrepancies between participating Investigator and study sites.

The Investigator will be kept informed of important data that relate to the safe use of the IMPs as the study proceeds.

8.3 Responsibilities

At each study site the Investigator is accountable for the conduct of the clinical study. Responsibilities may be delegated to appropriately qualified persons.

A Delegation of Duties Log will be filled in and signed by the Investigator. In accordance with this delegation log, study site staff (e.g., sub-investigators, nurses) are authorized to perform tasks relating to the study.

8.4 Investigator's Site File

At each study site, the Investigator is responsible for maintaining all records to enable the conduct of the study to be fully documented. Essential documents as required by GCP guidelines and regulations (e.g., copies of

the protocol, study approval letters, all original informed consent forms, site copies of all CRFs, drug dispensing and accountability logs, correspondence pertaining to the study, etc.) should be filed accurately and kept by the Investigator for the maximum period of time required by local regulations.

The Investigator is responsible for maintaining a confidential patient identification code list, which provides the unique link between named source records and CRF data for the Sponsor. The Investigator must arrange for the retention of this confidential list for the maximum period of time required by local regulations.

No study document should be destroyed without prior written agreement between the Investigator and the Sponsor. Should the Investigator elect to assign the study documents to another party, or move them to another location, the Sponsor must be notified in writing.

8.5 Provision of Additional Information

On request, the site investigators will supply the Sponsor or designate, such as the monitors with additional data relating to the study, or copies of relevant source records, ensuring that the patient's confidentiality is maintained. This is particularly important when CRFs are illegible or when errors in data transcription are encountered. In case of particular issues or governmental queries, it is also necessary to have access to the complete study records, provided that the patient's confidentiality is protected in accordance with applicable regulations.

8.6 Independent Data Safety Monitoring Committee

An IDSMC will be established by the Sponsor. The IDSMC will be composed of recognized experts in the field of emergency medicine, critical care medicine, methodology/epidemiology, and anesthesiology. To identify any safety concerns with the conduct of the trial, the IDSMC will review study outcomes after 60 patients have been enrolled, at the interim and then every 100 patients thereafter. In particular, data on deaths, SAEs and thromboembolic events will be reviewed in detail. The IDSMC will review data at the adaptive interim analysis after 120 patients have completed the study, at which point it will give advice on the continuation, modification, or termination of the study. A written study-specific charter will define in detail the composition, responsibilities, and procedures of the IDSMC. The IDSMC chair will be notified of every SAE thought to be related to the study medication. A written study-specific charter will define in detail the composition, responsibilities, and procedures of the IDSMC.

Reports from the IDSMC meetings will be submitted to the REB.

9 STATISTICAL METHODS AND SAMPLE SIZE

The statistical analysis will be delegated under an agreement of transfer of responsibilities to an external biostatistician. The principal statistical methodology is described in this section. Further specifics regarding the statistical analysis will be provided in the Statistical Analysis Plan (SAP).

9.1 Determination of Sample Size

The statistical analysis of the primary outcome, a composite number of units of ABPs (RBC + FP + platelets) transfused within 24 hours post trauma bay/ED arrival, will be based on the mean number of ABPs within the first 24 hours. To demonstrate that the early administration of FC + PCC is clinically superior to the usual component therapy (FC administration depending on fibrinogen levels and/or clinician discretion and clotting factor replacement with FP), with respect to the mean number of ABPs, a two-sample, one-sided test of the pair of hypotheses: H_0: $RR \geq RR_0$ vs. Ha: $RR < RR_0$ will be carried out with an overall type I error probability of $\alpha = 0.025$. Here, λ_1 and λ_2 denote the mean number of ABPs (RBC + FP + platelets) in the control group (Standard of Care; SoC) and intervention groups, respectively, RR is the ratio λ_2/λ_1 and RR_0 will be set equal to 1.0 to test for superiority. A mean difference in 5 units of the composite outcome (mean 15 units in the control group and mean 10 units in the intervention group) is considered as a clinically meaningful difference that should be detected with at least 80% power.

Testing of the hypothesis will be performed in the context of a counting regression model (generalized linear model for count data with log-link function and a negative binomial error term), with treatment group as main effect. Inferences will be based on the one-sided 97.5% confidence interval (CI) for the ratio λ_2 / λ_1 derived from the estimated least square means (LSmeans) of this model. Superiority will be concluded if the upper limit of this CI is strictly less than $R_0 = 1.0$ (i.e. the mean number of ABPs is larger in the SoC group). Sample size estimations based on these assumptions were performed with the software nQuery (version 8.3). Empirical estimates of the mean number of allogeneic blood products units within the first 24 hours and its dispersion were based on results of the FIIRST-1 Study [49] with the same endpoint in the same indication and similar treatment. The Table 11 displays the sample sizes per treatment arm and different assumptions about λ_1, λ_2 and a common dispersion of 1.0. Among the different scenarios, Scenario no. 3 appeared to be most probable. Therefore, a net sample size of 297 patients would suffice to demonstrate the superiority of the investigational treatment under the stated assumptions. The FIIRST-1 study had a 10% patient drop-off (exclusions post randomization). Hence, we will inflate our sample size to account for a drop-out percentage of up to 15%. For this reason, the study plans to enroll up to 350 patients.

Table 11 Sample Size Scenarios.

Scenario	1	2	3	4
RR_0	1.0	1.0	1.0	1.2
λ2	12	11	10	9
λ1	15	15	15	15
RR = λ2/λ1	0.8	0.733	0.667	0.600
Power	80%	80%	80%	80%
Dispersion	1.0	1.0	1.0	1.0
Total Sample Size	1001	506	297	188

λ1 = mean number of ABPs (RBCs + FP + platelets) in the control group; λ2 = mean number of ABPs (RBCs + FP + platelets) in the intervention group.
ABP = allogenic blood product; FP = frozen plasma; RBCs = red blood cells; RR = ratio of event rates (λ_2/λ_1); RR_0 = ratio of event rates under the null hypothesis (set to 1.0 to test for superiority).

9.2 Statistical Analysis

For the statistical analysis the following analysis sets will be considered:

The intention to treat (ITT) population: All randomized patients who receive at least a whole or parts of their first randomized MHP pack (not including the RBCs) and agree to remain in the study after consenting. Should a patient receive MHP packs that are not in concordance with the randomization schedule, the treatment group will be defined according to the randomization (rather than the actual treatment received). The primary outcome and mortality for non-consenting patients will be collected from as many sites as possible, as permitted by the local REB.

The modified intention to treat (mITT) population: All randomized patients who receive any of the non-RBC products in MHP pack 1 or beyond of the intended first-line treatment and agree to remain in the study after consenting. Should a patient receive treatment that is not in concordance with the randomization schedule, the treatment group will be defined according to the actual treatment received (rather than the randomization). If no randomization errors are observed the ITT population will be identical to the mITT population.

For both ITT and mITT populations the treatment assignment will be based on the first MHP pack, even if the second MHP pack should be from the opposing treatment group.

The per-protocol (PP) population: All patients in the ITT population, excluding patients with major protocol deviations. Patients meeting any of the following criteria will be excluded:

- Patients who, according to their assigned treatment arm, do not receive at least 2 g of FC AND 1000 IU of PCC after randomization or 2 units of FP
- Patients who significantly violate inclusion/exclusion criteria (e.g., not a trauma patient, randomized after 3 hours from injury, etc.)
- Patients receiving treatment not in concordance with the randomization schedule

The restricted safety population (SAF-REB): All patients who receive at least one whole or parts of their first randomized MHP pack, who (or their SDM) did not consent participation post randomization; but for whom REB approval is available for SAE data analysis for patients who had SAEs. Only SAE terms (including mortality) will be displayed for this restricted population.

A final decision about the classification of protocol deviations as major and minor and their consequences regarding assignment of patients to analysis populations will be made during the blinded data review meeting prior to unblinding for the interim and final analyses by the lead PI.

The mITT analysis population is considered the primary population for analysis of the primary endpoint. The evaluation of the primary endpoint will additionally be performed for the PP population.

The data will be analyzed using protocols established for safeguarding privacy and confidentiality. These include password protected computers, locked file cabinets for hardcopies, and no public discussion of individual cases. There will be no linkages to other datasets, and no disclosures of person identifier. Reports will present aggregate statistics only.

Summary data for continuous variables will be presented as means and standard deviations, or medians and interquartile ranges depending on the distribution. Discrete variables will be summarized as frequency and percentages. All-cause mortality and other event time will be analyzed by time-to-event methods (Kaplan-Meier curves, log-rank tests). Differences in binary outcomes will be assessed using exact tests for proportions. Likelihood ratio tests in the context of a generalized linear model (GLIM) for count data will be used to analyze ratios of allogeneic blood products. For categorical clinical endpoints, odds ratios (OR) and two-sided 95% confidence interval (CI) will be calculated.

Primarily in order to verify the assumptions on the primary endpoint and potentially adapt the sample size of the study an interim analysis will be performed after a total of 120 evaluable patients have completed the study. While the members of the IDSMC will be entitled to review unblinded interim results and base adaptation recommendations on the results, the blinding status for all other study team members will remain unchanged.

Additional details on the counting regression modeling, the adaptation rules after interim analysis, the group-sequential design and the specific statistical analyses for the study endpoints will be presented in a separate SAP.

9.2.1 Efficacy Analysis Plan

Primary Endpoint

The primary endpoint is to demonstrate superiority with respect to the composite number of ABPs (RBCs + FP + platelets) transfused within the first 24 hours following arrival at the trauma bay/ED. The statistical analysis of the primary outcome will be based on the mean number of units of ABPs within the first 24 hours (λ_1 – control group and λ_2 – intervention group).

To demonstrate that the early administration of FC and PCC is clinically superior to the standard of care, with respect to the mean number of ABPs administered within 24 hours of arrival at the trauma bay/ED, a two-sample, one-sided test of the pair of hypotheses: H_0: $RR \geq RR_0$ vs. Ha: $RR < RR_0$ will be carried out with a

type I error probability of $\alpha = 0.025$. Here, λ_1 and λ_2 denote the mean number of ABPs (RBCs + FP + platelets) in the control group (standard of care) and intervention group, respectively, RR is the ratio λ_2/λ_1, and RR_0 will be set equal to 1.0 to test for superiority. A mean difference in 5 U of the composite outcome (mean 15 U in the control group and mean 10 U in the intervention group) is considered as a clinically meaningful difference that should be detected with at least 80% power.

Testing of the hypothesis will be performed in the context of a counting regression model (generalized linear model for count data with log-link function and a negative binomial error term), with treatment group as main effect. Inferences will be based on the one-sided 97.5% confidence interval (CI) for the ratio λ_2 / λ_1 derived from the estimated least square means (LSmeans) of this model. Superiority will be concluded if the upper limit of this CI is strictly less than $R_0 = 1.0$ (i.e. the mean number of ABPs is larger in the control group).

Secondary Endpoints

The following measurement will be considered secondary endpoint in the analysis of efficacy of the study treatments:

- Total number of units of RBCs transfused within the first 24 hours following arrival at the trauma bay/ED

Other endpoints in the analysis of efficacy include:

- Total and individual number of units and volumes (liters) of ABPs (RBCs, FP and platelets) transfused within 6 hours, 24 hours and within 7 days post arrival at the trauma bay/ED
- Total volume of crystalloids and other colloids administered within the first 6 and 24 hours following arrival at the trauma bay/ED
- Rescue use of hemostatic agents (fibrinogen concentrate and rFVIIa) within the first 24 hours following arrival at the trauma bay/ED
- Laboratory endpoints, including thromboelastometry measurements, where available
- Days out of hospital within the first 28 days following arrival at the trauma bay/ED
- Time to death over the first 28 days following arrival at the trauma bay/ED

9.2.2 Safety Analysis Plan

The following measurements will be considered secondary endpoints in the analysis of safety of the study treatments:

- Incidence of thromboembolic events, from arrival at the trauma bay/ED, up to 28 days
- Ventilator-free days up to Day 28 following arrival at the trauma bay/ED

Other endpoints in the analysis of safety of the study treatments include:

- All documented adverse events (AEs) and serious adverse events (SAEs) during the first 28 days following arrival, including MOF, ACS and LCS, transfusion reactions, and TEAEs
- Duration of ICU stay
- 28-day all-cause mortality

The safety analysis population (SAF) will include all randomized patients who receive any of the interventional products in the first MHP pack or beyond the intended first-line treatment and agree to remain in the study after consenting. Safety outcomes will be analyzed analogously to the primary endpoint, presenting point estimates and two-sided 95% CIs in addition to descriptive statistics.

Adverse Events, Including Thromboembolic Events

AEs will be coded according to the latest Medical Dictionary for Regulatory Activities (MedDRA) version as specified in the Data Management Plan. The analysis will focus on treatment emergent adverse events (TEAEs), i.e., AEs that started or worsened after start of infusion with IMP.

All TEAEs, related TEAEs (i.e., AEs probably or possibly related to the IMP), and serious TEAEs will be summarized and tabulated according to primary system organ class and preferred term. TEAEs leading to death and TEAEs resulting in withdrawal from the study, respectively, will be tabulated using frequency tables if a reasonable number of events of this type are observed.

Analogous frequency tables for thromboembolic events (as recorded on the pertinent eCRF page and those identified using standardized MedDRA queries [SMQs]) will be provided.

Patient listings will be provided for patients with SAEs, thromboembolic events, AEs leading to withdrawal from study, and AEs leading to death. These SAE listings will also include patients enrolled but not randomized.

Mortality

The number of patients who died (all-cause mortality) will be summarized. Date and time of death will be recorded up to 28 days following arrival at the trauma bay/ED. Cause of death will be blindly adjudicated by an independent reviewer physician and one of the investigators, and defined as:

- Mainly due to exsanguination
- Mainly neurological/due to traumatic brain injury
- Mainly due to MOF/sepsis
- Other, with specific cause detailed

A possible difference between treatment groups will be estimated by a risk ratio with 95% CI. Kaplan-Meier estimates for the time to death distribution will be calculated and graphically presented.

Routine Laboratory Data

All laboratory values will be classified as normal or abnormal according to the laboratories' normal ranges and indicated as clinically significant or not clinically significant by the investigator on specified ranges. The following approaches will be taken for each laboratory parameter for the statistical analysis:

- Quantitative data will be examined for trends using descriptive analysis (number of patients, number of missing values, mean, SD, median, quartiles, minimum, maximum) of actual values at each scheduled time point and changes from baseline to each scheduled time point
- Qualitative data based on reference ranges will be described according to the categories (i.e., low, normal, high)

- Shift tables illustrating changes with respect to the laboratories' normal ranges between baseline and a defined scheduled time point
- Number and frequency of patients with clinically significant laboratory values. A separate patient listing will be provided

9.2.3 Handling of Missing Data

In general, missing data will not be imputed.

9.3 Randomization, Stratification, and Code Release

Eligible patients will be randomly assigned to the intervention group or control group. Randomization will occur via a computer-generated random-block size allocation sequence generated by a biostatistician. In order to assure balanced groups, the randomization will be stratified by hospital sites. Sealed envelopes containing these numbers for treatment allocation will be maintained in the blood banks of the participating sites.

The medical laboratory technologist from the Blood Bank will randomize patients to one of the two groups once eligibility has been confirmed. Patients will be identified using a sequential numbering system.

9.4 Adaptive design (Interim Analysis)

Due to the inherent variability in the primary endpoint and a yet substantial uncertainty about the effect size, an adaptive design approach will be used. For this, a single interim analysis will be performed after 120 patients have completed the study. Primary aim of this interim analysis is to calculate the p-value and conditional power of the test statistic for the primary endpoint and perform a sample size re-assessment. This will be an unblinded interim analysis performed by an independent statistician who will report the results only to the independent data safety monitoring committee (IDSMC) which will make recommendations to the sponsor without revealing the treatment groups. The study design will follow a group sequential design with O'Brien-Fleming error-spending function, a futility boundary and sample size re-estimation based on conditional power. Hence, the recommendation of the IDSMC can include:

- To continue the trial as planned until 350 patients have completed the study,
- To stop the trial for demonstrated superiority at the interim analysis,
- To stop the trial at the interim for futility (e.g. conditional power less than 25%) or for requiring an increase in sample size that is considered unfeasible (e.g. total sample size larger than 450),
- To continue the trial with a modified sample size.

9.5 Subgroup Analysis

To assess a possible different efficacy and safety profile in different patient subpopulations, the statistical analysis of the subgroups will be restricted to the mITT analysis population and will involve only selected efficacy and safety endpoints, defined a priori as:

1. Patients with blunt versus penetrating trauma (excluding patients with trauma resulting from both mechanisms)
2. Patients with versus those without traumatic brain injury
3. Patients less than 60 years of age versus those 60 years of age and older
4. Patients massively transfused (10 RBC U or more) versus those not massively transfused
5. Subgroups by study site, depending on the enrollment volume (1–20, 20–50, >50 patients)

Further details of the interim and subgroup analyses are described in the respective sections of the SAP.

10 ETHICAL/REGULATORY, LEGAL AND ADMINISTRATIVE ASPECTS

10.1 Ethical/Regulatory Framework

This study will be conducted in accordance with the ethical principles laid down in the Declaration of Helsinki. The study protocol and any subsequent amendment(s) will be submitted to an REB and to the Regulatory Authority. The study will be conducted in compliance with the protocol, GCP guidelines, and applicable regulatory requirements.

The regulatory application or submission for regulatory approval will be made by the Sponsor or designated third party (e.g., CRO).

10.2 Approval of Study Documents

The study protocol, a sample of the patient information and informed consent form, any other materials provided to the patients, and further requested information will be submitted by the Sponsor or the Investigator to the appropriate REB and the Regulatory Authority. The study must be approved by the REB and the Regulatory Authority before any IMPs may be shipped to the study sites and any patient is exposed to a study-related procedure.

The Sponsor, the Investigator and any third party (e.g., CRO) involved in obtaining approval must inform each other in writing that all ethical and legal requirements have been met before the first patient is enrolled in the study.

10.3 Patient Information and Informed Consent

This study will compare hemostatic therapies that are currently within the standard-of-care for trauma patients and poses no additional risks to patients and entails no additional interventions outside of normal clinical care. Moreover, due to the emergency nature of the condition being studied (i.e., patients at risk of massive hemorrhage), the trial will include only patients who are incapable of providing informed consent at the time the therapy is needed and in whom delays in obtaining surrogate consent can be severely detrimental to their well-being. Thus, we will employ a deferred informed consent approach [99,100].

10.3.1 Deferred Consent

We will obtain consent from the patient or an SDM as soon as possible after randomization for collection and analysis of patient data. The Investigator (or delegate as appropriate) will obtain freely given consent from each patient (or SDM) after an appropriate explanation of the aims, methods, anticipated benefits, potential hazards, and any other aspect of the study which is relevant to the decision to continue to participate.

The Investigator (or delegate) will explain that the patients are completely free to withdraw from the study at any time, without any consequences for their further care and without the need to justify. Each patient will be informed that his/her medical (source) records may be reviewed by the study monitor, a quality assurance auditor, or a health authority inspector, in accordance with applicable regulations, and that these persons are bound by confidentiality obligations.

Every attempt will be made to have the informed consent form signed, with name and date and time noted by the patient (or SDM), before the patient is exposed to any further study-related procedures, namely evaluation and data collection. In unique situations in which all attempts to obtain written consent have been exhausted, telephone consent will be accepted. This may be due to pandemic restrictions to hospital visitation prevent the SDM from coming in-person to the facility, or they do not have access or are unable to proficiently use the computer. As dictated in the telephone script, a witness will be present to confirm the telephone consent process was adhered to and confirm the patient/SDM agreement to participate in the trial.In addition to the challenges described above, other situations may further contribute to failure in obtaining the consent. In patients in extremis, who usually expire within the six to 24 hours post admission/enrollment to the study, it is extremely difficult to find patient`s or SDM`s contact information. It is not uncommon that friends or relatives of young trauma patients who are victims of a violent mechanism of injury (usually gunshot wounds, but not limited to it) come to the hospital to be updated about the death are hesitant to speak to hospital staff and do not leave contact information. As the body is moved to the coroner`s office, obtaining further information (including identification of the SDM) is nearly impossible.

The consenting process continues to be difficult in case they survive but lose the capacity of providing (or not) consent. Ongoing efforts to obtain contact information to approach the SDM, including with the support of social workers, is made. However, most commonly these attempts are not successful in these marginalized patients.

Due to these extreme challenges and the fact that the consenting process starts after the intervention, integrity of data and validity of the trial may be affected, if the number of incomplete CRFs exceed a few percent of the total number of patients. Furthermore, not being able to collect data related to adverse events (i.e., thromboembolic complications) may affect the evaluation of the safety of the two comparative treatments.

In situations where the patient has expired or has not recovered to provide consent and research staff have been unsuccessful in contacting the SDM after at least 3 attempts, all patient data will be collected. Please, see Appendix 6 for a summary of the consenting process.

This consent process meets the criteria for alterations of the informed consent according to the Tri-council policy statement for the ethical conduct for research involving humans: it involves a serious threat to the participants that requires immediate intervention, no standard efficacious care exists, risk is not greater than that involved in standard efficacious care, participant is unconscious or lacks capacity to understand the risks, methods and purposes of the study, and third party authorization cannot be secured in sufficient time.

10.4 Protocol Amendments

Any amendments will be submitted to the competent REB and any authority as required by applicable regulations.

REB approval will, at a minimum, be requested for any change to this protocol which could affect the safety of the patients, the objective or design of the study, any increase in dosage or duration of exposure to the IMPs, an increase in the number of patients treated, the addition of a new test or procedure, or the dropping of a test intended to monitor safety.

10.5 Confidentiality of Patient Data

The Investigator will ensure that the patient's confidentiality is preserved. On CRFs or any other documents submitted to the Sponsor, the patients will not be identified by their names, but by a unique patient identifier. Documents not intended for submission to the Sponsor, i.e., the confidential patient identification code list, original consent forms, and source records, will be maintained by the Investigator in strict confidence.

11 QUALITY CONTROL AND QUALITY ASSURANCE

11.1 Periodic Monitoring

The monitor will contact and visit the Investigator periodically to review all study-related source data/records, verify the adherence to the protocol and the completeness, correctness and accuracy of all CRF entries compared to source data. The Investigator will co-operate with the monitor to ensure that any discrepancies identified are resolved.

For this study, the first monitoring visit shall take place shortly after the inclusion of the first patient. Thereafter, monitoring frequency will depend on study progress.

The monitor must be given direct access to source documents (original documents, data and records). Direct access includes permission to examine, analyze, verify, and reproduce any records and reports that are important to the evaluation of the clinical study. Source data will be available for all data in the CRFs, including all laboratory results.

11.2 Audit and Inspection

The Investigator will make all study-related source data and records available to a qualified quality assurance auditor or REB and regulatory inspectors, after reasonable notice. The main purposes of an audit or inspection are to confirm that the rights and welfare of the patients have been adequately protected, and that all data relevant for the assessment of safety and efficacy of the IMPs have been captured.

12 REPORTING AND PUBLICATION

12.1 Clinical Study Report

A clinical study report (in accordance with relevant guidelines) will be prepared by the Sponsor after completion of the study. The Coordinating Investigator will approve the final study report after review.

12.2 Publication Policy

The results of this study will be published and may be presented at scientific meetings.

In accordance with standard editorial and ethical practice, the Investigator will publish the multi-center data only in their entirety and not as individual center data. Authorship will be determined by mutual agreement. Any subsequent publications based on subsets of the data will require approval from the Sponsor.

13 LIABILITIES AND INSURANCE

In order to cover any potential damage or injury occurring to a patient in association with the IMPs or participation in the study, the Investigators and or their institutions will contract insurance in accordance with local regulations.

The Investigators are responsible for dispensing the IMPs according to this protocol and for its secure storage and safe handling throughout the study.

14 REFERENCES

1. Thygesen K, Alpert JS, Jaffe AS, Chaitman BR, Bax JJ, Morrow DA, et al. Fourth Universal Definition of Myocardial Infarction (2018). Circulation. 2018;138(20):e618-e51.
2. Kernan WN, Ovbiagele B, Black HR, Bravata DM, Chimowitz MI, Ezekowitz MD, et al. Guidelines for the prevention of stroke in patients with stroke and transient ischemic attack: a guideline for healthcare professionals from the American Heart Association/American Stroke Association. Stroke. 2014;45(7):2160-236.
3. Schoenfeld DA, Bernard GR, Network A. Statistical evaluation of ventilator-free days as an efficacy measure in clinical trials of treatments for acute respiratory distress syndrome. Crit Care Med. 2002;30(8):1772-7.
4. Jones AE, Trzeciak S, Kline JA. The Sequential Organ Failure Assessment score for predicting outcome in patients with severe sepsis and evidence of hypoperfusion at the time of emergency department presentation. Crit Care Med. 2009;37(5):1649-54.
5. Kirkpatrick AW, Roberts DJ, De Waele J, Jaeschke R, Malbrain ML, De Keulenaer B, et al. Intra-abdominal hypertension and the abdominal compartment syndrome: updated consensus definitions and clinical practice guidelines from the World Society of the Abdominal Compartment Syndrome. Intensive Care Med. 2013;39(7):1190-206.
6. Wall CJ, Lynch J, Harris IA, Richardson MD, Brand C, Lowe AJ, et al. Clinical practice guidelines for the management of acute limb compartment syndrome following trauma. ANZ J Surg. 2010;80(3):151-6.
7. International Society of Blood Transfusion Working Party on Haemovigilance. Proposed standard definitions for surveillance of non infectious adverse transfusion reactions. Last updated 2018. Available at: https://www.isbtweb.org/fileadmin/user_upload/Proposed_definitions_2011_surveillance_non_infectious_adverse_reactions_haemovigilance_incl_TRALI_correction_2013_TACO_correction_2018.pdf. Accessed Feb 05, 2020.
8. Centers for Disease Control and Prevention. Web-based Injury Statistics Query and Reporting System (WISQARS). Available at: http://www.cdc.gov/injury/wisqars. Accessed Jul 25, 2019.
9. Kauvar DS, Lefering R, Wade CE. Impact of hemorrhage on trauma outcome: an overview of epidemiology, clinical presentations, and therapeutic considerations. J Trauma. 2006;60(6 Suppl):S3-11.
10. Simmons JW, Pittet JF, Pierce B. Trauma-Induced Coagulopathy. Curr Anesthesiol Rep. 2014;4(3):189-99.
11. Yucel N, Ozturk Demir T, Derya S, Oguzturk H, Bicakcioglu M, Yetkin F. Potential Risk Factors for In-Hospital Mortality in Patients with Moderate-to-Severe Blunt Multiple Trauma Who Survive Initial Resuscitation. Emerg Med Int. 2018;2018:6461072.
12. Brohi K, Singh J, Heron M, Coats T. Acute traumatic coagulopathy. J Trauma. 2003;54(6):1127-30.
13. MacLeod JB, Lynn M, McKenney MG, Cohn SM, Murtha M. Early coagulopathy predicts mortality in trauma. J Trauma. 2003;55(1):39-44.
14. Maegele M, Lefering R, Yucel N, Tjardes T, Rixen D, Paffrath T, et al. Early coagulopathy in multiple injury: an analysis from the German Trauma Registry on 8724 patients. Injury. 2007;38(3):298-304.
15. Brohi K, Cohen MJ, Ganter MT, Matthay MA, Mackersie RC, Pittet JF. Acute traumatic coagulopathy: initiated by hypoperfusion: modulated through the protein C pathway? Ann Surg. 2007;245(5):812-8.
16. Niles SE, McLaughlin DF, Perkins JG, Wade CE, Li Y, Spinella PC, et al. Increased mortality associated with the early coagulopathy of trauma in combat casualties. J Trauma. 2008;64(6):1459-63.

17. Frith D, Brohi K. The pathophysiology of trauma-induced coagulopathy. Curr Opin Crit Care. 2012;18(6):631-6.
18. Hess JR, Brohi K, Dutton RP, Hauser CJ, Holcomb JB, Kluger Y, et al. The coagulopathy of trauma: a review of mechanisms. J Trauma. 2008;65(4):748-54.
19. Hiippala S. Replacement of massive blood loss. Vox Sang. 1998;74 Suppl 2:399-407.
20. Vernon T, Morgan M, Morrison C. Bad blood: A coagulopathy associated with trauma and massive transfusion review. Acute Med Surg. 2019;6(3):215-22.
21. Levy JH, Welsby I, Goodnough LT. Fibrinogen as a therapeutic target for bleeding: A review of critical levels and replacement therapy. Transfusion. 2014;54(5):1389-405.
22. Franchini M, Lippi G. Fibrinogen replacement therapy: a critical review of the literature. Blood Transfus. 2012;10(1):23-7.
23. Lowe GD, Rumley A, Mackie IJ. Plasma fibrinogen. Ann Clin Biochem. 2004;41(Pt 6):430-40.
24. Fries D, Innerhofer P, Reif C, Streif W, Klingler A, Schobersberger W, et al. The effect of fibrinogen substitution on reversal of dilutional coagulopathy: An in vitro model. Anesth Analg. 2006;102(2):347-51.
25. British Committee for Standards in H, Stainsby D, MacLennan S, Thomas D, Isaac J, Hamilton PJ. Guidelines on the management of massive blood loss. Br J Haematol. 2006;135(5):634-41.
26. Hiippala ST, Myllyla GJ, Vahtera EM. Hemostatic factors and replacement of major blood loss with plasma-poor red cell concentrates. Anesth Analg. 1995;81(2):360-5.
27. Bolliger D, Szlam F, Molinaro RJ, Rahe-Meyer N, Levy JH, Tanaka KA. Finding the optimal concentration range for fibrinogen replacement after severe haemodilution: An in vitro model. Br J Anaesth. 2009;102(6):793-9.
28. Nielsen VG, Cohen BM, Cohen E. Effects of coagulation factor deficiency on plasma coagulation kinetics determined via thrombelastography: Critical roles of fibrinogen and factors II, VII, X, and XII. Acta Anaesthesiol Scand. 2005;49(2):222-31.
29. Lang T, Johanning K, Metzler H, Piepenbrock S, Solomon C, Rahe-Meyer N, et al. The effects of fibrinogen levels on thromboelastometric variables in the presence of thrombocytopenia. Anesth Analg. 2009;108(3):751-8.
30. Dempfle CE, Kalsch T, Elmas E, Suvajac N, Lucke T, Munch E, et al. Impact of fibrinogen concentration in severely ill patients on mechanical properties of whole blood clots. Blood Coagul Fibrinolysis. 2008;19(8):765-70.
31. Karkouti K, Callum J, Crowther MA, McCluskey SA, Pendergrast J, Tait G, et al. The relationship between fibrinogen levels after cardiopulmonary bypass and large volume red cell transfusion in cardiac surgery: an observational study. Anesth Analg. 2013;117(1):14-22.
32. Karlsson M, Ternstrom L, Hyllner M, Baghaei F, Nilsson S, Jeppsson A. Plasma fibrinogen level, bleeding, and transfusion after on-pump coronary artery bypass grafting surgery: A prospective observational study. Transfusion. 2008;48(10):2152-8.
33. Blome M, Isgro F, Kiessling AH, Skuras J, Haubelt H, Hellstern P, et al. Relationship between factor XIII activity, fibrinogen, haemostasis screening tests and postoperative bleeding in cardiopulmonary bypass surgery. Thromb Haemost. 2005;93(6):1101-7.
34. Ternstrom L, Radulovic V, Karlsson M, Baghaei F, Hyllner M, Bylock A, et al. Plasma activity of individual coagulation factors, hemodilution and blood loss after cardiac surgery: A prospective observational study. Thromb Res. 2010;126(2):e128-e33.

35. Spahn DR, Bouillon B, Cerny V, Duranteau J, Filipescu D, Hunt BJ, et al. The European guideline on management of major bleeding and coagulopathy following trauma: fifth edition. Crit Care. 2019;23(1):98.
36. Callum JL, Karkouti K, Lin Y. Cryoprecipitate: The current state of knowledge. Transfus Med Rev. 2009;23(3):177-88.
37. Pereira A. Cryoprecipitate versus commercial fibrinogen concentrate in patients who occasionally require a therapeutic supply of fibrinogen: risk comparison in the case of an emerging transfusion-transmitted infection. Haematologica. 2007;92(6):846-9.
38. Webert KE, Cserti CM, Hannon J, Lin Y, Pavenski K, Pendergrast JM, et al. Proceedings of a Consensus Conference: pathogen inactivation-making decisions about new technologies. Transfus Med Rev. 2008;22(1):1-34.
39. Theodoulou A, Berryman J, Nathwani A, Scully M. Comparison of cryoprecipitate with fibrinogen concentrate for acquired hypofibrinogenaemia. Transfus Apher Sci. 2012;46(2):159-62.
40. Ahmed S, Harrity C, Johnson S, Varadkar S, McMorrow S, Fanning R, et al. The efficacy of fibrinogen concentrate compared with cryoprecipitate in major obstetric haemorrhage--an observational study. Transfus Med. 2012;22(5):344-9.
41. Collins PW, Solomon C, Sutor K, Crispin D, Hochleitner G, Rizoli S, et al. Theoretical modelling of fibrinogen supplementation with therapeutic plasma, cryoprecipitate, or fibrinogen concentrate. Br J Anaesth. 2014;113(4):585-95.
42. Callum J, Farkouh ME, Scales DC, Heddle NM, Crowther M, Rao V, et al. Effect of Fibrinogen Concentrate vs Cryoprecipitate on Blood Component Transfusion After Cardiac Surgery: The FIBRES Randomized Clinical Trial. JAMA. 2019:1-11.
43. Curry N, Foley C, Wong H, Mora A, Curnow E, Zarankaite A, et al. Early fibrinogen concentrate therapy for major haemorrhage in trauma (E-FIT 1): results from a UK multi-centre, randomised, double blind, placebo-controlled pilot trial. Crit Care. 2018;22(1):164.
44. Weiss G, Lison S, Glaser M, Herberger S, Johanning K, Strasser T, et al. Observational study of fibrinogen concentrate in massive hemorrhage: evaluation of a multicenter register. Blood Coagul Fibrinolysis. 2011;22(8):727-34.
45. Aubron C, Reade MC, Fraser JF, Cooper DJ. Efficacy and safety of fibrinogen concentrate in trauma patients--a systematic review. J Crit Care. 2014;29(3):471 e11-7.
46. Kozek-Langenecker S, Sorensen B, Hess JR, Spahn DR. Clinical effectiveness of fresh frozen plasma compared with fibrinogen concentrate: a systematic review. Crit Care. 2011;15(5):R239.
47. Wikkelsø A, Lunde J, Johansen M, Stensballe J, Wetterslev J, Moller AM, et al. Fibrinogen concentrate in bleeding patients. Cochrane Database Syst Rev. 2013(8):CD008864.
48. Mengoli C, Franchini M, Marano G, Pupella S, Vaglio S, Marietta M, et al. The use of fibrinogen concentrate for the management of trauma-related bleeding: a systematic review and meta-analysis. Blood Transfus. 2017;15(4):318-24.
49. Nascimento B, Callum J, Tien H, Peng H, Rizoli S, Karanicolas P, et al. Fibrinogen in the initial resuscitation of severe trauma (FiiRST): a randomized feasibility trial. Br J Anaesth. 2016;117(6):775-82.
50. Chowdary P, Tang A, Watson D, Besser M, Collins P, Creagh MD, et al. Retrospective Review of a Prothrombin Complex Concentrate (Beriplex P/N) for the Management of Perioperative Bleeding Unrelated to Oral Anticoagulation. Clin Appl Thromb Hemost. 2018;24(7):1159-69.
51. Sheffield WP, Bhakta V, Yi QL, Jenkins C. Stability of Thawed Apheresis Fresh-Frozen Plasma Stored for up to 120 Hours at 1 degrees C to 6 degrees C. J Blood Transfus. 2016;2016:6260792.

52. Chai-Adisaksopha C, Hillis C, Siegal DM, Movilla R, Heddle N, Iorio A, et al. Prothrombin complex concentrates versus fresh frozen plasma for warfarin reversal. A systematic review and meta-analysis. Thromb Haemost. 2016;116(5):879-90.
53. Pandey S, Vyas GN. Adverse effects of plasma transfusion. Transfusion. 2012;52 Suppl 1:65S-79S.
54. Desborough M, Sandu R, Brunskill SJ, Doree C, Trivella M, Montedori A, et al. Fresh frozen plasma for cardiovascular surgery. Cochrane Database Syst Rev. 2015(7):CD007614.
55. Holness L, Knippen MA, Simmons L, Lachenbruch PA. Fatalities caused by TRALI. Transfus Med Rev. 2004;18(3):184-8.
56. Narick C, Triulzi DJ, Yazer MH. Transfusion-associated circulatory overload after plasma transfusion. Transfusion. 2012;52(1):160-5.
57. Shaheen AW, Crandall ML, Nicolson NG, Smith-Singares E, Merlotti GJ, Jalundhwala Y, et al. Abdominal compartment syndrome in trauma patients: New insights for predicting outcomes. J Emerg Trauma Shock. 2016;9(2):53-7.
58. Sarani B, Dunkman WJ, Dean L, Sonnad S, Rohrbach JI, Gracias VH. Transfusion of fresh frozen plasma in critically ill surgical patients is associated with an increased risk of infection. Crit Care Med. 2008;36(4):1114-8.
59. Bjursten H, Dardashti A, Ederoth P, Bronden B, Algotsson L. Increased long-term mortality with plasma transfusion after coronary artery bypass surgery. Intensive Care Med. 2013;39(3):437-44.
60. Khan H, Belsher J, Yilmaz M, Afessa B, Winters JL, Moore SB, et al. Fresh-frozen plasma and platelet transfusions are associated with development of acute lung injury in critically ill medical patients. Chest. 2007;131(5):1308-14.
61. Inaba K, Branco BC, Rhee P, Blackbourne LH, Holcomb JB, Teixeira PG, et al. Impact of plasma transfusion in trauma patients who do not require massive transfusion. J Am Coll Surg. 2010;210(6):957-65.
62. Watson GA, Sperry JL, Rosengart MR, Minei JP, Harbrecht BG, Moore EE, et al. Fresh frozen plasma is independently associated with a higher risk of multiple organ failure and acute respiratory distress syndrome. J Trauma. 2009;67(2):221-7.
63. Percy CL, Hartmann R, Jones RM, Balachandran S, Mehta D, Dockal M, et al. Correcting thrombin generation ex vivo using different haemostatic agents following cardiac surgery requiring the use of cardiopulmonary bypass. Blood Coagul Fibrinolysis. 2015;26(4):357-67.
64. Ghadimi K, Levy JH, Welsby IJ. Prothrombin Complex Concentrates for Bleeding in the Perioperative Setting. Anesth Analg. 2016;122(5):1287-300.
65. Schubert P, Culibrk B, Karwal S, Slichter SJ, Devine DV. Optimization of platelet concentrate quality: application of proteomic technologies to donor management. J Proteomics. 2012;76 Spec No.:329-36.
66. Raval JS, Waters JH, Seltsam A, Scharberg EA, Richter E, Kameneva MV, et al. Menopausal status affects the susceptibility of stored RBCs to mechanical stress. Vox Sang. 2011;100(4):418-21.
67. Grottke O, Rossaint R, Henskens Y, van Oerle R, Ten Cate H, Spronk HM. Thrombin generation capacity of prothrombin complex concentrate in an in vitro dilutional model. PLoS One. 2013;8(5):e64100.
68. Schöchl H, Grottke O, Sutor K, Dony K, Schreiber M, Ranucci M, et al. Theoretical Modeling of Coagulation Management With Therapeutic Plasma or Prothrombin Complex Concentrate. Anesth Analg. 2017;125(5):1471-4.
69. Godier A, Greinacher A, Faraoni D, Levy JH, Samama CM. Use of factor concentrates for the management of perioperative bleeding: guidance from the SSC of the ISTH. J Thromb Haemost. 2018;16(1):170-4.

70. Barco S, Picchi C, Trinchero A, Middeldorp S, Coppens M. Safety of prothrombin complex concentrate in healthy subjects. Br J Haematol. 2017;176(4):664-6.
71. Grottke O, Braunschweig T, Spronk HM, Esch S, Rieg AD, van Oerle R, et al. Increasing concentrations of prothrombin complex concentrate induce disseminated intravascular coagulation in a pig model of coagulopathy with blunt liver injury. Blood. 2011;118(7):1943-51.
72. Mitterlechner T, Innerhofer P, Streif W, Lodl M, Danninger T, Klima G, et al. Prothrombin complex concentrate and recombinant prothrombin alone or in combination with recombinant factor X and FVIIa in dilutional coagulopathy: a porcine model. J Thromb Haemost. 2011;9(4):729-37.
73. Majeed A, Eelde A, Agren A, Schulman S, Holmstrom M. Thromboembolic safety and efficacy of prothrombin complex concentrates in the emergency reversal of warfarin coagulopathy. Thromb Res. 2012;129(2):146-51.
74. Bruce D, Nokes TJ. Prothrombin complex concentrate (Beriplex P/N) in severe bleeding: experience in a large tertiary hospital. Crit Care. 2008;12(4):R105.
75. Jehan F, Aziz H, O'Keeffe T, Khan M, Zakaria ER, Hamidi M, et al. The role of four-factor prothrombin complex concentrate in coagulopathy of trauma: A propensity matched analysis. J Trauma Acute Care Surg. 2018;85(1):18-24.
76. Goldstein JN, Refaai MA, Milling TJ, Jr., Lewis B, Goldberg-Alberts R, Hug BA, et al. Four-factor prothrombin complex concentrate versus plasma for rapid vitamin K antagonist reversal in patients needing urgent surgical or invasive interventions: a phase 3b, open-label, non-inferiority, randomised trial. Lancet. 2015;385(9982):2077-87.
77. Rahe-Meyer N, Solomon C, Hanke A, Schmidt DS, Knoerzer D, Hochleitner G, et al. Effects of fibrinogen concentrate as first-line therapy during major aortic replacement surgery: a randomized, placebo-controlled trial. Anesthesiology. 2013;118(1):40-50.
78. Schöchl H, Nienaber U, Hofer G, Voelckel W, Jambor C, Scharbert G, et al. Goal-directed coagulation management of major trauma patients using thromboelastometry (ROTEM)-guided administration of fibrinogen concentrate and prothrombin complex concentrate. Crit Care. 2010;14(2):R55.
79. Schöchl H, Nienaber U, Maegele M, Hochleitner G, Primavesi F, Steitz B, et al. Transfusion in trauma: thromboelastometry-guided coagulation factor concentrate-based therapy versus standard fresh frozen plasma-based therapy. Crit Care. 2011;15(2):R83.
80. Innerhofer P, Fries D, Mittermayr M, Innerhofer N, von Langen D, Hell T, et al. Reversal of trauma-induced coagulopathy using first-line coagulation factor concentrates or fresh frozen plasma (RETIC): a single-centre, parallel-group, open-label, randomised trial. Lancet Haematol. 2017;4(6):e258-e71.
81. Schöchl H, Voelckel W, Grassetto A, Schlimp CJ. Practical application of point-of-care coagulation testing to guide treatment decisions in trauma. J Trauma Acute Care Surg. 2013;74(6):1587-98.
82. Gorlinger K, Dirkmann D, Hanke AA, Kamler M, Kottenberg E, Thielmann M, et al. First-line therapy with coagulation factor concentrates combined with point-of-care coagulation testing is associated with decreased allogeneic blood transfusion in cardiovascular surgery: a retrospective, single-center cohort study. Anesthesiology. 2011;115(6):1179-91.
83. Weber CF, Zacharowski K, Meybohm P, Adam EH, Hofer S, Brun K, et al. Hemotherapy algorithms for coagulopathic cardiac surgery patients. Clin Lab. 2014;60(6):1059-63.
84. Zeeshan M, Hamidi M, Feinstein AJ, Gries L, Jehan F, Sakran J, et al. Four-factor prothrombin complex concentrate is associated with improved survival in trauma-related hemorrhage: A nationwide propensity matched analysis. J Trauma Acute Care Surg. 2019;87(2):274-81.
85. Holcomb JB, Fox EE, Zhang X, White N, Wade CE, Cotton BA, et al. Cryoprecipitate use in the PROMMTT study. J Trauma Acute Care Surg. 2013;75(1 Suppl 1):S31-9.

86. Shih AW, Al Khan S, Wang AY, Dawe P, Young PY, Greene A, et al. Systematic reviews of scores and predictors to trigger activation of massive transfusion protocols. J Trauma Acute Care Surg. 2019;87(3):717-29.
87. Ross C, Rangarajan S, Karimi M, Toogeh G, Apte S, Lissitchkov T, et al. Pharmacokinetics, clot strength and safety of a new fibrinogen concentrate: randomized comparison with active control in congenital fibrinogen deficiency. J Thromb Haemost. 2018;16(2):253-61.
88. Lissitchkov T, Madan B, Djambas Khayat C, Zozulya N, Ross C, Karimi M, et al. Efficacy and safety of a new human fibrinogen concentrate in patients with congenital fibrinogen deficiency: an interim analysis of a Phase III trial. Transfusion. 2018;58(2):413-22.
89. Kozek-Langenecker S, Fries D, Spahn DR, Zacharowski K. III. Fibrinogen concentrate: clinical reality and cautious Cochrane recommendation. Br J Anaesth. 2014;112(5):784-7.
90. Karkouti K, Callum J, Rao V, Heddle N, Farkouh ME, Crowther MA, et al. Protocol for a phase III, non-inferiority, randomised comparison of a new fibrinogen concentrate versus cryoprecipitate for treating acquired hypofibrinogenaemia in bleeding cardiac surgical patients: the FIBRES trial. BMJ Open. 2018;8(4):e020741.
91. Solomon C, Hagl C, Rahe-Meyer N. Time course of haemostatic effects of fibrinogen concentrate administration in aortic surgery. Br J Anaesth. 2013;110(6):947-56.
92. Rodgers GM. Prothrombin complex concentrates in emergency bleeding disorders. Am J Hematol. 2012;87(9):898-902.
93. National Advisory Committee on Blood and Blood Products. Recommendations for use of prothrombin complex concentrate in Canada. May 2014. Available at: https://www.nacblood.ca/resources/guidelines/downloads/PCC-Recommendations-Final-2014-05-16.pdf. Accessed Feb 05, 2020.
94. Rothmann MD, Wiens BL, Chan ISF. Design and analysis of non-inferiority trials. 1st ed. Chow SC, Jones B, Liu JP, Pease KE, Turnbull BW, editors: Chapman & Hall/CRC; 2012 2012.
95. Freedman B. Placebo-controlled trials and the logic of clinical purpose. IRB. 1990;12(6):1-6.
96. Burnouf T, Radosevich M. Nanofiltration of plasma-derived biopharmaceutical products. Haemophilia. 2003;9(1):24-37.
97. Bates SM, Jaeschke R, Stevens SM, Goodacre S, Wells PS, Stevenson MD, et al. Diagnosis of DVT: Antithrombotic Therapy and Prevention of Thrombosis, 9th ed: American College of Chest Physicians Evidence-Based Clinical Practice Guidelines. Chest. 2012;141(2 Suppl):e351S-e418S.
98. Singer M, Deutschman CS, Seymour CW, Shankar-Hari M, Annane D, Bauer M, et al. The Third International Consensus Definitions for Sepsis and Septic Shock (Sepsis-3)Consensus Definitions for Sepsis and Septic ShockConsensus Definitions for Sepsis and Septic Shock. JAMA. 2016;315(8):801-10.
99. Shaw DA. HEAT-PPCI sheds light on consent in pragmatic trials. The Lancet. 2014;384(9957):1826-7.
100. Jansen TC, Kompanje EJ, Bakker J. Deferred proxy consent in emergency critical care research: ethically valid and practically feasible. Crit Care Med. 2009;37(1 Suppl):S65-8.

15 APPENDICES

Appendix 1. Massive Hemorrhage Protocol Activation Criteria Collected During Protocol Development at Each Potential Study Site

Participating site	MHP activation criteria
Sunnybrook Health Sciences Centre	• Life-threatening bleeding situation requiring mobilization of blood bank, laboratory and clinical resources<br>• Anticipated need for at least 4 U of RBC immediately and component therapy (platelets, plasma and fibrinogen)<br>• Systolic blood pressure less than 90 mmHg and/or requiring inotropes
Saint Michael's Hospital	• Penetrating trauma AND persistent hypotension (2 measurements of systolic blood pressure <90 mmHg 5 min apart in the ED)<br>• Blunt trauma AND persistent hypotension AND suspected bleeding<br>• A recognized need for un-crossmatched RBC<br>• Known or suspected cardiac rupture, aortic rupture or atrial leak
Montreal General Hospital	• Anticipated total body volume loss of blood within a 24-hour period = 8–10 U RBCs for an average adult<br>• Or half body volume loss in <3 hours
Foothills Medical Centre	• MD discretion
Vancouver General Hospital	• Trauma patient with active hemorrhage AND systolic blood pressure <90 mmHg or impalpable radial pulse AND failure to respond to 0.5–1.0 L plasmalyte bolus
The Ottawa Hospital	• Clear evidence of bleed<br>• Evidence of shock/trauma<br>• Anticipated need for massive transfusion<br>• Anti-coagulated patient with bleed
Hamilton General Hospital	• MD discretion
London Health Sciences Centre	• Severe/uncontrolled bleeding

ED = emergency department; MHP = massive hemorrhage protocol; RBC = red blood cells.

Appendix 2. Definition of MHP Packs Collected During Protocol Development at Each Potential Study Site for the Intervention Group

Site	MHP pack 1 (Intervention group only)			MHP pack 2 (Intervention group only)			MHP pack 3 (both groups)		
	RBCs (U)	PCC (IU) + FC (g)	Platelets (dose)*	RBCs (U)	PCC (U) + FC (g)	Platelets (dose)*	RBCs (U)	FP (U)	Platelets (dose)*
Sunnybrook Health Sciences Centre	4	2000 + 4	-	4	2000 + 4	1	4	2	0.5 in every other pack
Saint Michael's Hospital	4	2000 + 4	-	4	2000 + 4	1	4	4	-
Montreal General Hospital	4	2000 + 4	-	4	2000 + 4	1	5	4	1
Foothills Medical Centre	4	2000 + 4	-	4	2000 + 4	1	6	4	1
Vancouver General Hospital	4	2000 + 4	-	4	2000 + 4	1	6	6	1
The Ottawa Hospital	4	2000 + 4	-	4	2000 + 4	1	6	6	1
Hamilton General Hospital	4	2000 + 4	-	4	2000 + 4	1	4	2	1
London Health Sciences Centre	4	2000 + 4	-	4	2000 + 4	1	4	4	1

* 1 dose of platelets = 4 U of pooled or single donor platelets.
FC = fibrinogen concentrate; FP = frozen plasma; MHP = massive hemorrhage protocol; PCC = prothrombin complex concentrate; RBC = red blood cells.

Appendix 3. Definition of MHP Packs Collected During Protocol Development at Each Potential Study Site, Based on the Standard of Care

Site	MHP pack 1 (Control group only)			MHP pack 2 (Control group only)			MHP pack 3 (both groups)		
	RBCs (U)	FP (U)	Platelets (dose)*	RBCs (U)	FP (U)	Platelets (dose)*	RBCs (U)	FP (U)	Platelets (dose)*
Sunnybrook Health Sciences Centre	4	4	-	4	4	1	4	2	0.5 in every other pack
Saint Michael's Hospital	4	4	-	4	4	1	4	4	-
Montreal General Hospital	4	4	-	4	4	1	5	4	1
Foothills Medical Centre	4	4	-	4	4	1	6	4	1
Vancouver General Hospital	4	4	-	4	4	1	6	6	1
The Ottawa Hospital	4	4	-	4	4	1	6	6	1
Hamilton General Hospital	4	4	-	4	4	1	4	2	1
London Health Sciences Centre	4	4	-	4	4	1	4	4	1

FP = frozen plasma; MHP = massive hemorrhage protocol; RBC = red blood cells.
* 1 dose of platelets = 4 U of pooled or single donor platelets.

Appendix 4. Thromboelastometry-Guided Parameters for Targeted Therapy at Each Study Site

Participating site	Thromboelastometry parameter (target)	Therapeutic intervention
Sunnybrook Health Sciences Centre	ROTEM in OR phase of care:<br>• CT <90 s<br>• EXTEM >35 mm<br>• FIBTEM >8 mm	 • 2–4 U FP<br>• 1 dose platelets*<br>• 4 g FC
Saint Michael's Hospital	ROTEM in the laboratory:<br>• EXTEM CT >80 s<br>• EXTEM A10 or MCF >35 mm + abnormal FIBTEM A10 or MCF >7 mm	 • Consider FP<br>• Consider FC
Montreal General Hospital	N/A	N/A
Foothills Medical Centre	N/A	N/A
Vancouver General Hospital	ROTEM in the trauma bay/ED:<br>• EXTEM A10 ≤40 mm (or CFT >130 s) and FIBTEM <10 mm<br>• EXTEM A10 ≤40 mm (or CFT >130 s) and FIBTEM >10 mm<br>• EXTEM CT ≥100 s<br>• EXTEM ML >10%	 • 4 g FC<br>• 1 dose platelets*<br>• 2–4 U FP<br>• 2 g TXA
The Ottawa Hospital	N/A	N/A
Hamilton General Hospital	N/A	N/A
London Health Sciences Centre	N/A	N/A

* 1 dose = 4 U of pooled or single donor platelets.
A10 = amplitude at 10 minutes; CFT = clot formation time; CT = clotting time; FC = fibrinogen concentrate; FP = frozen plasma; MCF = maximum clot firmness; ML = maximum lysis; OR = operating room; TXA = tranexamic acid.

Appendix 5. Laboratory Test-Guided Parameters for Targeted Therapy at Each Study Site

Participating site	Laboratory parameter	Therapeutic intervention
Sunnybrook Health Sciences Centre	• INR <1.5<br>• Fib >2.0 g/L<br>• Platelets >100 x 10^9/L	• 2–4 U FP<br>• 4 g FC<br>• 1 dose platelets
Saint Michael's Hospital	• Hgb >80<br>• INR <1.5<br>• Fib >1.5–2 g/L<br>• Platelets >50 x 10^9/L if active hemorrhage<br>• Platelets >100 x 10^9/L for CNS bleed	• RBC as needed<br>• FP as ordered by MD<br>• 4 g FC<br>• 1 dose platelets<br>• 1 dose platelets
Montreal General Hospital	• At MD discretion	• N/A
Foothills Medical Centre	• Hgb ≥100<br>• INR <1.5<br>• Fib >1.5–2 g/L<br>• Platelets >100 x 10^9/L	• RBC as needed<br>• 2–4 U FP<br>• 2–4 g FC<br>• 1–2 U platelets
Vancouver General Hospital	• Hgb >100<br>• INR <1.5<br>• Fib >2.0 g/L<br>• Platelets >80 x 10^9/L	• RBC as needed<br>• 3–4 U FP (15 mL/kg)<br>• 4 g FC<br>• 1 dose platelets
The Ottawa Hospital	• At MD discretion	• N/A
Hamilton General Hospital	• At MD discretion	• N/A
London Health Sciences Centre	• Hgb >70<br>• INR <1.5<br>• Fib >1.5 g/L<br>• Platelets >50 x 10^9/L	• RBC as needed<br>• 2–4 U FP<br>• 4 g FC<br>• 1 dose platelets

A10 = amplitude at 10 minutes; CFT = clot formation time; CT = clotting time; FC = fibrinogen concentrate; Fib = plasma fibrinogen level; FP = frozen plasma; Hgb = hemoglobin; INR = international normalized ratio; MCF = maximum clot firmness; ML = maximum lysis; OR = operating room; TXA = tranexamic acid.
1 dose of platelets = 4 U of pooled or single donor platelets.

Appendix 6. CONSENTING PROCESS GUIDANCE DOCUMENT

The following processes are to be followed for obtaining consent from SDM and patient

Patient alive + not critically ill	Patient alive + critically ill	Patient expires
↓	↓	↓
Follow-up with patient (if competent) or SDM (if patient not competent) 24 hours after ER arrival to notify them of patient's enrollment in the study and to obtain consent. If patient has not recovered, obtain consent from SDM and continue to follow-up with the patient q4-7 days until they are competent to provide consent up to 28 days post admission. .	Follow-up with SDM 24 hours after trauma bay/ED arrival to notify them of patient's enrollment in the study. If SDM is comfortable, obtain consent. Follow-up with patient after they have recovered. If additional time is needed follow up q4-7 days until 28 days post admission. If patient expires before becoming competent, follow 'patient expires' path.	Follow-up with SDM at 14-21 days after expiry to notify them of patient's enrollment in study. If SDM is comfortable, obtain consent. If SDM is not ready to decide but is agreeable to further contact, make one additional attempt at 28 days post.

<u>**Consent Scenarios**</u>		<u>**Actions**</u>
SDM consents + Patient consents	→	Collect all study data
SDM consents + Patient not capable	→	Collect all study data
SDM consents + Patient does not consent	→	Document treatment allocation only; collect no additional data; patient not included in analysis
SDM not approachable + Patient consents	→	Collect all study data
SDM not approachable + Patient does not consent	→	Document treatment allocation only; collect no additional data; patient not included in analysis
SDM not approachable + Patient not capable or dead	→	Collect all study data
SDM does not consent + Patient consents	→	Collect all study data
SDM does not consent + Patient does not consent	→	Document treatment allocation only; collect no additional data; patient not included in analysis
SDM does not consent + Patient not approachable	→	Document treatment allocation only; collect no additional data; patient not included in analysis
