# Supplementary material for: Factors in the Initial Resuscitation of Patients With Severe Trauma: The FiiRST-2 Randomized Clinical Trial
Source: JAMA Netw Open. 2025 Sep 22;8(9):e2532702. doi: 10.1001/jamanetworkopen.2025.32702 (PMC12455389; doi:10.1001/jamanetworkopen.2025.32702)
Supplement: Supplement 2. — Statistical Analysis Plan [file jamanetwopen-e2532702-s002.pdf]

Statistical Analysis Plan (SAP)

Sponsor:	Keyvan Karkouti, MD, Toronto General Hospital
Study Title:	Prospective, multi-center, randomized, parallel-control, superiority study comparing administration of clotting factor concentrates with a standard massive hemorrhage protocol in severely bleeding trauma patients
Protocol Version/Date:	Version 3.0, 11-Jun-2022
SAP Version/Date:	Version 1.0, 26-Apr-2023
Supersedes SAP Version:	Not applicable (initial version)
Appendices (external documents):	1. List of Tables, Listing, Figures (TLFs)

Approval

The Trial Statistician hereby confirms that the SAP was prepared in conformance with the procedures and principles set forth in the indicated protocol version and all established relevant guidelines.

Name<br>Affiliation, Function	Signature:	Date:
Marti Jones<br>Ergomed, Trial Statistician		

By signing hereafter, I confirm that this Statistical Analysis Plan adequately describes the statistical analyses to be performed in the context of this study.

Name<br>Affiliation, Function	Signature:	Date:
Keyvan Karkouti<br>Toronto General Hospital, Sponsor		

Revision history

SAP Version	Version date	Reason(s) for change
1	26-Apr-2023	Not applicable.

TABLE OF CONTENTS

LIST OF ABBREVIATIONS 4
1 Study information 5
1.1 Primary objective 5
1.2 Secondary objectives 5
1.3 Study design 5
1.4 Planned sample size 6
1.5 Independent data safety monitoring committee 6
2 General Information 7
2.1 Background details 7
2.2 Changes to protocol specified analyses 7
2.3 Individual protocol deviations 7
3 Analysis Populations 7
3.1 Intention to Treat Population 7
3.2 Modified Intention to Treat Population 8
3.3 Per-protocol Population 8
3.4 Subgroup analyses 8
4 Statistical Analyses 8
4.1 Conventions 9
4.1.1 Baseline definition 9
4.1.2 Visit Assignment 9
4.1.3 Missing data 9
4.1.4 Pooling of centres 9
4.2 Demographic and other background data 9
4.2.1 Basic description 9
4.3 IMP exposure, compliance 10
4.4 Concomitant medication and procedures 10
4.5 Efficacy 10
4.5.1 Primary endpoint 10
Sensitivity analyses for primary endpoint 10
4.5.2 Secondary endpoints 11
4.5.3 Additional efficacy endpoints 12
4.6 Safety 14
4.6.1 Multi-organ failure 15
4.6.2 Abdominal compartment syndrome and limb compartment syndrome 15
4.6.3 Transfusion reactions 15
4.6.4 Adverse events 15
4.6.5 Duration of ICU Stay 16
4.6.6 All-cause Mortality 16
4.6.7 Laboratory variables 16
4.7 Interim analysis 16
5 Quality Control 17
6 References 17
7 Standards used in Preparation of Statistical Outputs 18
7.1 Programming 18
7.2 Layout 18
7.3 Headers, Titles and Footnotes 18
7.4 General Conventions 19
Appendices 21
1. List of Tables, Listings, Figures 21

LIST OF ABBREVIATIONS

Abbreviation	Description
ABP	Allogeneic Blood Products
AE	Adverse Event
ANOVA	Analysis of Variance
BMI	Body Mass Index
BP	Blood Pressure
CI	Confidence Interval
CRF	Case Report Form
CSR	Clinical Study Report
DRM	Data Review Meeting
EAIR	Exposure-adjusted Incidence Rate
ED	Emergency Department
FAS	Full Analysis Set
FC	Fibrinogen Concentrate
FP	Frozen Plasma
GCP	Good Clinical Practice
ICH	International Council for Harmonisation
IDSMC	Independent Data Safety Monitoring Committee
ITT	Intention-to-treat
LSmeans	Least Square Means
MedDRA	Medical Dictionary for Regulatory Activities
MHP	Massive Hemorrhage Protocol
mITT	Modified Intention-to-Treat
PCC	Prothrombin Complex Concentrate
PP	Per-protocol
PT	Preferred Term
RBC	Red Blood Cell
REB	Research Ethics Board
SAE	Serious Adverse Event
SAF	Safety Analysis Set
SAP	Statistical Analysis Plan
SDM	Substitute Decision Maker
SOC	System Organ Class
SOP	Standard Operating Procedure
TEAE	Treatment Emergent Adverse Event
TLFs	Tables, Listings, Figures
TS	Trial Statistician

1 STUDY INFORMATION

This document describes the rules and conventions to be used in the presentation and analysis of efficacy and safety data from the FiiRST-2 protocol. It describes the data to be summarized and analyzed, including specifics of the statistical analyses to be performed. This statistical analysis plan (SAP) is based on protocol version 3.0, dated 11 Jun 2022.

1.1 Primary objective

The primary objective of this study is to determine the impact of early co-administration of fibrinogen concentrate (FC) and prothrombin complex concentrate (PCC) on the total number of allogeneic blood products (ABPs) transfused compared to the current standard of care (frozen plasma [FP] administered in a 1:1 ratio-based plasma resuscitation).

1.2 Secondary objectives

- To compare the hemostatic efficacy of the intervention with the standard of care, as measured by transfusion of ABPs, use of hemostatic interventions, and correction of coagulopathy based on laboratory tests.
- To assess the safety of the intervention, focusing on arterial and venous thromboembolic complications.

1.3 Study design

FiiRST-2 is a multicenter, randomized, controlled, superiority trial, utilizing a conventional, parallel group, two-armed, with an adaptive two-stage design, performed at six Level 1 Trauma Centers in Canada. The study is designed to examine the effect on number of ABP units of early replacement of fibrinogen and clotting factors via FC and PCC in trauma patients with severe hemorrhage versus the current standard of care (ratio-based plasma resuscitation (ratio 1:1), and FC administered in response to low fibrinogen levels).

Upon arrival at the trauma bay/emergency department (ED) following the activation of the massive hemorrhage protocol (MHP) according to the MHP activation criteria at each study site (within the first hour post-arrival), patients will be enrolled. Once eligibility is confirmed, the blood bank medical laboratory technologist will randomize the patient to one of two groups: the intervention group, who will receive FC and PCC or the control group who will receive ratio-based plasma resuscitation with FP (1:1) and FC administered in response to low fibrinogen levels, as per the standard of care at each study site.

Patients in both groups will receive MHP treatment packs as indicated by the study site MHP initiation criteria. The first two packs will contain therapeutics according to the group assignment: patients in the intervention group will receive FC (Fibryga) and PCC (Octaplex) in the first and second packs and patients in the control group will receive FP in the first and second packs. In both groups, red blood cell (RBCs) will be included as part of the first and second MHP packs, and 1 dose of platelets will also be included as part of the second MHP pack. Both RBCs and platelets will be administered according to the clinical situation (initially as part of the ratio-based resuscitation, then guided by lab results). In the control group, FC may be administered if hypofibrinogenemia (fibrinogen level below 1.5–2.0 g/L or FIBTEM A10 below 8–12 mm) is identified as part of routine testing, at the discretion of the clinical team. The maximum time frame for administration of the second MHP pack (if required), is 24 hours from arrival at the trauma bay/ED or termination of the MHP (whichever comes first).

If a third pack is required, and thereafter, patients in both groups will receive MHP packs according to MHP guidelines at each participating site (but with a minimum of a 2:1 ratio of RBC to plasma until goal-directed transfusion is possible) or revert to a laboratory or visco-elastic-guided transfusion as per the local guidelines if hemorrhage control is achieved and the MHP is terminated. The MHP should be terminated once bleeding is controlled and the hospital MHP termination criteria are met. Thereafter, transfusions should be based on the results of laboratory testing and rate of bleeding.

The primary efficacy outcome will be the number of ABP units (RBCs, FP and platelets) administered during the first 24 hours following arrival at the trauma bay/ED, with the primary comparisons being in the modified intention-to-treat (mITT) population.

Safety outcomes will be measured for the first 28 days following arrival at the trauma unit, which is the duration of participation of each patient in the trial.

1.4 Planned sample size

The statistical analysis of the primary outcome, a composite number of units of ABPs (RBC + FP + platelets) transfused within 24 hours post trauma bay/ED arrival, will be based on the mean number of ABPs within the first 24 hours. To demonstrate that the early administration of FC + PCC is clinically superior to the usual component therapy (FC administration depending on fibrinogen levels and/or clinician discretion and clotting factor replacement with FP), with respect to the mean number of ABPs, a two-sample, one-sided test of the pair of hypotheses: H_0: $RR \geq RR_0$ vs. H_a: $RR < RR_0$ will be carried out with an overall type I error probability of $\alpha = 0.025$. Here, λ_1 and λ_2 denote the mean number of ABPs (RBC + FP + platelets) in the control group (standard of care) and intervention groups, respectively. RR is the ratio λ_2/λ_1 and RR_0 will be set equal to 1.0 to test for superiority.

A mean difference in 5 units of the composite outcome (mean 15 units in the control group and mean 10 units in the intervention group) is considered as a clinically meaningful difference that should be detected with at least 80% power. Sample size estimations based on these assumptions were performed with the software nQuery (version 8.3). Empirical estimates of the mean number of allogeneic blood products units within the first 24 hours and its dispersion were based on results of the FiiRST-1 Study [1] with the same endpoint in the same indication and similar treatment. A sample size of 297 patients would suffice to demonstrate the superiority of the investigational treatment under the stated assumptions. The FiiRST-1 study had a 10% patient drop-off (exclusions post randomization). Hence, we will inflate our sample size to account for a drop-out percentage of up to 15%. For this reason, the study plans to enroll up to 350 patients.

A planned unblinded interim analysis will be performed after about 120 patients have completed the study. This interim analysis will calculate the conditional power of the primary analysis test statistic and perform a sample size re-assessment if the conditional power is in the promising zone. The sample size will be increased to achieve the targeted conditional power of 80% given the interim results. Hence, the final number of enrolled patients will depend on the sample size re-calculation.

1.5 Independent data safety monitoring committee

An independent data safety monitoring committee (IDSMC) will be established by the Sponsor. The IDSMC will be composed of recognized experts in the field of emergency medicine,

critical care medicine, trauma surgery, methodology/epidemiology, transfusion medicine/hematology, and anesthesiology. To identify any safety concerns with the conduct of the trial, the IDSMC will review study outcomes after 60 patients have been enrolled, at the interim analysis and then every 100 patients thereafter. In particular, data on deaths, serious adverse events (SAEs) and thromboembolic events will be reviewed in detail. The IDSMC will review data at the adaptive interim analysis after 120 patients have completed the study, at which point it will give advice on the continuation, modification, or termination of the study. A written study-specific charter will define in detail the composition, responsibilities, and procedures of the IDSMC.

2 GENERAL INFORMATION

2.1 Background details

All study data will be transferred to a SAS database (version 9.4 or later) for statistical analysis purposes. Data will be imported from a Data Capture System (OPVerdi) via validated SAS programs.

The SAP will be finalized before database lock and unblinding, after agreement with the Sponsor on patient disposition and coding.

2.2 Changes to protocol specified analyses

The protocol defines a restricted safety population, defined as patients who receive at least one whole or parts of their first randomized MHP pack who did not consent participation post-randomization, but this population will not be used for any analyses. This analysis population is not relevant since only randomization results and inclusion/exclusion criteria were entered in the database for patients who refused to provide consent for the study, as per research ethics board allowances.

2.3 Individual protocol deviations

A detailed review of all documented and derived deviations from protocol will be part of the blinded data review meeting (DRM) before database lock. During this DRM the impact of protocol deviations on the analysis will be assessed and the conclusions recorded. The list of patients excluded from any analysis sets due to protocol deviations will be finalized prior to lock.

3 ANALYSIS POPULATIONS

In general, the disposition of patients will be displayed for the patient populations defined below.

3.1 Intention to Treat Population

All randomized patients who receive at least a whole or parts of their first randomized MHP pack (not including the RBCs) and agree to remain in the study after consenting, or for whom patient/substitute decision maker (SDM) consent could not be obtained but local research ethics board (REB) approval to use the data is obtained. Should a patient receive MHP packs that are not in concordance with the randomization schedule, the treatment group will be defined according to the randomization (rather than the actual treatment received).

3.2 Modified Intention to Treat Population

All randomized patients who receive any of the non-RBC, non-platelet products in MHP pack 1 or beyond of the intended first-line treatment and agree to remain in the study after consenting, or for whom patient/SDM consent could not be obtained but local REB approval to use the data is obtained. Should a patient receive treatment that is not in concordance with the randomization schedule, the treatment group will be defined according to the actual treatment received (rather than the randomization). If no randomization errors are observed the ITT population will be identical to the mITT population.

The mITT analysis population is considered the primary population for analysis of the primary endpoint.

3.3 Per-protocol Population

The per-protocol (PP) population includes all patients in the ITT population who received a minimum amount of treatment (at least 2 g of FC and 1000 IU of PCC or 2 units of FP) and did not have a major protocol deviation that could impact the analysis of the primary endpoint. Protocol deviations, including violations of inclusion/exclusion criteria, will be reviewed prior to database lock and the list of subjects to be excluded will be finalized prior to lock and unblinding of the study.

The evaluation of the primary endpoint will additionally be performed for the PP population.

3.4 Subgroup analyses

The following subgroups for statistical analysis of the primary endpoint were pre-defined:

- Patients with blunt versus penetrating trauma (excluding patients with trauma resulting from both mechanisms)
- Patients with versus those without traumatic brain injury (head AIS score ≥ 3 vs. < 3)
- Patients less than 60 years of age versus those 60 years of age and older
- Patients massively transfused (10 RBC U or more) versus those not massively transfused
- Patients at the largest volume enrolling site vs. patients at all other sites
- Male vs. female
- Patients who survived at least 6 hours past arrival at trauma bay/ED
- Patients who survived at least 24 hours past arrival at trauma bay/ED

4 STATISTICAL ANALYSES

All statistical analyses will be performed using the SAS® software (Version 9.4 or later).

Descriptive statistics will be given by treatment group and overall where appropriate.

If not stated otherwise the following standard types of descriptive analyses will be presented:

– Descriptive statistics for continuous data

  N, mean, standard deviation (SD), min, lower quartile, median, upper quartile and max will be presented. These descriptive statistics will be determined for measured values and optionally for differences to baseline.

– Descriptive statistics for categorical data

Absolute frequencies and percentages will be presented. Percentage bases (denominators) will be identified in the table title or footnote (i.e., all patients at risk, all non-missing cases, all cases).

- Inferential statistics

  Unless otherwise stated, all statistical tests will be performed two-sided and at a type I error probability of α=0.05.

  Unless otherwise stated, all confidence intervals (CIs) will be derived two-sided and at a confidence probability of 1-α=0.95.

- Listings

  All recorded data will be listed by patient (sorted by treatment and patient ID).

Derived data will be stored in special analysis data sets and will be calculated as outlined in section 6.1.

4.1 Conventions

4.1.1 Baseline definition

Study baseline is defined as the last non-missing measurement taken prior to the start date/time of the first pack of the MHP.

4.1.2 Visit Assignment

For summaries and analyses of laboratory results and SOFA scores, results will be assigned to a study day relative to time of arrival at the trauma bay/ED. Results > 0 to ≤ 24 hours will be assigned to Day 1, results >24 to ≤ 48 hours will be assigned to Day 2, etc.

4.1.3 Missing data

No imputation of missing data is planned. To address the possible issue of survivor bias on the primary endpoint (patients who survive longer may receive more ABPs), sensitivity analyses will be conducted in the subset of patients who survive for at least 6 hours past arrival at the trauma bay/ED and in the subset of patients who survive for at least 24 hours post arrival at the trauma bay/ED.

4.1.4 Pooling of centres

No pooling of centres will be performed.

4.2 Demographic and other background data

4.2.1 Basic description

The disposition of patients will be tabulated by treatment and for the entire population. Details on protocol deviations will be listed.

Discontinued patients will be described by frequency distributions including the reasons and in individual listings.

Demographic data (sex, age, race, height, weight, and body mass index (BMI)) will be summarized in tables and presented for the mITT and PP populations.

The following injury related baseline data will also be summarized for the mITT and PP populations:

- Time from injury to admission (in hours)

- Mechanism of injury
- Injury severity score, including abbreviated injury scores for head, face, neck, thorax, abdomen, spine, upper extremity, lower extremity, and external
- Glasgow coma score

Demographic and injury data will also be listed.

Comorbidities and other relevant medical history data will be listed.

4.3 IMP exposure, compliance

The number and percentage of patients receiving each IMP pack, along with the number and percentage of patients receiving full vs. partial IMP and the reasons for not receiving the full administration will be summarized.

Details of IMP exposure will be listed.

4.4 Concomitant medication and procedures

Concomitant medications administered to treat SAEs will be listed.

Surgical or interventional procedures done during the study will also be listed.

4.5 Efficacy

4.5.1 Primary endpoint

The primary endpoint of the study is the composite number of all ABP units (RBCs, FP and platelets; including FP as IMP) transfused within 24 hours of arrival at the trauma bay/ED. The mITT will be used for the primary endpoint analysis.

The number of allogeneic units will be calculated as:

- Platelets: 1 apheresis unit = 4 allogeneic units, 1 non-apheresis unit = 4 allogeneic units
- FP: 1 apheresis 500 mL unit = 2 allogeneic units, 1 non-apheresis unit or 1 apheresis 250 mL unit = 1 allogeneic unit
- RBC: 1 unit is counted as 1 allogeneic unit.

Descriptive statistics and boxplots for the number of ABP units will be presented.

The primary analysis will test the hypotheses detailed in section 1.4.

Testing of the hypotheses will be performed using a generalized linear model for count data with log-link function and a negative binomial error term, with treatment group as main effect. Inferences will be based on the one-sided 97.5% CI for the ratio λ_2 / λ_1 derived from the estimated least square means (LSmeans) of this model. Superiority will be concluded if the upper limit of this CI is strictly less than $R_0 = 1.0$ (i.e., the mean number of ABPs is larger in the SoC group).

Sensitivity analyses for primary endpoint

The following sensitivity analyses for the primary endpoint will be conducted:

- Primary endpoint analyzed in the PP population.
- Primary endpoint calculated as the total number of ABP units, excluding FP as IMP.
- Primary endpoint analyzed in the subset of patients who survived for at least 6 hours past arrival at the trauma bay/ED.

- Primary endpoint analyzed in the subset of patients who survived for at least 24 hours past arrival at the trauma bay/ED.

4.5.2 Secondary endpoints

The secondary endpoints will be analyzed in the mITT population. To prevent an inflated probability of a Type I error in the testing of the secondary endpoints due to multiple testing, the Holm-Bonferroni method will be used. The p-values for the following tests will be ordered from smallest to largest: the total number of RBCs transfused within the first 24 hours, the incidence of experiencing any thromboembolic event, and the number of ventilator-free days. These ordered p-values will be tested at the following significance levels: 0.017, 0.025, 0.05. The testing procedure stops when the first test fails to reject the null hypothesis.

The full list of secondary endpoints includes:

- Total number of units of RBCs transfused within the first 24 hours following arrival at the trauma bay/ED
- Incidence of thromboembolic events, as defined by evidence of any of the following, from arrival at the trauma bay/ED, up to 28 days:
  - Deep vein thrombosis
  - Pulmonary embolism
  - Myocardial infarction
  - Ischemic stroke
  - Arterial or venous thrombosis at other sites
- Ventilator-free days, defined as the number of days up to Day 28 following arrival at the trauma bay/ED on which a patient breathed without assistance (if period of unassisted breathing lasted at least 48 consecutive hours). Patients who die during study follow-up or require 28 or more days of mechanical ventilation will be assigned 0 ventilator-free days.

The total number of RBCs transfused within the first 24 hours following arrival at the trauma bay/ED will be summarized similarly to the primary endpoint. The analysis of the total number of RBCs will also be conducted in the subset of patients who survive for at least 6 hours past arrival at the trauma bay/ED and the subset of patients who survive for at least 24 hours past arrival at the trauma bay/ED. The total number of FP units (including FP received as IMP) and platelets will also be summarized.

Thromboembolic events will be identified as AEs coded to the following Medical Dictionary for Regulatory Activities (MedDRA) preferred terms:

- Deep vein thrombosis
- Pulmonary embolism
- Myocardial infarction
- Arterial thrombosis
- Venous thrombosis
- Ischemic stroke

The incidence of experiencing any thromboembolic event will be compared between the treatment groups using the p-value from an exact binomial test. The risk difference between the treatment groups and corresponding 95% CI will also be presented.

The time to first thromboembolic event will also be compared in the 2 treatment groups using a Kaplan-Meir analysis. The time from first administration of IMP to the start of the first thromboembolic event will be calculated for each patient. Patients who do not experience a thromboembolic event will be censored at the last visit date or death date for patients who die without experiencing an event. Time to first thromboembolic event will be summarized using Kaplan-Meier quartile estimates and the corresponding 95% confidence intervals. The primary comparison of the treatment effect will be based on the log-rank test.

The exposure-adjusted incidence rate (EAIR) of thromboembolic events will also be explored. The EAIR per 100 patient-days (p-d) is the number of patients who experience an event divided by the total patient exposure time (in days) multiplied by 100. For patients who experience an event, the exposure time is from first administration of IMP to time of the first thromboembolic event. For patients who do not experience an event, the exposure time is from first administration of IMP to the minimum of study completion date and death date. Exposure-adjusted incidence rates will be calculated by treatment group for any thromboembolic event and separately for each thromboembolic event preferred term.

Ventilator-free days will be calculated as:

- For patients who die, ventilator-free days = 0
- For patients who require 28 or more days of mechanical ventilation, ventilator-free days =0
- For patients who complete the 28 days of follow-up, ventilator-free days = 28 – sum of all time periods (in days) patient is receiving ventilatory support. If the end date and time of ventilatory support is missing, patient will be assumed to have been on ventilatory support until the study completion date.

The number of ventilator-free days will be compared using the Wilcoxon rank-sum test.

4.5.3 Additional efficacy endpoints

Results for the additional efficacy endpoints are considered exploratory and no adjustments for multiplicity will be made. The following additional efficacy endpoints will be analyzed in the mITT population:

- Total and individual numbers of units and volumes (liters) of ABPs (RBCs, FP [excluding FP as IMP] and platelets) transfused within 6 hours, 24 hours and within 7 days post arrival at the trauma bay/ED
- Total volume of crystalloids and other colloids administered within the first 6 and 24 hours following arrival at the trauma bay/ED
- Rescue use of hemostatic agents (fibrinogen concentrate, PCC, and rFVIIa) within the first 24 hours following arrival at the trauma bay/ED
- Laboratory endpoints upon arrival (before drug administration), if measured, and following infusion of the investigational medicinal products (IMPs), as per each site protocol routine, measured within the first 24 hours and within 7 days following arrival at the trauma bay/ED:
    - Plasma fibrinogen levels

    - International normalized ratio (INR), prothrombin time and activated partial thromboplastin time (aPTT)
    - Hemoglobin and hematocrit levels
    - Platelet count
    - Base deficit, pH and lactate
    - Thromboelastometry values: EXTEM clotting time (CT), EXTEM A10, EXTEM maximum clot firmness (MCF), FIBTEM A10, FIBTEM MCF, and EXTEM LY30
- Days out of hospital within the first 28 days following arrival at the trauma bay/ED
- Time to death over the first 28 days following arrival at the trauma bay/ED

The total and individual number of units of ABPs (RBCs, FP and platelets) transfused will be summarized similarly to the primary endpoint.

The analysis of the volume of ABPs will be based on the derivation of the total volume in ml per unit, using the following median volumes:

- RBC unit = 300 mL
- FP unit = 290 mL
- Platelet unit = 350 mL.

The total and individual volume of ABPs transfused within 6 hours/24 hours/7 days of arrival at the trauma bay/ED will be summarized descriptively. An analysis of variance (ANOVA) will be used to compare the mean volumes between the 2 treatment groups. Nonparametric tests may be used if the assumptions of an ANOVA are not met.

The total volume (in mL) over all crystalloid and colloid products administered will be calculated within 6 hours and within 24 hours of arrival at the trauma bay/ED. The total volume will be summarized descriptively and compared between treatments groups using ANOVA. Nonparametric tests may be used if the assumptions of an ANOVA are not met.

The incidence of rescue use of hemostatic agents within the first 24 hours following arrival at the trauma bay/ED will be compared between the treatment groups using a logistic regression model. The odds ratio (OR) for rescue use of hemostatic agents (intervention vs. standard of care) will be presented. The comparison will be done separately for each type of product: Factor VIIa, fibrinogen, and PCC. The total dose received will also be summarized for each product. The analysis of incidence of rescue use of hemostatic agents will also be conducted in the subset of patients who survive for at least 6 hours after arrival at the trauma bay/ED and the subset of patients who survive for at least 24 hours after arrival at the trauma bay/ED.

The incidence of use of tranexamic acid and other hemostatic agents will also be summarized, along with the total dose of tranexamic acid.

For each laboratory endpoint, the following values will be summarized descriptively:

- Baseline
- Highest/lowest post-baseline value in the first 6 hours after first IMP administration

- Highest/lowest post-baseline value between 6-24 hours after first IMP administration
- Most extreme value per day for Day 1 – Day 7, where most extreme value is defined as
    - highest value for INR, prothrombin time, aPTT, base deficit, lactate, EXTEM clotting time, EXTEM LY30
    - lowest value for plasma fibrinogen, hemoglobin, hematocrit, platelet, pH, EXTEM A10, EXTEM MCF, FIBTEM A10, FIBTEM MCF

Due to differences in normal ranges by sex, summaries of hemoglobin and hematocrit will be summarized by sex as well as overall. Box plots will also be presented for the most extreme value for Days 1-7.

The number of days out of hospital within the first 28 days following arrival at the trauma bay/ED will be compared between treatments using the Wilcoxon rank-sum test. Patients who die during study follow-up or require 28 or more days of hospitalization will be assigned 0 days out of hospital.

Time to death over the first 28 days following arrival at the trauma bay/ED will be summarized using Kaplan-Meier quartile estimates and the corresponding 95% confidence intervals. Patients who are alive at the end of study will be censored on their last contact date. The primary comparison of the treatment effect will be based on the log-rank test. The estimate of the hazard ratio for treatment and the corresponding 95% CI from a Cox proportional hazards regression model will also be presented.

4.6 Safety

The following safety endpoints will be summarized in the modified ITT population:

- All documented adverse events (AEs) and serious adverse events (SAEs) during the first 28 days following arrival, including:
    - Multi organ failure (MOF) as measured by the Sequential Organ Failure Assessment (SOFA) score daily during ICU stay for up to 28 days following arrival at the trauma bay/ED
        - Highest SOFA score and time of highest SOFA score
        - Change in SOFA score
    - Incidence of abdominal compartment syndrome (ACS), defined as sustained intra-abdominal pressure >20 mmHg with or without an abdominal perfusion pressure (APP) of < 60 mmHg, that is associated with new organ dysfunction/failure
    - Incidence of limb compartment syndrome (LCS)
    - Incidence of transfusion reactions as defined by the International Society of Blood Transfusion
    - Incidence of TEAEs
- Duration of ICU stay

- 28-day all-cause mortality

4.6.1 Multi-organ failure

The highest SOFA score on Days 1-7, Day 14, Day 21, and Day 28 will be summarized descriptively. The time from trauma bay/ED admission to the highest SOFA score measured during the ICU stay will also be summarized. Patients who die will be assigned the maximum SOFA score of 24 for each analysis time point after their death date. The last observation carried forward (LOCF) approach will be implemented for patients with missing SOFA score due to other reasons (for example, not all SOFA components were measured). The last observed SOFA score will be carried through to each analysis visit with a missing result up to Day 28 or death.

Box plots of the highest score on Days 1-7, Day 14, Day 21, and Day 28 will also be presented.

4.6.2 Abdominal compartment syndrome and limb compartment syndrome

The incidence of ACS and LCS will be summarized.

4.6.3 Transfusion reactions

Transfusion reactions will be recorded as AEs. The incidence of transfusion reactions by SOC and preferred term will be summarized. Transfusion reactions will be identified by the following MedDRA preferred terms:

- Transfusion-related acute lung injury
- Transfusion-associated circulatory overload
- Congestive heart failure
- Transfusion-associated dyspnea
- Alloimmunization
- Hyperkalemia
- Hypocalcemia
- Urticarial reaction
- Anaphylaxis
- Febrile non-hemolytic transfusion reaction

4.6.4 Adverse events

Adverse events will be presented by MedDRA System Organ Class (SOC) and Primary Term (PT). It is assumed that coding of verbatim terms has been performed either by Ergomed or the Sponsor and that all relevant codes are stored in the database.

Summaries will include treatment-emergent AEs, defined as events that started or worsened after start of infusion with IMP. Summaries will be by SOC and PT, with PTs sorted according to the Internationally Agreed Sorting Order (MedDRA). Multiple counts within a PT or SOC (repeated or different included terms or changes in descriptors) will be counted only once for the calculation of incidences.

Incidence tables, i.e., frequency tables of patients experiencing at least one occasion of the event while at risk (along with the number of different occurrences of the TEAE), will be presented for the following types of adverse events:

- All TEAEs irrespective of the causality assessment

- TEAEs related to IMP (i.e., causality of possible or probable)
- Serious TEAEs (SAEs)
- TEAEs leading to death
- TEAEs leading to withdrawal of IMP

Listings will be provided for the following:

- All SAEs
- Treatment-emergent thromboembolic AEs
- TEAEs leading to withdrawal of IMP
- AEs leading to death

4.6.5 Duration of ICU Stay

The duration of the ICU stay is calculated as the sum of all ICU admission periods, where the duration of each period is calculated as the time from initial ICU admission or readmission to ICU discharge date. The duration of ICU stay will be summarized descriptively.

4.6.6 All-cause Mortality

All-cause mortality will be analyzed for the ITT and PP populations. Incidence of all-cause mortality will be compared between the 2 treatment groups using the Mantel-Haenszel risk ratio and corresponding 95% CI.

4.6.7 Laboratory variables

The safety assessment will include summaries of the following laboratory parameters: bilirubin, creatinine, albumin, alanine transaminase (ALT), alkaline phosphatase (ALP), aspartate aminotransferase (AST), troponin T-hs, calcium (ionized), and bicarbonate.

Descriptive summaries will summarize the most extreme value per day for Days 1 – 7, where the most extreme result is defined as:

- Highest value for bilirubin, creatinine, ALT, ALP, AST, troponin T-hs, calcium (ionized)
- Lowest value for albumin, bicarbonate

Box plots of the most extreme value for Days 1-7 will also be presented for each parameter. A listing of all abnormal (out of range) values will be presented.

4.7 Interim analysis

Due to the inherent variability in the primary endpoint and a yet substantial uncertainty about the effect size, an adaptive design approach will be used. For this, a planned interim analysis will be performed after 120 patients have completed the study. Primary aim of this interim analysis is to calculate the p-value and conditional power of the test statistic and perform a sample size re-assessment. This will be done in an unblinded interim analysis performed by an independent statistician who will report the results only to the independent data safety monitoring committee (IDSMC) which will make recommendations to the sponsor without revealing the treatment groups.

The study design will follow a group sequential design with O'Brien-Fleming error-spending function, a futility boundary and sample size re-estimation based on conditional power. Hence, the recommendation of the IDSMC can include:

- To continue the trial as planned until 350 patients have completed the study,
- To stop the trial for demonstrated superiority at the interim analysis,
- To stop the trial at the interim for futility or for requiring an increase in sample size that is considered unfeasible,
- To continue the trial with a modified sample size.

5 QUALITY CONTROL

The SAP was reviewed by the TS before signature. Particularly the TS has checked the consistency of the described methods and outputs with the actual version of the study protocol. In addition, a sponsor representative and an independent statistician have reviewed the SAP before final approval.

Log files of all SAS® programs used in the analysis will be checked for errors, warnings and suspicious notes by the statistical programmer. All findings will be either eliminated or commented upon. The final version of each program will be stored along with its log file in the electronic archive.

All programs will be validated by the program author or an independent statistical programmer depending on the requested validation level selected in the List of TLFs form (FRM/BS/001.02) for a particular program.

The agreement of the program outputs with the SAP, their consistency and plausibility will be checked by the TS. Moreover, the TS will review the outputs regarding completeness, readability and comprehensibility.

The described process is associated with the 'normal' level of program validation. Additional levels of quality control can be specified in the List of TLFs (see Appendix, 1) for individual outputs.

6 REFERENCES

[1] Nascimento B, Callum J, Tien H, Peng H, Rizoli S, Karanicolas P, et al. Fibrinogen in the initial resuscitation of severe trauma (FiiRST): a randomized feasibility trial. Br J Anaesth. 2016;117(6):775- 82.

7 STANDARDS USED IN PREPARATION OF STATISTICAL OUTPUTS

The below conventions will be followed as agreed with the Sponsor.

7.1 Programming

- One SAS program should create only one output.
- One output file can contain different output types (e.g. descriptive and inferential).
- Individual output files will be created in MS Word format (Rich Text Format, RTF).
- Once delivered to the client, numbering of TLFs will not be altered, unless agreed with the client

7.2 Layout

- TLFs will be produced in landscape format
- TLFs will have a minimum 2 cm on every side
- TLFs will be produced using the Courier New font, size 8
- Section numbering of TLFs will follow ICH E3 guideline.
- Numbering of TLFs will follow the convention XXX-YY, where XXX stands for a (sub-)section number of the ICH E3 guideline and YY represents the sequence number of the output within the section. A dash ('-') will always be used to separate section numbers from output sequence numbers
- Titles and footnotes for figures will also be in Courier New font, size 8.
- Tables and listings will be in black and white (no colour), figures may include only colour that can be distinguished when printed on a grey-scale printer
- Text styles, such as bolding, italics, borders, shading, and superscripted and subscripted text, will be used sparingly in the TLFs
- The ANSI character set will be used in the TLFs. Certain subscripts and superscripts (e.g., m^2, AUC_{norm}) will be employed on a case-by-case basis.
- Mixed case will be used for all titles, footnotes, column headers, and programmer-supplied formats, unless they are derived directly from the data

7.3 Headers, Titles and Footnotes

- All output will have the following header at the top left of each page showing the study ID, the date of output generation and an internal pagination, where Y stands for the total number of pages in the pertaining output.

```
Study ID                                          ERGOMED, YYYY-MM-DD
Study description                                      Page [ X / Y ]
```

- Also, all TLFs will have the following footer, identifying the generating SAS program (XXX.SAS), a reference to the relevant patient listing and the date of the data snapshot:

```
SAS program: <XXX>.sas          Ref. list X.X.X-YY          Data status: YYYY-MM-DD
```

- Each TLF will bear a title which is repeated on each page of the output.
- The title at the top of the page will be horizontally cantered in bold font.
- A blank line will separate the title from the body of the output.
- The title will consist of an Output number, a descriptive title and a description of the presented analysis set (if applicable).
- The title will have the following general appearance:

```
Table / Figure / List XX.X.X-YY
   Descriptive Title line 1
   Descriptive Title line 2
(All patients in the FAS, N=nnn)
```

- Each new footnote should start on a new line, where possible.
- Preferably, footnotes should be left justified. When extending beyond a single line, a manual linefeed should be inserted to avoid meaning distortion.
- An automatic footnote '(continued)' will appear at the bottom of TLFs that extend over more than one page.

7.4 General Conventions

- For measured variables column headers should include the unit in their description
- The order of treatment arms in the TLFs will be consistent throughout the entire TLF presentation
- Alphanumeric values are preferably displayed left-justified;
- Dates are presented left-justified
- Integer numbers (e.g., counts) can be centered or right-aligned
- Numbers containing fractional portions will be decimal-aligned.
- Fractional numbers with absolute value less than 1 will carry a leading zero, i.e. 0.123 not .123.
- Units of measured or derived variables will be included where appropriate
- Unless otherwise warranted, the estimated mean, median and quartiles for a set of values will be displayed with 1 more significant digit than the original values, and standard deviations with 2 more significant digits. The minimum and maximum should report the same significant digits as the original values.

- P-values are output in the format: "0.xxxx", where xxxx is the value rounded to 4 decimal places. P-values less than 0.0001 will be presented as <0.0001.
- Precision of percentages displayed will depend on the total study size. For studies with less than 1000 patients values will be presented with one decimal place. For studies with more than 1000 patients, values will be presented with two decimal places.
- Tabular display of data for medical history, prior/concomitant medications and all tabular displays of adverse event data are generally presented by body system, treatment class, or SOC according to the Internationally Agreed Sorting Order of the MedDRA, unless otherwise agreed.
- The percentage of patients is normally calculated as a proportion of the number of patients assessed in the relevant treatment group (or overall) for the analysis (sub-) population presented.
- For categorical summaries (number and percentage of patients) where a patient can be included in more than one category, an explanatory text will be added to clarify that multiple answers were possible.
- Missing values will be displayed either by a double-dash ("--") or as "NA" (='not available/applicable'), whichever is appropriate.
- Dates are displayed in according to ISO date/time format as YYYY-MM-DD, e.g. 2010-03-24. Missing dates may be represented as "NA", if not available/applicable.
- Clock times are displayed as HH:MM or HH:MM:SS based on 24-hour clock

APPENDICES

1. List of Tables, Listings, Figures
