# Supplementary material for: Factors in the Initial Resuscitation of Patients With Severe Trauma: The FiiRST-2 Randomized Clinical Trial
Source: JAMA Netw Open. 2025 Sep 22;8(9):e2532702. doi: 10.1001/jamanetworkopen.2025.32702 (PMC12455389; doi:10.1001/jamanetworkopen.2025.32702)
Supplement: Supplement 3. — eTable 1. Massive Hemorrhage Protocol Activation Criteria at Each Participating Site eTable 2. Details on Compliance With the Administration of Intervention and Control eTable 3. Primary Outcome in Subgroups of Interest eTable 4. Trial Outcomes by Treatment Group in the Per Protocol Population eFigure 1. Transfusion of Blood Products Across the First 24 Hours Following Admission, mITT N=137 eFigure 2. Coagulation Profile and Hemoglobin Across the First 24 Hours Following Admission, mITT N=137 [file jamanetwopen-e2532702-s003.pdf]

Supplemental Online Content

da Luz LT, Karkouti K, Carroll J, et al. Factors in the initial resuscitation of severe trauma: the FiiRST-2 randomized clinical trial. *JAMA Netw Open*. 2025;8(9):e2532702. doi:10.1001/jamanetworkopen.2025.32702

eTable 1. Massive Hemorrhage Protocol Activation Criteria at Each Participating Site

eTable 2. Details on Compliance With the Administration of Intervention and Control

eTable 3. Primary Outcome in Subgroups of Interest

eTable 4. Trial Outcomes by Treatment Group in the Per Protocol Population

eFigure 1. Transfusion of Blood Products Across the First 24 Hours Following Admission, mITT N=137

eFigure 2. Coagulation Profile and Hemoglobin Across the First 24 Hours Following Admission, mITT N=137

This supplemental material has been provided by the authors to give readers additional information about their work.

© 2025 da Luz LT et al. *JAMA Network Open*

eTable 1. Massive hemorrhage protocol activation criteria at each participating site

Participating site	MHP activation criteria
Sunnybrook Health Sciences Centre	• Life-threatening bleeding situation requiring mobilization of blood bank, laboratory and clinical resources<br>• Anticipated need for at least 4 U of RBC immediately and component therapy (platelets, plasma and fibrinogen)<br>• Systolic blood pressure less than 90 mmHg and/or requiring inotropes
Saint Michael's Hospital	• Penetrating trauma AND persistent hypotension (2 measurements of systolic blood pressure <90 mmHg 5 min apart in the ED)<br>• Blunt trauma AND persistent hypotension AND suspected bleeding<br>• A recognized need for un-crossmatched RBC<br>• Known or suspected cardiac rupture, aortic rupture or atrial leak
Vancouver General Hospital	• Trauma patient with active hemorrhage AND systolic blood pressure <90 mmHg or impalpable radial pulse AND failure to respond to 0.5–1.0 L plasmalyte bolus
Kingston Health Sciences Centre	• Situations with a hemodynamically unstable patient with evidence of rapid (>150 mL/min) and/or massive (>1500mL) blood loss<br>• A Critical Activation Threshold (CAT1) of three or more units of RBC over an hour<br>• A Resuscitation Index (RI) of four or more 'units' of fluid (ie: 1 unit RBC, 1 unit FFP, 500mL colloid, or 1000mL crystalloid) within 30 minutes<br>• A Shock Index of 1.4 of higher (heart rate/systolic blood pressure)
Hamilton General Hospital	• MD discretion
London Health Sciences Centre	• Severe/uncontrolled bleeding

ED = emergency department; MHP = massive hemorrhage protocol; RBC = red blood cells.

eTable 2. Details on compliance with the administration of intervention and control

	Plasma (n = 71)		FC and PCC (n = 66)
**Treatment administration**			
**First pack**			
Complete dose, No. (%)	47 (66.2)		59 (89.4)
Partial dose, No. (%)	24 (33.8)		7 (10.6)
**Second pack**			
Complete dose, No. (%)	21 (29.6)		26 (39.4)
Partial dose, No. (%)	4 (5.6)		7 (10.6)
Not required, No. (%)	46 (64.8)		33 (50)
**Dosage of IMPs**	**Plasma, units**	**FC, grams**	**PCC, IU**
**First pack**			
Mean (SD)	3.3 (1.0)	3.9 (0.5)	1856.1 (494.3)
Median (IQR)	4 (2,4)	4 (4,4)	2000 (2000,2000)
**Second pack**			
Mean (SD)	3.7 (0.8)	3.7 (1.1)	1727.3 (674.2)
Median (IQR)	4 (4,4)	4 (4,4)	2000 (2000,2000)

Abbreviations: FC – fibrinogen concentrate, IQR – interquartile range, IU – international unit, PCC – prothrombin complex concentrate, SD – standard difference.

© 2025 da Luz LT et al. *JAMA Network Open*

eTable 3. Primary outcome in subgroups of interest

Characteristic	Plasma (n = 71)	FC and PCC (n = 66)	Total (n = 137)
Sex No. (%)			
Male, median (IQR)	11.5 (8 to 30.5)	12 (6 to 23)	12 (8 to 26)
Female, median (IQR)	13 (7 to 33)	10 (6 to 22)	12 (7 to 22)
Injury mechanism (No., %)			
Blunt, median (IQR)	13.5 (8 to 30)	10 (6 to 22)	11 (7 to 26)
Penetrating, median (IQR)	11 (7.5 to 25)	18 (5 to 23)	11 (6 to 23)
Brain injury, No. (%)			
Yes, median (IQR)	14 (8 to 32)	10 (6 to 25)	12 (7 to 28)
No, median (IQR)	11 (8 to 30)	12 (7 to 23)	11 (7 to 23)
Age, No. (%)			
< 60 years, median (IQR)	11 (8 to 35)	12 (6 to 23)	12 (7 to 28)
≥ 60 years, median (IQR)	13 (8 to 26)	7 (3.5 to 14)	11 (7 to 18)
Massive transfusion, No. (%)			
Yes, median (IQR)	44 (30.5 to 64.5)	25 (19 to 38)	32 (23 to 58)
No, median (IQR)	9 (7 to 12)	6 (5 to 10)	8 (6 to 11)
Largest volume enrolling site, No. (%)			
Yes, median (IQR)	11.5 (8 to 25.5)	10 (6 to 23)	11 (7 to 23)
No, median (IQR)	14 (8 to 33)	18 (9 to 26)	17 (8 to 30)
Survival at 6 hours, No. (%)			
Yes, median (IQR)	11 (8 to 31)	10 (6 to 22)	11 (7 to 23)
No, median (IQR)	14 (11 to 30)	28 (13 to 56)	19.5 (11 to 43)
Survival at 24 hours, No. (%)			
Yes, median (IQR)	11 (8 to 31)	10 (6 to 22)	11 (7 to 23)
No, median (IQR)	16 (11 to 36.5)	28 (13 to 56)	18 (11 to 43)

Abbreviations: FC – fibrinogen concentrate, IQR – interquartile range, IU – international unit, PCC – prothrombin complex concentrate, SD – standard difference. Massive transfusion – Defined as 10 or greater units of RBCs within the first 24 hours following admission.

© 2025 da Luz LT et al. *JAMA Network Open*

eTable 4. Trial outcomes by treatment group in the per protocol population

	Plasma (n=65)	FC and PCC (n=60)	LS mean (1-sided 97.5%CI), Odds or Hazard Ratio or Risk Difference (%), 95% CI	*P* value
**Primary outcome**				
Total no. ABPs transfusion within 24h, units	25.4 (20.4 to 31.5)	22.2 (17.7 to 27.8)	LS mean: 0.87 (0.0 to 1.20)	0.202[a]
Mean (SD)	25.4 (26.5)	22.2 (27.1)		
Median (IQR)	14.0 (8 to 32)	12.0 (7 to 24)		
**Secondary outcomes**				
Total no. ABPs transfusion within 24h, w/o plasma as active control, units	20.4 (16.16 to 25.76)	22.2(17.42 to 28.29)	LS mean: 1.09 (0.0 to 1.52)	0.689[a]
Mean (SD)	20.4(25.0)	22.2 (27.1)		
Median (IQR)	10.0 (5 to 26)	12.0 (7 to 24)		
Days out of hospital at day 28, median (IQR)	0 (0 to 4)	0 (0 to 10)		0.368
ICU-free days at day 28, median (IQR)	8 (0 to 19)	11 (0 to 20)		
Ventilator-free days at day 28, median (IQR)	13 (0 to 22) (n=63)*	16 (0 to 23.5) (n=57)*		0.39
24-hour mortality	12 (16.9)	5 (7.6)		0.24 [b]
All-cause mortality at 28 days	15 (23.08)	8 (13.33)	HR: 0.58 (0.26 to 1.26)	0.161 [c]

Abbreviations: ABP – allogeneic blood products, FC – fibrinogen concentrate, HR – hazard ratio, ICU – intensive care unit, IQR – interquartile range, LS – least square, OR – odds ratio, PCC – prothrombin complex concentrate, RBC – red blood cells, RD – risk difference, SD – standard difference.

a Least Square Ratio (FC + PCC/plasma) 1-sided 97.5% CI.

b Log Rank Test for comparison between groups.

c Cox Proportional Hazard Model used for comparison between time to death within 28 days.

d Fisher's Exact Test p value.

* Analysis of subjects 02-003 (Octaplex & Fibryga), 04-005 (Octaplex & Fibryga), 04-011 (Frozen Plasma), 04-017 (Octaplex & Fibryga), 04-019 (Frozen Plasma) was not possible due to missing start/end date of ICU.

© 2025 da Luz LT et al. *JAMA Network Open*

eFigure 1. Transfusion of blood products across the first 24 hours following admission, mITT N=137:

A) ABPs B) RBCs C) Plasma D) Platelets

A)

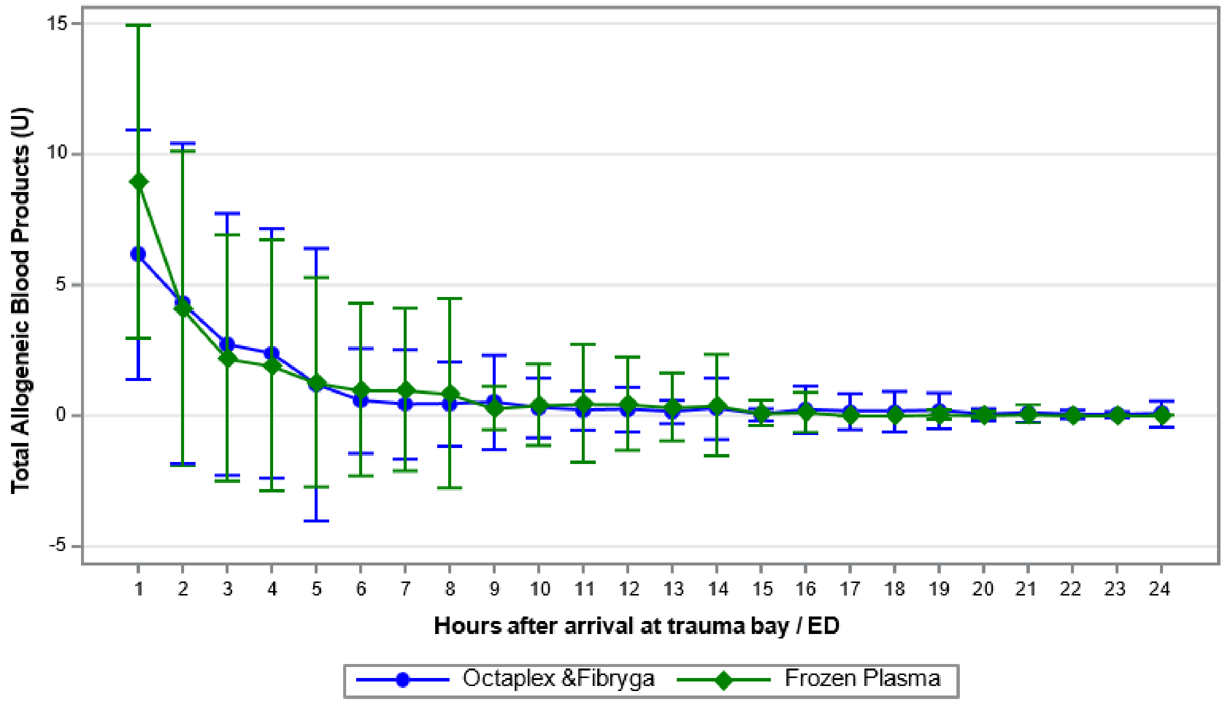

B)

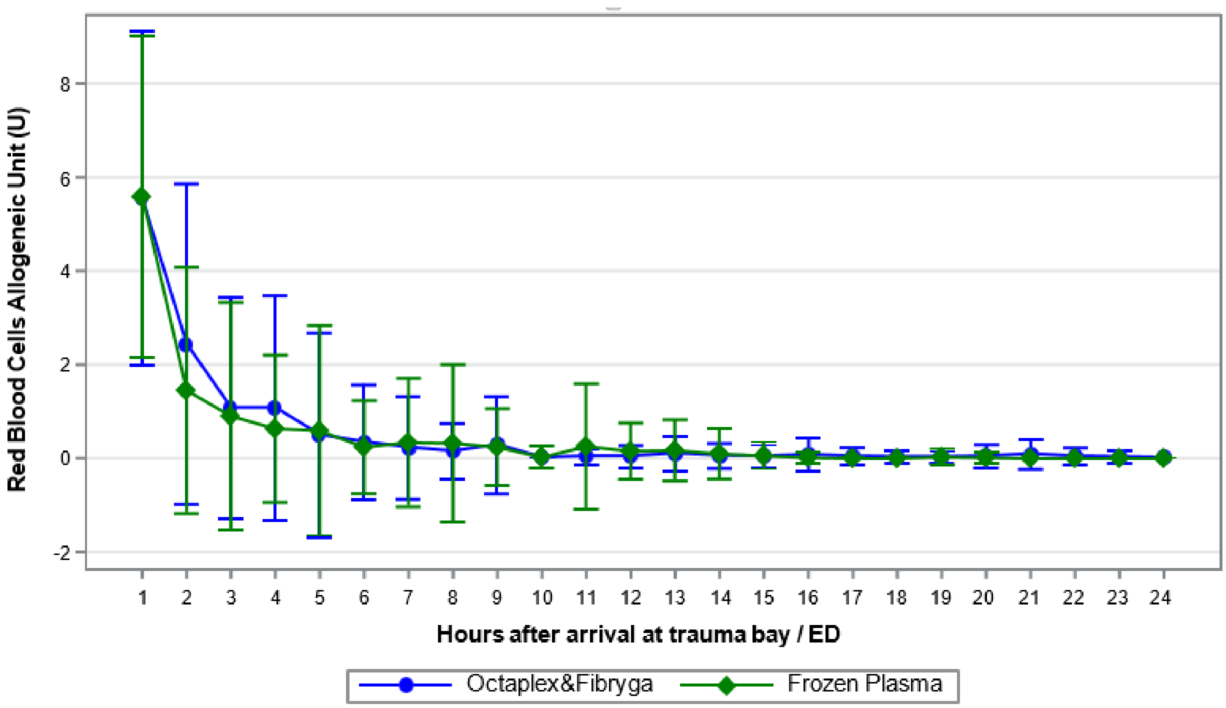

© 2025 da Luz LT et al. *JAMA Network Open*

C)

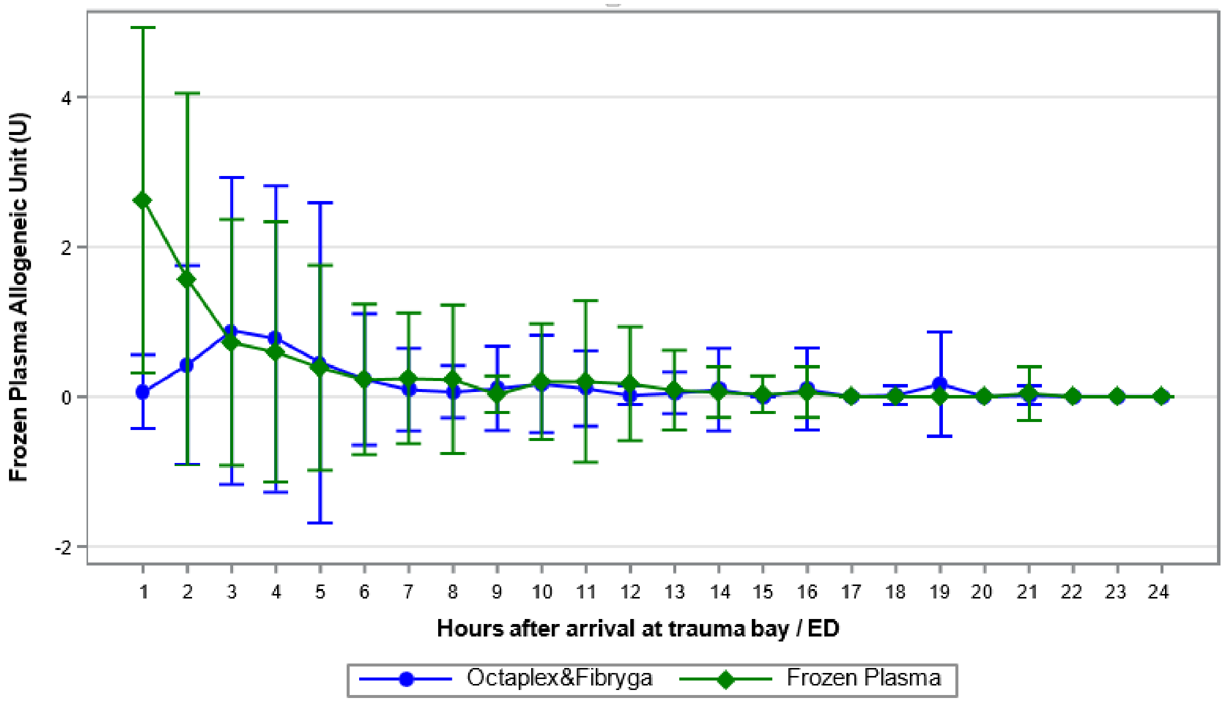

D)

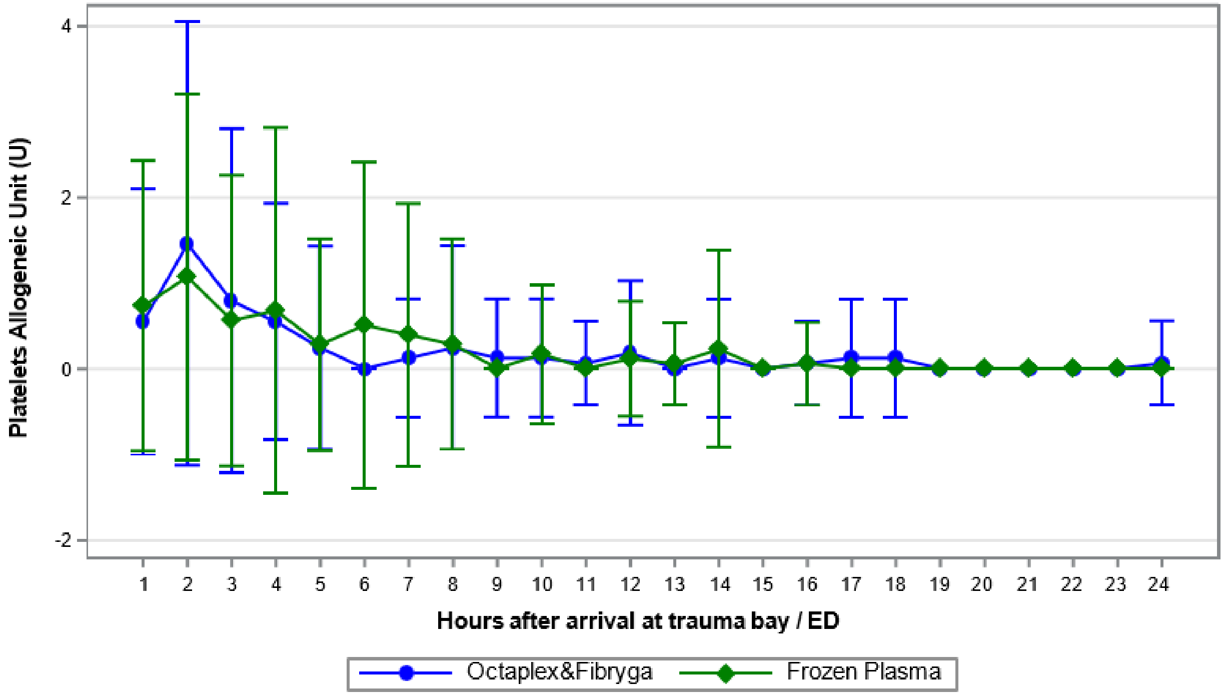

Abbreviations: ABP– allogeneic blood product, RBC – red blood cell, INR– international normalized ratio

© 2025 da Luz LT et al. *JAMA Network Open*

eFigure 2. Coagulation profile and hemoglobin across the first 24 hours following admission, mITT N=137

A) INR B) Fibrinogen C) Platelets D) Hemoglobin

A)

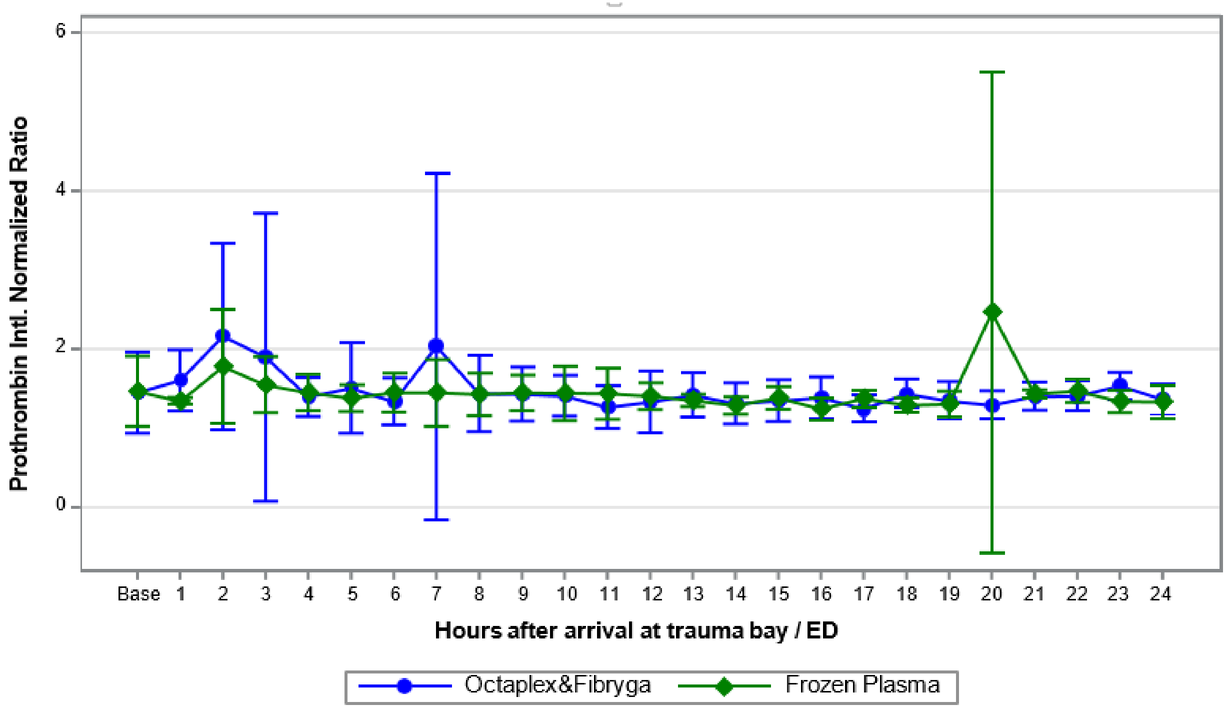

B)

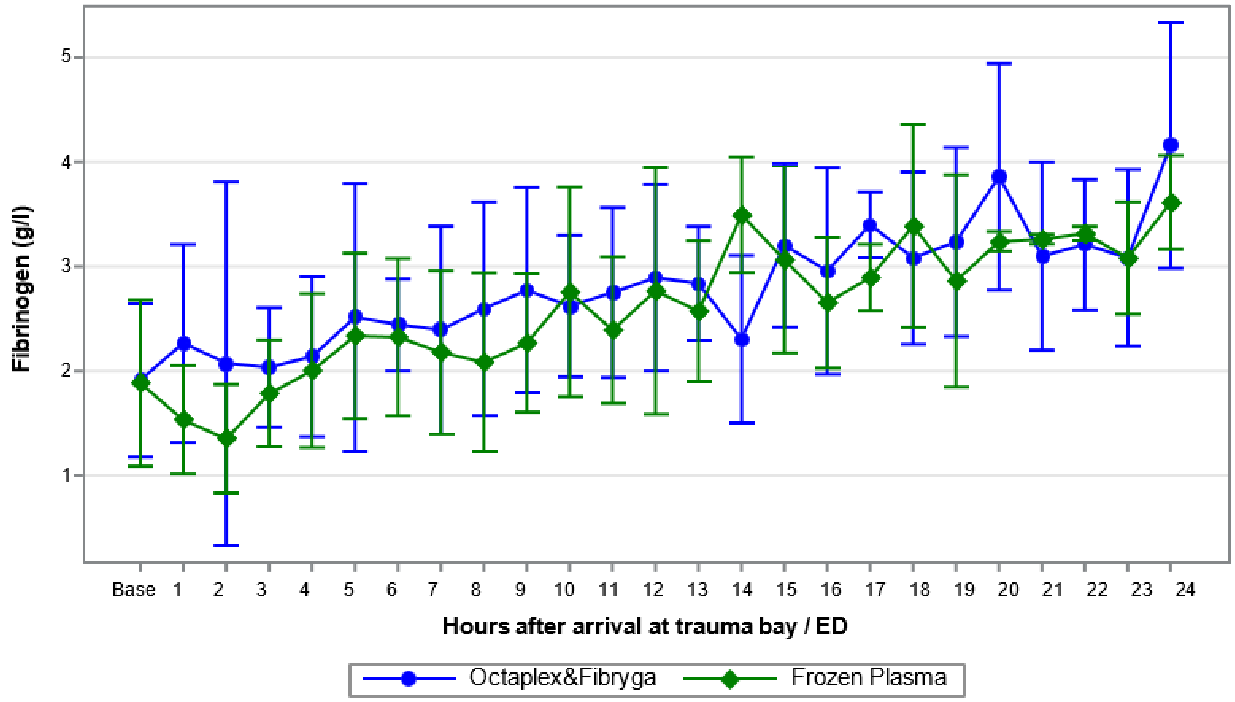

© 2025 da Luz LT et al. *JAMA Network Open*

C)

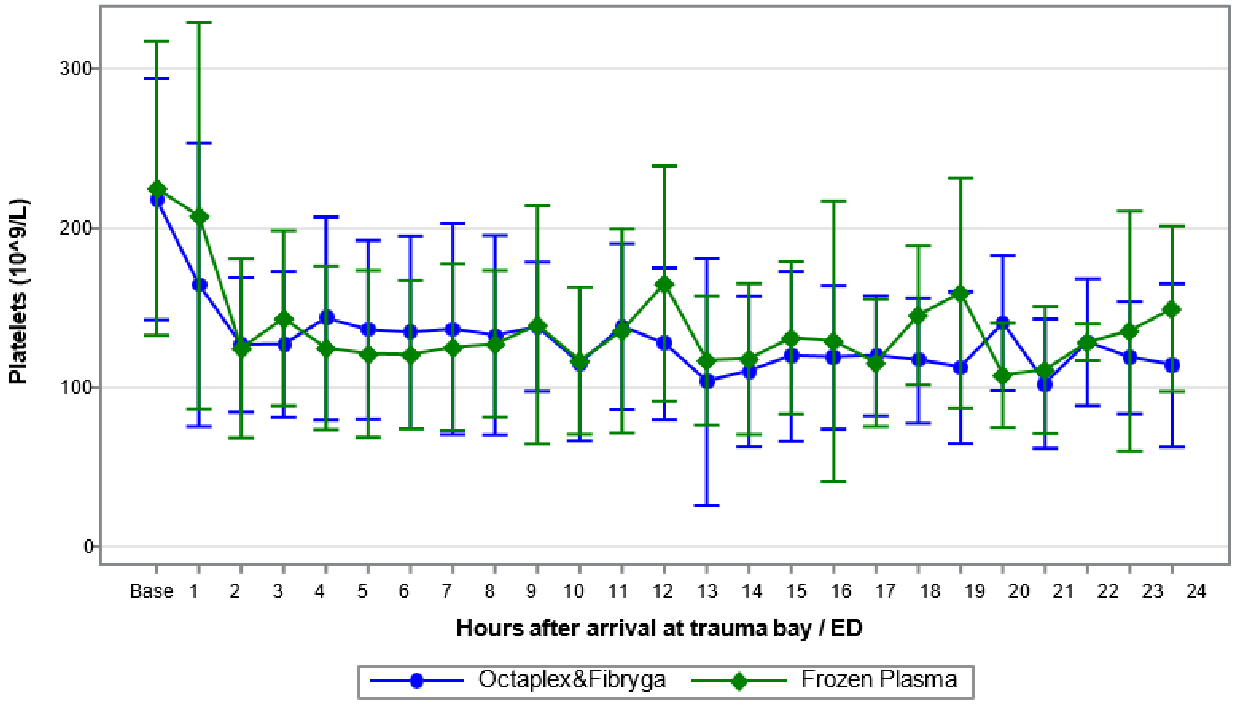

D)

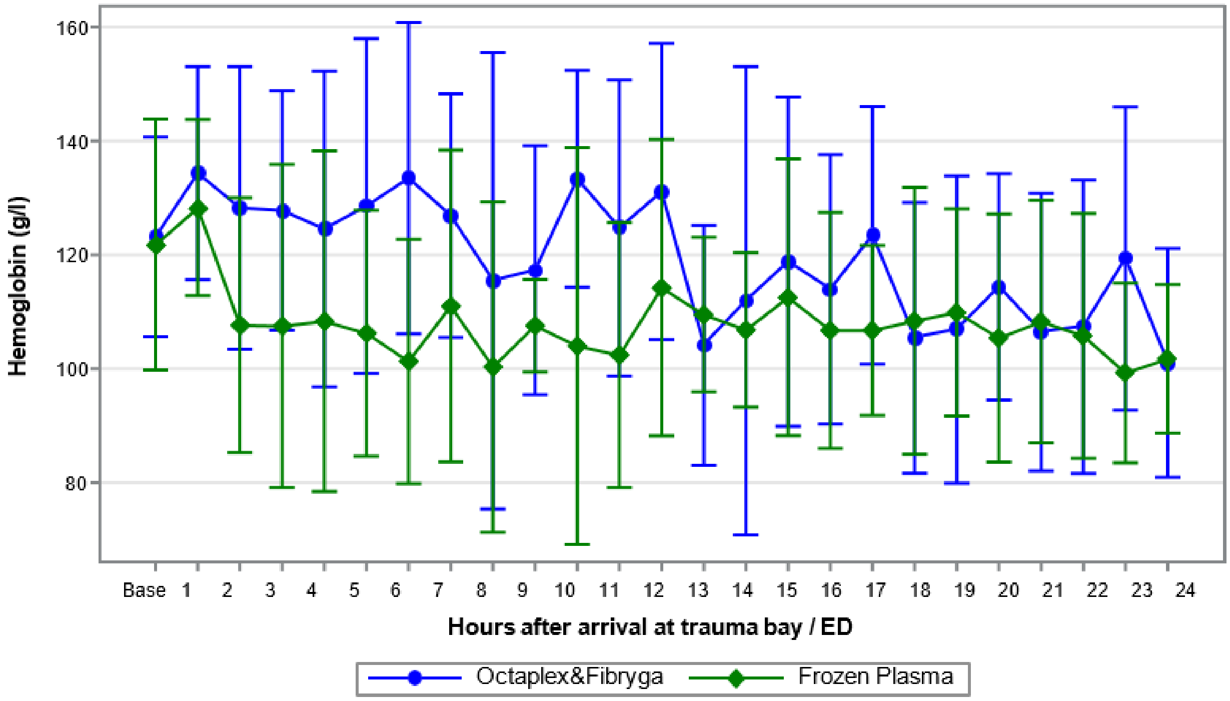

© 2025 da Luz LT et al. *JAMA Network Open*
