# Supplementary material for: Factors in the Initial Resuscitation of Patients With Severe Trauma: The FiiRST-2 Randomized Clinical Trial
Source: JAMA Netw Open. 2025 Sep 22;8(9):e2532702. doi: 10.1001/jamanetworkopen.2025.32702 (PMC12455389; doi:10.1001/jamanetworkopen.2025.32702)
Supplement: Supplement 4. — Data Sharing Statement [file jamanetwopen-e2532702-s004.pdf]

Data Sharing Statement

da Luz. Factors in the Initial Resuscitation of Severe Trauma. *JAMA Netw Open*. Published September 22, 2025. doi:10.1001/jamanetworkopen.2025.32702

Data
Additional Information: https://clinicaltrials.gov/study/NCT04534751?cond=Trauma%20Injury&intr=clotting%20factor%20concentrates&rank=3 Registration number: NCT04534751
Data available: No
